# UDP-glycosyltransferases act as key determinants of host plant range in generalist and specialist *Spodoptera* species

**DOI:** 10.1073/pnas.2402045121

**Published:** 2024-04-29

**Authors:** Huidong Wang, Jing Song, Benjamin J. Hunt, Kairan Zuo, Huiru Zhou, Angela Hayward, Bingbing Li, Yajuan Xiao, Xing Geng, Chris Bass, Shutang Zhou

**Affiliations:** ^a^State Key Laboratory of Cotton Bio-breeding and Integrated Utilization, School of Life Sciences, College of Agriculture, Henan University, Kaifeng 475004, Henan, China; ^b^Centre for Ecology and Conservation, University of Exeter, Penryn TR10 9FE, United Kingdom

**Keywords:** Spodoptera pests, UDP-glucosyltransferases, host adaptation, DIMBOA, xenobiotic detoxification

## Abstract

Phytophagous insects have evolved sophisticated detoxification systems to overcome the antiherbivore chemical defenses produced by many plants. However, how these biotransformation systems differ in generalist and specialist insect species and their role in determining insect host plant range remains an open question. Here, we show that UDP-glucosyltransferases (UGTs) play a key role in determining the host range of insect species within the *Spodoptera* genus. Comparative genomic analyses of *Spodoptera* species that differ in host plant breadth identified a relatively conserved number of UGT genes in generalist species but high levels of UGT gene pseudogenization in the specialist *Spodoptera picta*. CRISPR-Cas9 knockouts of the three main UGT gene clusters of *Spodoptera frugiperda* revealed that UGT33 genes play an important role in allowing this species to utilize the poaceous plants maize, wheat, and rice, while UGT40 genes facilitate utilization of cotton. Further functional analyses in vivo and in vitro identified the UGT SfUGT33F32 as the key mechanism that allows generalist *S. frugiperda* to detoxify the benzoxazinoid DIMBOA (2,4-dihydroxy-7-methoxy-2H-1,4-benzoxazin-3(4H)-one), a potent insecticidal phytotoxin produced by poaceous plants. However, while this detoxification capacity is conserved in several generalist *Spodoptera* species, *Spodoptera picta*, which specializes on *Crinum* plants, is unable to detoxify DIMBOA due to a nonfunctionalizing mutation in *SpUGT33F34*. Collectively, these findings provide insight into the role of insect UGTs in host plant adaptation, the mechanistic basis of evolutionary transitions between generalism and specialism and offer molecular targets for controlling a group of notorious insect pests.

## Significance

The genetic factors that influence diet breadth in herbivorous insects remain poorly understood. Here, we demonstrate that uridine diphosphate (UDP)-glycosyltransferase (UGT) genes play a key role in the capacity of species within the *Spodoptera* genus of insects to utilize certain host plants and thus act as important determinants of host range. We uncover a conserved UGT-mediated mechanism of plant defense chemical detoxification in generalist *Spodoptera* species that allows them to utilize maize, wheat, and rice. However, this mechanism has been lost in the specialist *Spodoptera picta* through mutation, rendering it unable to feed on these plants. Our findings provide insights into the molecular innovations required for ecological adaptation and the role of gene gain and loss in determining insect host range.

Many plants produce chemical defense compounds, plant secondary metabolites (PSMs), to protect themselves against herbivory. Phytophagous insects have, in turn, evolved sophisticated biotransformation systems to detoxify these antiherbivore defenses. While polyphagous herbivores have to deal with a broad range of PSMs from plants belonging to different taxa, mono-, and oligophagous insects encounter a comparatively reduced diversity of PSMs in their restricted diets. These differences are thought to have shaped the detoxification gene families of generalist and specialist insect species (1). However, our understanding of the nature of these differences remains surprisingly poorly understood. For example, whether quantitative differences in detoxification gene number, or qualitative changes in the substrate specificity/promiscuity of enzymes encoded by these genes, determines insect host range remains an open question. Furthermore, given the prevailing paradigm that evolutionary arms races between hosts and herbivores leads to increasing specialization (2, 3), the fate of detoxification genes as generalist insects transition to specialism remains unclear.

Insect detoxification of xenobiotics such as PSMs typically involves three phases. These comprise oxidation, hydrolysis, and/or reduction reactions by phase I enzymes such as cytochrome P450s or carboxylesterases, conjugation reactions by phase II enzymes mediated by UDP-glucosyltransferases (UGTs) or glutathione S-transferases, and phase III excretion by transporters (4–6). While our understanding of the importance of many of the enzyme families in insect xenobiotic detoxification is mature, the role of UGT enzymes in insect detoxification of potentially harmful xenobiotics, including PSMs, is comparatively much less well understood.

The UGT superfamily of enzymes has been shown to conjugate an exceptionally diverse range of small lipophilic xenobiotics or endobiotics with sugars including glucuronic acid (most commonly used in mammals), glucose (preferred in arthropods), xylose, N-acetylglucosamine, or galactose (7–9). In insects, the number of genes encoding UGTs varies significantly among different insect species from 5 UGT genes in bed bugs to 106 UGT genes in the glassy-winged sharpshooter (10). Remarkably, even different insect species within the same genus can exhibit marked diversity in UGT gene number suggesting this is a highly dynamic gene family. For example, in the *Drosophila* genus UGT gene number varies from 29 in *Drosophila elegans*, *Drosophila pseudoobscura*, and *Drosophila mojavensis*, to 50 in *Drosophila takahashii* (11). This diversity in gene number is consistent with the finding that several insect UGT family genes exhibit evidence of lineage-specific gene family expansions or “gene blooms” (12, 13). The UGT33 and UGT40 families of the *Spodoptera* genus serve as canonical examples of UGT blooms in insects and are characterized by gene clusters resulting from sequential tandem gene duplication events. It is believed that coevolution between herbivorous insects and plants is the primary driving force behind the extensive amplification of UGT genes in insects (14). Related to this, previous research has discovered specific UGT genes potentially involved in the detoxification of specific PSMs (15–18). However, the role of this gene family in determining insect host range remains poorly understood.

**Fig. 1.**
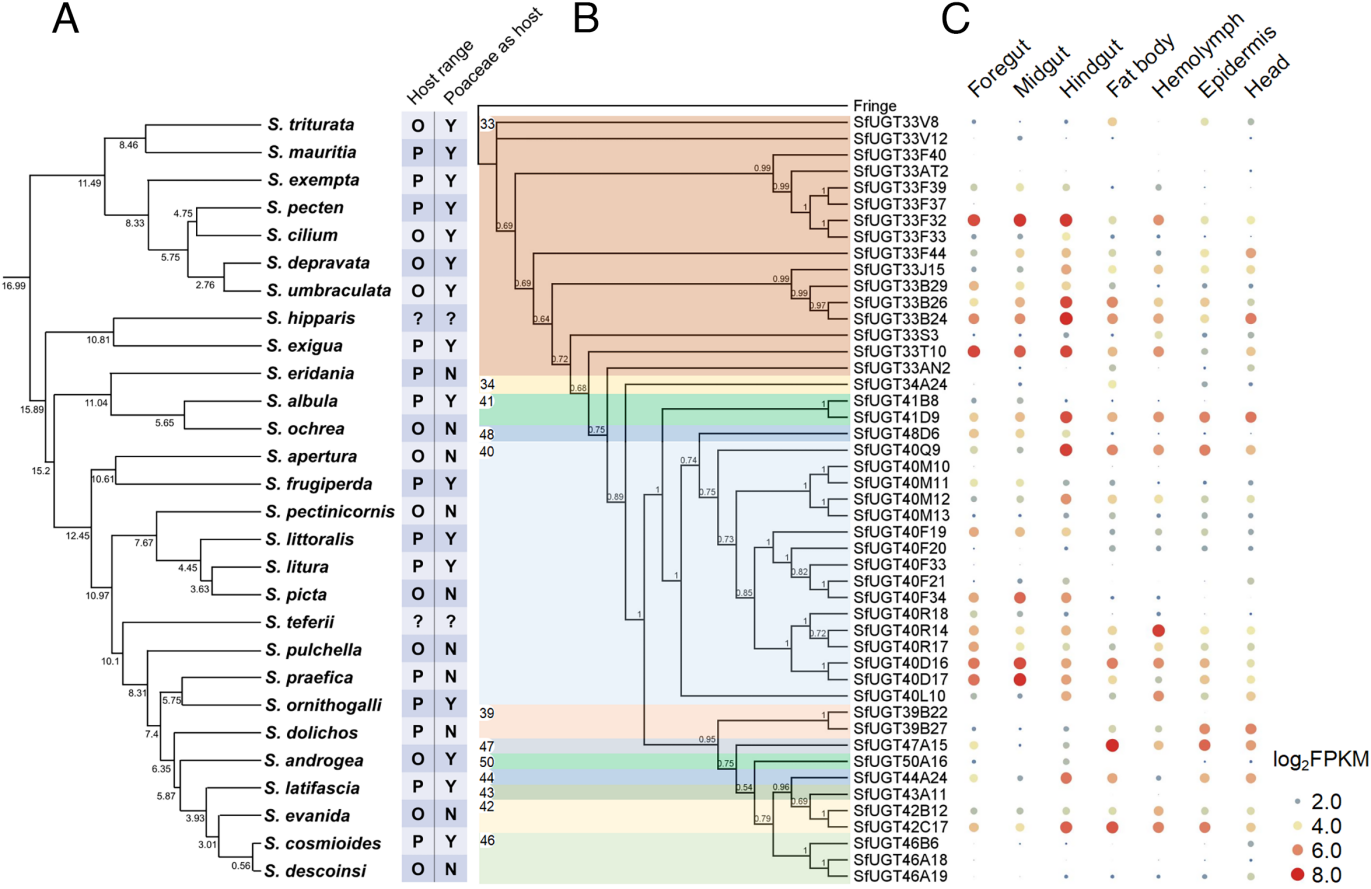
Phylogenetic analysis of species in the *Spodoptera* genus and phylogenetic and transcriptomic analysis of UGTs of *S. frugiperda.* (*A*) Phylogeny of species in the *Spodoptera* genus and information on their host plant range. The species tree and divergence time were derived from ref. 19, median ages (Mya) are provided under the nodes. 95% highest posterior density of estimated ages can be found in ref. 19. The “O” and “P” represent oligophagous and polyphagous species respectively. The “Y” (Yes) and “N” (No) indicate *Spodoptera* species that can or cannot utilize poaceous host plants. Further data on host plant range is provided in Dataset S1. (*B*) Phylogeny of UGTs of *S. frugiperda*. UGTs were aligned with the outgroup Fringe in Geneious version 9.0.5 using MUSCLE. Phylogeny was estimated using Bayesian inference (substitution model, LG + G; chain length, 1,100,000; subsampling frequency, 200; heated chains, 4; burn-in length, 100,000; heated chain temperature, 0.2). Posterior probability is shown above nodes. (*C*) Tissue-specific transcriptomic profiling of *S. frugiperda* UGT genes. Ten individuals were pooled as a biological replicate and three biological replicates were performed for each tissue. Sequencing was performed on the DNBSEQ gene sequencing platform. The heat map was generated using log_2_FPKM (Fragments Per Kilo-base of gene per Million mapped fragments) values. The color and size of circles indicate the expression level of each UGT gene in each tissue.

The genus *Spodoptera* of the family Noctuidae comprises at least 31 species with varied host plant breadths, including examples of oligophagous species such as *Spodoptera picta* and polyphagous species such as *Spodoptera frugiperda*, *Spodoptera exigua*, *Spodoptera* *litura,* and *Spodoptera littoralis.* This diversity in host plant range, and the relatively recent divergence time of species within this genus (0.56 Mya to 16.99 Mya) (Fig. 1*A*) (19), makes the *Spodoptera* genus an exceptional system to study the relationship between detoxification enzyme evolution and host plant adaptation. Furthermore, many generalist *Spodoptera* species are important agricultural or horticultural pests, offering the opportunity to translate knowledge gained on the genetic underpinning of host plant utilization into applied interventions. For example, the fall armyworm, *S. frugiperda*, is a highly invasive species that has recently spread to Africa, Asia, Europe, and Oceania from its native distribution in the Americas and become a destructive global agricultural pest (https://www.cabidigitallibrary.org). *S. frugiperda* is such an economically important pest in part due to its ability to feed on a wide range of host plants including 353 host plant species from 76 families, although it exhibits a preference for plants in the Poaceae family (20). Previous studies have demonstrated that a UGT in this species, SfUGT33F28, metabolizes DIMBOA (2,4-dihydroxy-7-methoxy-2H-1,4-benzoxazin-3(4H)-one), the main PSM in maize, wheat, and some other poaceous plants, in vitro. However, silencing of *SfUGT33F28* by RNAi only reduced the performance of one of the two *S. frugiperda* strains tested on maize, raising questions as to the overall importance of this gene for utilization of this plant as a host (16).

Here, we leveraged the *Spodoptera* system in combination with comparative genomics and CRISPR-Cas9 genome editing to investigate the role of UGT genes in host plant adaptation and host plant range in insects. More specifically, our analyses focused on addressing the following key questions: i) Are qualitative and quantitative changes in UGT genes, as exemplified by members of the UGT33 and UGT40 families, associated with host plant range in generalist and specialist species of the *Spodoptera* genus? ii) Do UGT33 and UGT40 genes play roles in the utilization of poaceous and nonpoaceous host plants in *Spodoptera frugiperda* and the detoxification of PSMs produced by these plants? iii) To what extent are UGT-based detoxification mechanisms conserved in generalist and specialist *Spodoptera* species. iv) Can study of UGT evolution in the *Spodoptera* genus provide insight into the fate of UGT genes as generalist insect species transition to specialism?

## Results

### Characterization of the UGT Superfamily of *S. frugiperda*.

We first leveraged recently released chromosome-scale reference genomes of *S. frugiperda* to characterize the complement of UGTs in this species. A total of 48 UGT genes were identified from the genome of the Faw-zju isolate (NCBI accession no. GCA_011064685.2) (21) and 49 UGT genes from the SF20-4 isolate (NCBI accession no. GCA_023101765.3) based on BLAST searches using UGT genes of *Helicoverpa armigera* and *Bombyx mori* as query sequences (*SI Appendix*, Figs. S1 and S2). Manual curation of these sequences and removal of duplicated or incorrectly assembled/annotated genes resulted in a final set of 47 nonredundant UGT genes that encode conserved structural features that are characteristic of this gene family (*SI Appendix*, Fig. S3). The sequence of all 47 UGT genes were verified by PCR and Sanger sequencing in the XZ strain of *S. frugiperda* used in this study and named by the UGT Nomenclature Committee (Dataset S2).

Phylogenetic analysis of the 47 *S. frugiperda* UGTs grouped them into 12 families comprising the 33, 34, 39, 40, 41, 42, 43, 44, 46, 47, 48, and 50 families (Fig. 1*B*). Of these, the UGT33 and UGT40 families were the largest with 16 members each, while the other families possess 3 or less UGT genes. To investigate the genomic distribution of *S. frugiperda* UGTs and gain insight into the expansion of the UGT33 and UGT40 families, the 47 UGTs were mapped to nine chromosomes (*SI Appendix*, Figs. S1 and S2). UGT genes belonging to the UGT33 family were found on three chromosomes, single gene members on chromosome 11 (*SfUGT33T10*) and 25 (*SfUGT33AN2*), and two gene clusters on chromosome 27 containing ten and four head to tail UGT33 genes respectively. UGT40 gene family members were all found on chromosome 25 comprising a large cluster of 15 UGT40 genes and a single UGT gene (*SfUGT40Q9*) separated from this cluster (*SI Appendix*, Fig. S1). Analysis of the exon-intron structure of the 47 UGT genes revealed an identical exon-intron structure in UGT genes belonging to the same family (*SI Appendix*, Fig. S4), which is in accord with their evolutionary relationship. The largest UGT33 and UGT40 families all possess three and seven introns in the coding region of the gene respectively. Collectively, these data provide evidence that the UGT33 and UGT40 families have expanded in *S. frugiperda* by tandem duplication followed by diversification.

The tissue-specific expression pattern of UGT genes in *S. frugiperda* was investigated using RNAseq data generated for seven specific tissues/body parts (foregut, midgut, hindgut, fat body, hemolymph, epidermis, and head) from sixth instar larvae (Fig. 1*C*). The expression of certain UGT genes (*SfUGT33F40*, *SfUGT33F37*, *SfUGT40M10*, *SfUGT40F33*, *SfUGT39B22,* and *SfUGT43A11*) was undetectable or detected at only extremely low levels in any tissue. In contrast, many UGT genes appear to be expressed ubiquitously, with high expression levels often seen within the digestive and detoxification systems (gut or fat body), particularly for some members of the UGT33 and UGT40 families (*SfUGT33F32*, *SfUGT33B26*, *SfUGT33B24*, *SfUGT33T10*, *SfUGT40D16, SfUGT40D17,* and *SfUGT40F34*). Notably, most members of the UGT33 family are expressed at higher levels in the gut and fat body compared to other tissues. Thus, the spatial expression patterns of UGTs in the UGT33 and UGT40 families support important roles in detoxification. Beyond the UGT33 and UGT40 family genes, several other UGT family genes, including *SfUGT41D9*, *SfUGT47A15*, *SfUGT44A24*, and *SfUGT42C17*, exhibit high expression levels in the gut or fat body, which also suggests a potential role in xenobiotic detoxification and host adaptation (Fig. 1*C*).

### Genomic Organization and Phylogeny of the UGT33 and UGT40 Family among Five *Spodoptera* Species.

To compare the evolution of UGT genes in generalist and specialist species of the *Spodoptera* genus we first generated a reference genome for the specialist species *S. picta*. Long and short read sequence data were used to generate a high-quality genome assembly for this species (*SI Appendix*, Table S4). RNAseq data generated for *S. picta* were used to inform the annotation of a total of 14,578 genes in the genome assembly. This genomic resource was then used in combination with publicly available genome data for four other *Spodoptera* species, *S. exigua*, *S. frugiperda*, *S. litura*, and *S. littoralis*, to investigate the genomic organization and phylogenic relationship of the UGT33 and UGT40 family.

**Fig. 2.**
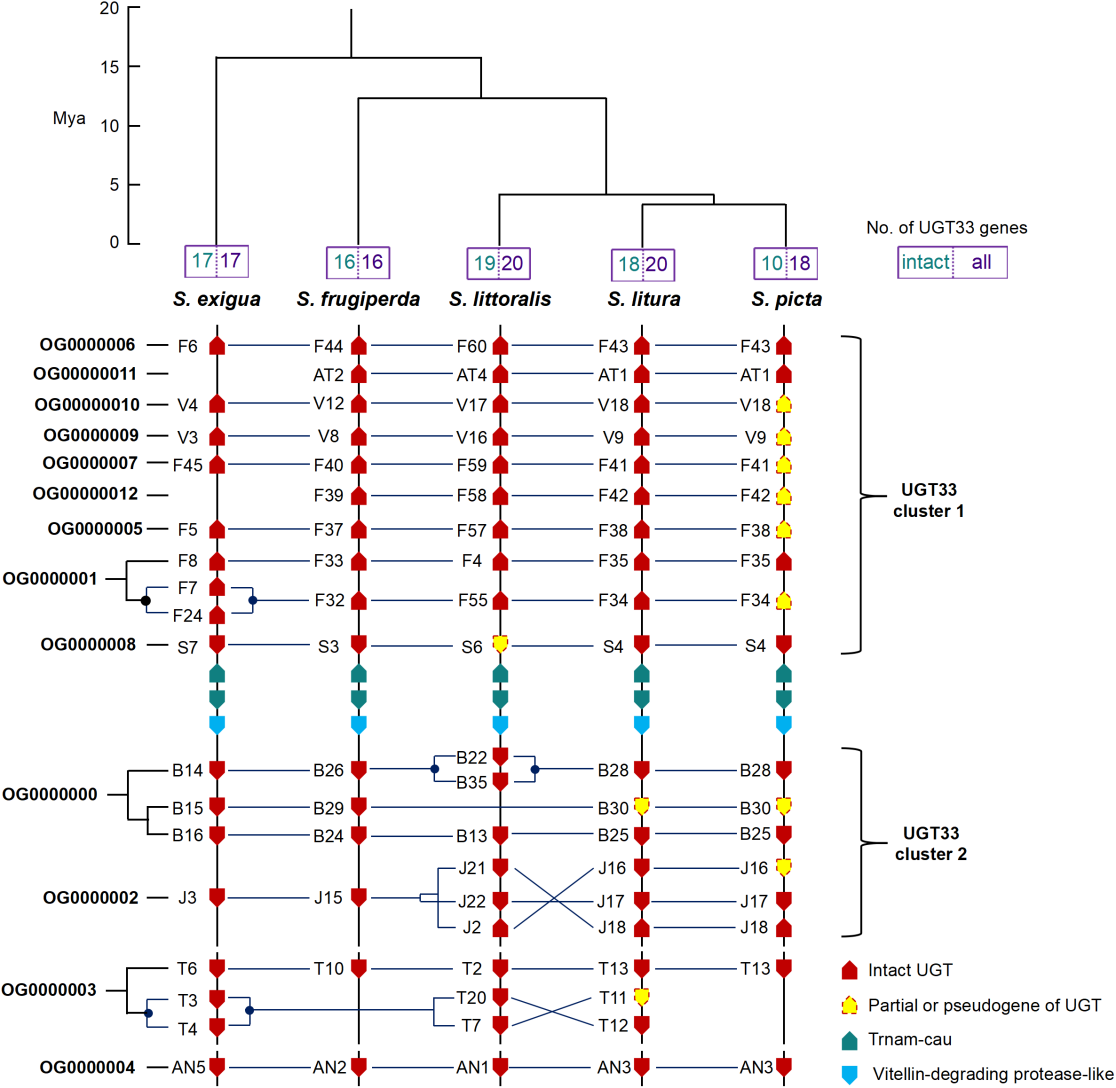
Syntenic analysis of UGT33 family genes in five *Spodoptera* species. The species tree and divergence time (Mya) were derived from ref. 19. UGT genes are shown in their correct orientation and order but physical distances are not to scale. Gene duplications within species are indicated with ●. The red arrows indicate UGT genes with intact sequences, the dashed arrow filled in yellow indicate partial or pseudogene UGTs. OG0000000-0000012 are orthogroups (a set of genes that have descended from a single gene from the last common ancestor in a clade of species) estimated by OrthoFinder (22).

A total of 16 to 20 UGT33 and 14 to 18 UGT40 genes were identified in the five *Spodoptera* species (Fig. 2 and *SI Appendix*, Fig. S5). To gain insight into the evolution of the UGT33 and UGT40 family genes during the speciation of the five *Spodoptera* species, OrthoFinder (22) and NOTUNG (23) were used to characterize gene gain/loss events of the UGT genes at each node of the phylogenetic tree. These analyses revealed highly dynamic changes in the number of UGT33 and UGT40 family genes over the evolution of *Spodoptera*. For the UGT33 family, thirteen orthogroups (Fig. 2) and 23 common ancestral genes (*SI Appendix*, Fig. S6) spanning the five *Spodoptera* species were predicted by OrthoFinder and NOTUNG respectively. Ten (Fig. 2) and four (*SI Appendix*, Fig. S6) 1:1:1:1:1 orthologs were identified across the five species by OrthoFinder and NOTUNG respectively, as higher numbers of gene loss events were predicted by NOTUNG. At least three gene duplication events after the species split were identified by both OrthoFinder and NOTUNG, these were *SeUGT33F7* and *SeUGT33F24*, *SeUGT33T3,* and *SeUGT33T4* in *S. exigua* and *SlittUGT33B22* and *SlittUGT33B35* in *S. littoralis* (Fig. 2 and *SI Appendix*, Fig. S6). Gene loss events were frequent in the evolution of UGT33 family, such as the “missing” orthologs of *SfUGT33AT2*, *SfUGT33F39* in *S. exigua* and *SlituUGT33B30* in *S. littoralis*. In pairwise comparisons of species, the genomes of *S. picta* and *S. litura* contained a very high number of 1:1 UGT33 orthologous genes (Fig. 2). This is consistent with the relatively recent divergence of *S. picta* and *S. litura*, which separated 3.63 Mya (19). Remarkably, however, the coding sequences of eight UGT33 genes of *S. picta* were found to have been disrupted by exon loss or other mutations that result in partial gene sequences or premature termination of translation (Fig. 2 and *SI Appendix*, Fig. S8). PCR amplification and sequencing confirmed that these findings did not represent assembly errors but rather are bona fide nonfunctionalizing mutations. In the case of the UGT40 family, eight orthogroups (*SI Appendix*, Fig. S5) and twenty common ancestral genes (*SI Appendix*, Fig. S7) spanning the five *Spodoptera* species were predicted by OrthoFinder and NOTUNG respectively. Ten (*SI Appendix*, Fig. S5) and five (*SI Appendix*, Fig. S7) 1:1:1:1:1 orthologs were identified across the five species by OrthoFinder and NOTUNG respectively. Gene loss events were more apparent than gene gain events, such as the lack of orthologs of *SfUGT40F21* in *S. exigua*, *SeUGT40D29* in *S. frugiperda*, and *SlituUGT40D20*, *SlituUGT40D22,* and *SlituUGT40M24* in *S. picta* (*SI Appendix*, Fig. S5). As observed for the UGT33 family, in addition to the gene loss events identified above, nonfunctionalizing mutations were also identified in three UGT40 genes of *S. picta* (*SI Appendix*, Figs. S5 and S8). The loss of nearly half of the functional UGT33 genes and several UGT40 genes in *S. picta* by pseudogenization is extraordinary and may reflect the lack of a requirement to retain a diverse profile of xenobiotic detoxifying UGTs in a species that specializes on *Crinum* plants (24).

### Knockout of the UGT33 or UGT40 Gene Clusters in *S. frugiperda* Reduces Performance on Certain Host Plants and Increases Sensitivity to Phytochemicals.

**Fig. 3.**
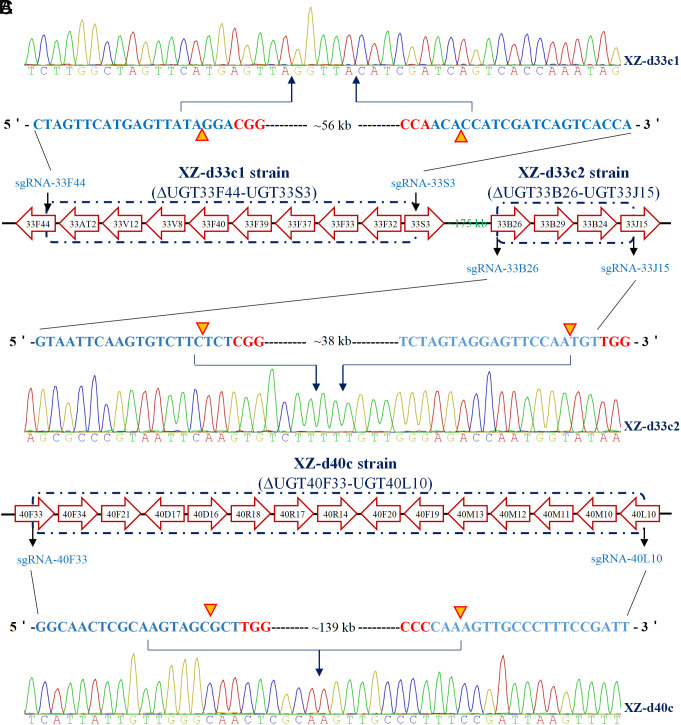
CRISPR-Cas9-mediated knock out of two UGT33 gene clusters (*A* and *B*) and one UGT40 gene cluster (*C*) in *S. frugiperda*. The UGT genes are shown in their correct orientation and order, but their lengths and distances apart are not shown to scale. Target sequences of the sgRNAs (blue), the PAM sequences (red), cutting sites by the Cas9 protein (yellow triangle or inverted triangle), and three representative chromatograms of direct sequencing of PCR products of individuals from the three knockout strains are shown. The genomic fragments (56 kb from 10 UGT33 genes in XZ-d33c1 strain, 38 kb from 4 UGT33 genes in XZ-d33c2 strain, 139 kb from 15 UGT40 genes in XZ-d40c strain) between two sgRNAs designed to target the genes at each end of UGT cluster were deleted as a result of imprecise DNA repair mediated by the nonhomologous end joining (NHEJ) pathway after CRISPR-Cas9 induced DNA breaks.

**Fig. 4.**
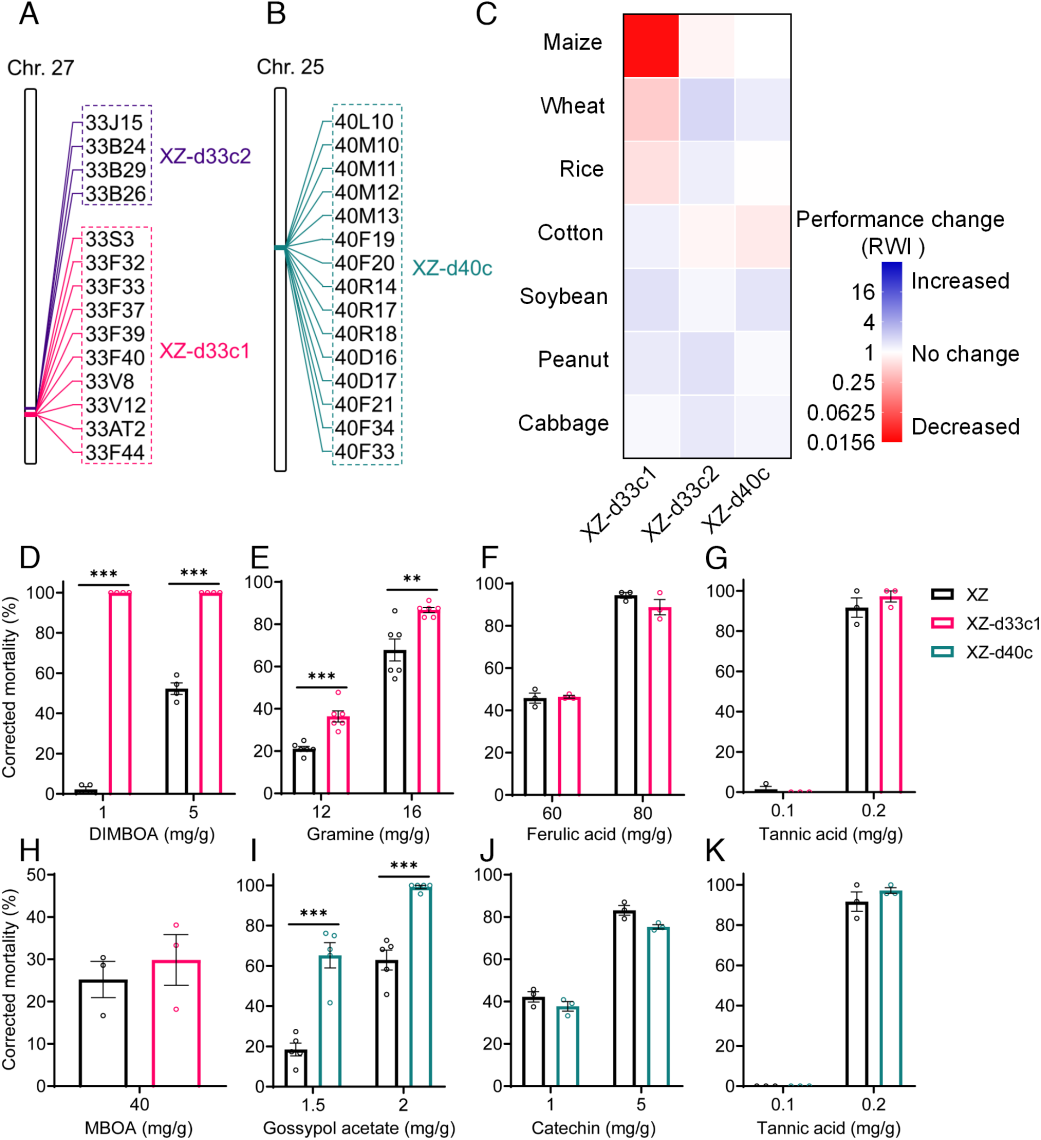
Performance of *S. frugiperda* UGT cluster knockout strains on different host plants and sensitivity to phytotoxins. (*A* and *B*) Schematic of the UGT genes knocked out in the XZ-d33c1, XZ-d33c2, and XZ-d40c strains. (*C*) Performance (relative weight index, RWI) of the XZ-d33c1, XZ-d33c2, and XZ-d40c strains on seven different host plants. RWI = (the weight of knockout strain on plant/average weight of knockout strain on artificial diet)/(average weight of XZ strain on plant/average weight of XZ strain on artificial diet). The red rectangle indicates the decreased performance of knockout strains on a specific plant compared to the XZ strain, and the blue rectangle indicates increased performance. Forty-eight or sixty individuals were tested on each plant or artificial diet for all strains. (*D*–*K*) Sensitivity of the XZ-d33c1, XZ-d33c2, and XZ-d40c strains to seven phytotoxins. Values are means of corrected mortality ± SEM, n = 3 to 6 biologically independent samples, twenty-four larvae were used as a biologically independent sample. Unpaired *t* tests were used for statistical comparisons, **P < 0.01, ***P < 0.001.

To investigate the causal role of the UGT33 and UGT40 families in xenobiotic detoxification and host plant utilization in the *Spodoptera* genus, we selected *S. frugiperda*, as a representative species for initial functional analysis. A dual sgRNA (single guide RNA)-directed CRISPR-Cas9 approach was used to knockout two clusters of UGT33 genes (UGT33 cluster 1, abbreviated as 33c1, containing ten UGT genes comprising *SfUGT33F44* to *SfUGT33S3*, and UGT33 cluster 2, abbreviated as 33c2 containing four genes comprising *SfUGT33B26* to *SfUGT33J15*) and one cluster of UGT40 genes (UGT40 cluster, abbreviated as 40c, containing 15 UGT genes comprising *SfUGT40F33* to *SfUGT40L10*) in the genetic background of the XZ strain (Fig. 2 and *SI Appendix*, Figs. S1 and S4). This resulted in the successful creation of three UGT gene cluster knockout strains (XZ-d33c1, XZ-d33c2, XZ-d40c) (Figs. 3 and 4 *A* and *B* and *SI Appendix*, Fig. S9).

The effect of knockout of the three UGT clusters on the ability of *S. frugiperda* to utilize three Poaceae (maize, wheat, and rice) and four non-Poaceae (cotton, soybean, peanut, and Chinese cabbage) plant species as hosts was examined. Compared with the background XZ strain, the performance [relative weight index (RWI), see *Materials and Methods*] of the XZ-d33c1 strain to maize, wheat, and rice decreased significantly (Fig. 4*C*). In particular, the performance of the XZ-d33c1 strain on maize was a remarkable 53-fold lower than the parental XZ strain (Fig. 4*C*). The performance of the XZ-d33c1 strain on wheat and rice was 2.3-fold and 1.7-fold lower than the XZ strain respectively (Fig. 4*C*). The XZ-d33c2 strain showed a negligible decrease (1.2-fold) in performance on maize compared to the XZ strain (Fig. 4*C*). In the case of the XZ-d40c strain a significant 1.4-fold decrease in performance on cotton compared to the XZ strain was identified (Fig. 4*C*). These results indicate that one or more of the 10 UGT33 genes in the 33c1 cluster are important determinants of the performance of *S. frugiperda* on maize, wheat, and rice, and one or more of the 15 UGT40 genes in the 40c cluster play a role in the performance of this species on cotton.

Given the above findings, and the well-established role of UGT genes in detoxification of PSMs, we next examined the sensitivity of the XZ-d33c1 and XZ-d40c UGT knockout strains to allelochemicals produced by maize, wheat, rice, and cotton. Seven plant toxins [the benzoxazinoids (BXD) DIMBOA and MBOA (6-methoxy-2-benzoxazolinone) primarily produced by maize and wheat (25); gramine produced by rice (26); ferulic acid produced by wheat, rice, and maize (27–29); gossypol and catechin produced by cotton (30, 31); and tannic acid produced by rice and cotton (31, 32)] were selected for use in bioassays. Compared to the background strain XZ, the susceptibility of the XZ-d33c1 strain to a high and low concentration of DIMBOA (Fig. 4*D*) and gramine (Fig. 4*E*) was significantly higher. In the case of XZ-d40c, susceptibility to gossypol acetate was significantly increased compared to the XZ strain (Fig. 4*I*). However, the susceptibility of either UGT knockout strain to ferulic acid, tannic acid, MBOA, and catechin did not differ significantly from the background strain. Thus, these results indicate that one or more UGT genes in the 33c1 cluster enhance performance on specific host plants by enhancing tolerance to DIMBOA and gramine, while one or more UGT genes in the 40c cluster confer tolerance to the gossypol acetate.

### SfUGT33F32-Mediated Glucosylation Was the Key Mechanism Allowing *S. frugiperda* to Detoxify DIMBOA and Utilize Maize as a Host.

**Fig. 5.**
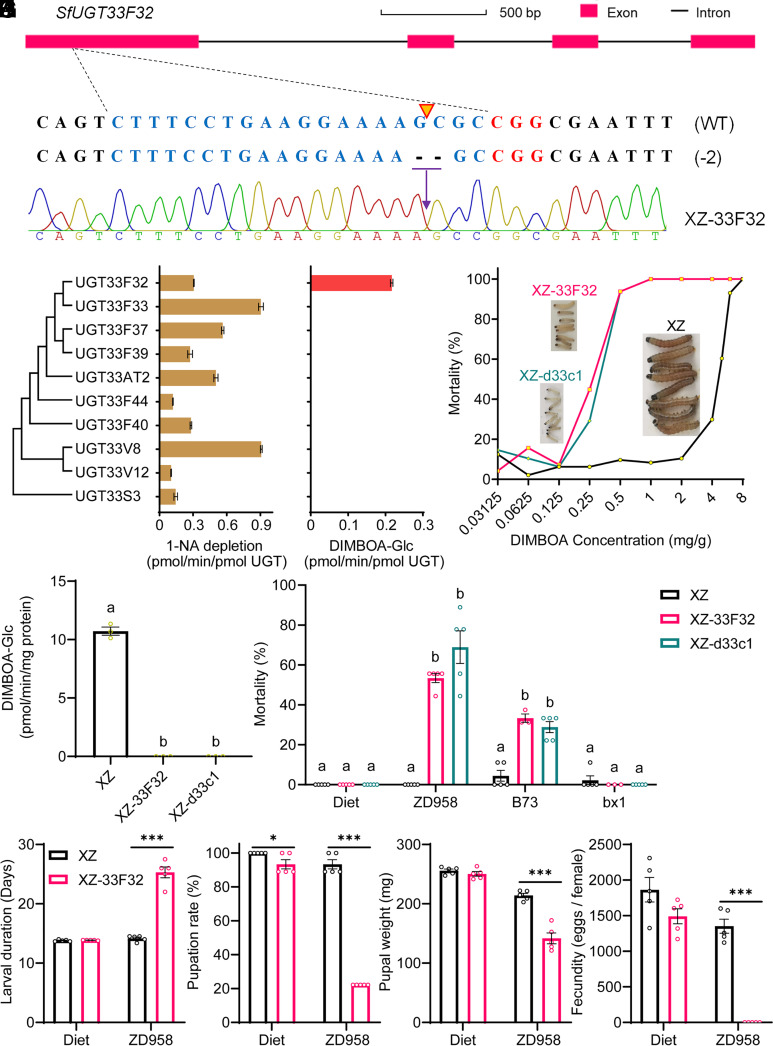
Identification of *SfUGT33F32* as the key UGT that allows *S. frugiperda* to detoxify DIMBOA and utilize maize as a host. (*A*) Schematic of the sgRNA-target sites and recognition sequences in exon 1 of *SfUGT33F32*. The target sequences of the wild-type *SfUGT33F32* allele and the PAM sequences are highlighted in blue and red respectively. The cleavage site is indicated with a yellow inverted triangle. (*B*) A representative chromatogram of direct sequencing of PCR products flanking the sgRNA site of *SfUGT33F32* from the XZ-33F32 strain. XZ-33F32 is homozygous for a 2-bp deletion in exon 1 of *SfUGT33F32*, which would be predicted to produce a truncated and nonfunctional protein. (*C* and *D*) Metabolic activity of 10 recombinant UGT33 proteins of *S. frugiperda* against the model substrate 1-NA and DIMBOA. Metabolic activity is expressed as pmol/min/pmol UGT. Activity is based on substrate 1-NA depletion for 1-NA and DIMBOA-Glc formation for DIMBOA. Error bars represent mean values ± SEM (n = 3). (*E*) Responses of XZ, XZ-33F32, and XZ-d33c1 strain larvae of *S. frugiperda* to DIMBOA. The pictures depict representative images of the three strains after feeding on artificial diet containing 1 mg/g DIMBOA. (*F*) In vitro enzyme activities toward DIMBOA in midguts dissected from XZ, XZ-33F32, and XZ-d33c1 strain larvae, Error bars represent mean values ± SEM (n = 3). (*G*) The mortality of the XZ-33F32, XZ-d33c1, and XZ strains on artificial diet and three maize lines including a commercial maize line Zhengdan958 (ZD958), BXD-deficient maize line (*bx1*) and its genetic background maize line (B73) after 7 d. Error bars represent mean values ± SEM (n = 3 to 5). (*H*–*K*) Performance of the *SfUGT33F32* knockout line and the XZ strain on artificial diet and maize ZD958. Larval duration (*H*), pupation rate (*I*), pupal weight (*J*), and fecundity (*K*) was assessed. Error bars represent mean values ± SEM (n = 5), (*F* and *G*) Significant differences (*P* < 0.05) are denoted using letters above bars as determined by one-way ANOVA with Tukey HSD. (*H* and *K*) unpaired *t* tests were used for statistical significance comparison, *P < 0.05, ***P < 0.001.

*S. frugiperda* is most notorious as a damaging pest of maize worldwide (33, 34). Given this, and the dramatic impact of knockout of one of the UGT33 gene clusters on, a) the ability of *S. frugiperda* to utilize maize as a host and, b) its sensitivity to DIMBOA, the primary PSM produced by this plant as a defense against herbivory, we next focused on identification of the UGT33 gene(s) involved in conferring tolerance to this compound. The ten UGT33 genes found in the 33c1 cluster were functionally expressed in a lepidopteran cell line and the ability of each UGT33 protein to metabolize DIMBOA in vitro was examined. Successful expression of UGT33 proteins was demonstrated by western blot experiments (*SI Appendix*, Fig. S14) and assays using a model substrate 1-naphthol (1-NA) for UGT activity, with recombinant UGT33s exhibiting 1-NA activities of 0.10 ± 0.002 pmol to 0.91 ± 0.008 1-NA depletion/min/pmol UGT (Fig. 5*C*). The formation of DIMBOA glucoside (DIMBOA-Glc) was used as the criterion to assess the metabolic activity of each UGT against DIMBOA. Among the ten *S. frugiperda* UGT33 proteins assayed, only SfUGT33F32 was shown to metabolize DIMBOA with an activity of 0.22 ± 0.003 pmol DIMBOA-Glc/min/pmol UGT protein (Fig. 5*D*). No significant production of DIMBOA-Glc was detected with any of the other remaining UGT33 proteins. This finding provided clear biochemical evidence of the capacity of SfUGT33F32 to metabolize DIMBOA in vitro and identified it as the only UGT gene in the 33c1 cluster with this activity.

To investigate the capacity of SfUGT33F32 to confer tolerance to DIMBOA in vivo we used CRISPR-Cas9 technology to generate a strain (XZ-33F32) homozygous for a 2-bp deletion in exon 1 of *SfUGT33F32* that results in the introduction of a premature stop codon in the coding sequence of this gene (Fig. 5 *A* and *B*). In bioassays, the XZ-33F32 strain exhibited a marked (16.5-fold) increase in sensitivity to DIMBOA compared to the background strain XZ, with the difference in sensitivity between these strains comparable to the enhanced sensitivity of the XZ-d33c1 strain to DIMBOA (14.4-fold) compared to the XZ strain (Fig. 5*E* and *SI Appendix*, Table S2). These results provide clear evidence of the role of SfUGT33F32 in conferring tolerance to DIMBOA in vivo. To provide additional evidence to support this conclusion, midgut enzyme preparations from larvae of the XZ, XZ-33F32 and XZ-d33c1 strains were prepared and tested for their capacity to metabolize DIMBOA to DIMBOA-Glc. Midgut enzyme preparations of the XZ strain showed strong activity against DIMBOA (Fig. 5*F*). In contrast, preparations from XZ-33F32 and XZ-d33c1 showed no detectable activity against this compound (Fig. 5*F*). The finding that knockout of *SfUGT33F32,* or the 33c1 UGT cluster, leads to extinction of the formation of DIMBOA-Glc provides additional evidence that *SfUGT33F32* plays an exclusive role in glucosylating DIMBOA in *S. frugiperda*.

To verify the role of *SfUGT33F32* in allowing *S. frugiperda* to utilize maize as a host, performance assays of larvae of the XZ, XZ-33F32, and d33c1 strains were conducted on artificial diet and three maize lines comprising a commercial maize line ZD958, a benzoxazinoid (BXD)-deficient maize line *bx1* and its wild-type background line B73. No difference in mortality was observed between the three *S. frugiperda* lines when fed on artificial diet or the BXD-deficient maize line *bx1* (Fig. 5*G*). In contrast, the mortality of the XZ-33F32 and d33c1 strains of *S. frugiperda* on the BXDs-containing maize lines (ZD958 and B73) was significantly greater than that of the XZ strain (Fig. 5*G*). Furthermore, when the XZ-33F32 strain was fed on the commercial maize line ZD958 its larval duration was prolonged, pupation rate and pupal weight declined, and fecundity and hatchability were abolished compared to the XZ line of the same genetic background without the *SfUGT33F32* gene (Fig. 5 *H*–*K* and *SI Appendix*, Fig. S13*C*). Thus, these results clearly demonstrate that *S. frugiperda* requires *SfUGT33F32* to survive and reproduce on BXDs-containing maize.

### The Capacity of *Spodoptera* Species to Metabolize DIMBOA Is Dependent on the Presence of a Functional SfUGT33F32 Ortholog.

**Fig. 6.**
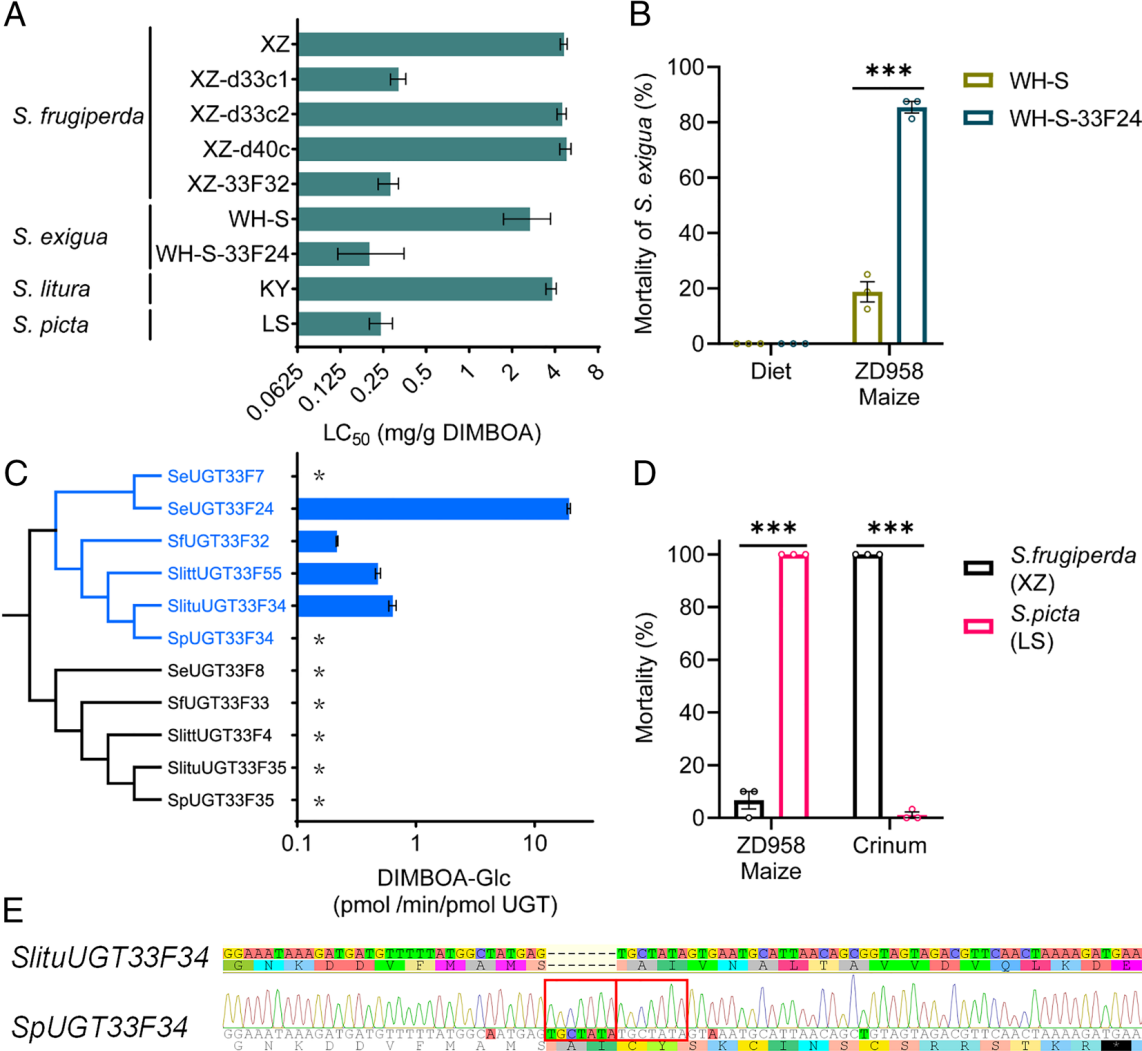
Functional analysis of *SfUGT33F32* and its orthologs in five *Spodoptera* species. (*A*) LC_50_ (Lethal concentration that kills 50% of larvae) estimates of newly hatched larvae of diverse *Spodoptera* strains to DIMBOA. Error bars indicate 95% CI. (*B*) Responses of knockout strains of *SeUGT33F24* (WH-S-33F24) and background strain (WH-S) to a commercial maize line ZD958. (*C*) Metabolic activity against DIMBOA encoded by *SfUGT33F32,* its orthologous genes, and five most closely related paralogous genes in five *Spodoptera* species. Data are mean values ± SEM (n = 3). * indicates no metabolic activity. (*D*) Mortality of *S. frugiperda* and *S. picta* on maize and crinum after 7 d. (*E*) Alignment of a sequenced amplicon of *SpUGT33F34* to *SlituUGT33F34*. *SpUGT33F34* was Sanger sequenced after PCR amplification, red boxes indicate a 7 base pair duplication (TGCTATA). (*B* and *D*) Error bars represent mean values ± SEM (n = 3); unpaired *t* tests were used for statistical significance comparison, ***P < 0.001.

Following the demonstration of the role of *SfUGT33F32* in adaptation to DIMBOA we identified *SeUGT33F24*, *SeUGT33F7*, *SlituUGT33F34*, *SlittUGT33F55,* and *SpUGT33F34* as orthologs of *SfUGT33F32* in *S. exigua*, *S. litura*, *S. littoralis,* and *S. picta,* respectively, based on syntenic and phylogenetic analyses (Figs. 2 and 6*C*). We also selected the five most closely related paralogous genes in the five *Spodoptera* species comprising *SfUGT33F33, SeUGT33F8*, *SlituUGT33F35*, *SlittUGT33F4,* and *SpUGT33F35*. Functional expression of these genes in a lepidopteran cell line revealed that SfUGT33F32 and all the UGT proteins encoded by orthologous genes in other *Spodoptera* species could glycosylate DIMBOA to DIMBOA-Glc with the exception of SeUGT33F7 and SpUGT33F34 (Fig. 6*C*). In contrast, the UGT proteins encoded by paralogous genes exhibited no activity against DIMBOA. To further confirm this finding, we used CRISPR-Cas9 to generate a strain (WH-S-33F24) of *S. exigua* homozygous for a 4-bp deletion in exon 1 of *SeUGT33F24* that results in the introduction of a premature stop codon (*SI Appendix*, Fig. S12 *C*–*F*). We confirmed that no off-target effects of CRISPR-Cas9 editing on *SeUGT33F7* occurred in the WH-S-33F24 strain (*SI Appendix*, Fig. S16). Compared with the background WH-S strain, the LC_50_ values for DIMBOA were significantly decreased in the knockout WH-S-33F24 strain (13.3-fold), similar to the increase in sensitivity (16.5-fold) of the *SfUGT33F32* knockout strain relative to the XZ strain of *S. frugiperda* (Fig. 6*A* and *SI Appendix*, Table S2). Similarly, the mortality of the WH-S-33F24 strain on the commercial maize line ZD958 was 85% compared to 19% for the WH-S strain (Fig. 6*B*), similar to the susceptibility changes after *SfUGT33F32* knockout in *S. frugiperda* on this maize line (Fig. 5*G*). Collectively, these findings reveal a conserved role for specific UGT genes belonging to the *UGT33F* subfamily in metabolizing an important PSM in polyphagous *Spodoptera* species that diverged over tens of millions of years.

As expected, the specialist *S. picta* was not able to survive on the BXDs-containing maize line ZD958 (Fig. 6*D*), and the LC_50_ values of *S. picta* for DIMBOA were equivalent to those of *SfUGT33F32* and *SeUGT33F24* knockout lines of *S. frugiperda* and *S. exigua* respectively (Fig. 6*A* and *SI Appendix*, Table S2). Intriguingly, both the *S. picta* genome assembly and Sanger sequencing after specific *SpUGT33F34* PCR amplification identified a seven bp insertion in exon 1 of this gene that is predicted to lead to premature termination of *SpUGT33F34* translation, effectively making *SpUGT33F34* a pseudogene (Fig. 6*E* and *SI Appendix*, Fig. S8). This is consistent with our finding that the truncated form of SpUGT33F34 cannot metabolize DIMBOA. Thus, the lack of a functional *SfUGT33F32* ortholog in *S. picta* likely underpins the inability of this species to detoxify DIMBOA and thus utilize maize, and other DIMBOA-containing plants, as a host plant.

The finding that the *S. exigua* UGTs SeUGT33F24 and SeUGT33F7 share high levels of sequence identity (*SI Appendix*, Fig. S17 and Table S5) but the former can metabolize DIMBOA while the latter cannot is intriguing and offers an opportunity to investigate candidate determinants of metabolism at the amino acid level. Computational modeling of protein structure and ligand transport analysis suggested that SeUGT33F24 is capable of transporting DIMBOA from the surface to the active part of the enzyme, but SeUGT33F7 is not (Supplementary results, *SI Appendix*, Fig. S19 and Tables S6–S10). Furthermore, four amino acid substitutions (ASN_75_ASP, ASN_82_ASP, ASN_92_THR, and GLU_94_GLY, SeUGT33F7:SeUGT33F24) that are specific to SeUGT33F7 (*SI Appendix*, Figs. S17–S19) and occur in α-helix 3 are predicted to alter the shape of the enzyme at the cleft formed where the N- and C-terminals meet, which accommodates the substrate binding sites (*SI Appendix, Supplementary Results*). Thus, these residues may play an important role in determining DIMBOA metabolism and are excellent candidates for future functional analysis.

## Discussion

Our data demonstrate that UGT genes play a key role in the capacity of species within the *Spodoptera* genus to utilize certain host plants and thus act as important determinants of host plant range. We identify marked differences in the number of functional UGT33 and UGT40 genes in generalist and specialist *Spodoptera* species, and identify a key UGT gene in *S. frugiperda* that effectively detoxifies DIMBOA and allows this species to utilize poaceous plants. We show that this detoxification mechanism is conserved in the generalist *Spodoptera* species examined in this study but has been lost in the specialist *S. picta* through pseudogenization. Our findings provide insights into the molecular mechanisms underpinning ecological adaptation and the role of detoxification genes in evolutionary transitions.

Our analysis of the UGT gene family in *S. frugiperda* revealed a marked expansion of the UGT33 and UGT40 families compared to the remaining 9 UGT families in this species. This finding is consistent with analysis of UGT genes in other Lepidoptera which also identified “blooms” in these two UGT families (9). Related to this, previous studies have suggested that major expansions of several gene families in the *Spodoptera* genus may play a role in polyphagy (35–37). However, whether there is a causal relationship between detoxification gene number and insect host range remains an open question (38–40). Our phylogenetic and syntenic analysis of UGT33 and UGT40 genes in *Spodopter*a species that vary in diet breadth revealed a relatively high degree of conservation in gene number and synteny. However, in the case of the specialist species *S. picta*, while most of the UGT33 and UGT40 genes identified in other *Spodoptera* species were present, a remarkably high number were found to contain nonfunctionalizing mutations. Evolutionary arms races between hosts and herbivores are thought to lead to increasing specialization (2, 3). Our findings are consistent with this and also provide insight into the fate of detoxification genes as generalist insects transition to specialism. Comparison of the specialist *S. picta* and the generalist *S. litura* (which is capable of feeding on over 100 plant species (36) in our study identified a very high number of 1:1 UGT33 orthologous genes and high levels of amino acid similarity between orthologs after mutations disrupting coding sequence in UGT genes of *S. picta* were corrected. This is consistent with the relatively recent divergence of *S. picta* and *S. litura*, which separated from a most recent common ancestor 3.63 Mya (19). The pseudogenization of nearly half of the UGT33 genes of *S. picta* after the split of *S. litura* and *S. picta* demonstrates that detoxification gene superfamilies can undergo profound remodeling during evolutionary transitions between generalism and specialism. This may result from stochastic forces such as genetic drift or from selection to reduce the metabolic cost of expressing detoxification genes that are superfluous to requirements following the transition to a more restricted diet with a reduced diversity of PSMs. The generation of a high-quality genome assembly for *S. picta* in this study provides an excellent resource to examine whether our findings on UGT genes in this species are also observed in other important detoxification gene families such as P450s.

We took advantage of the clustering of UGT33 and UGT40 genes in the genome of *Spodoptera* species to create three lines of *S. frugiperda* where these genes were knocked out using CRISPR-Cas9 genome editing. All three knockout lines were able to survive and reproduce on artificial diet, consistent with a role for UGT33 or UGT40 genes in xenobiotic metabolism rather than key physiological processes in vivo. Bioassays of the knockout lines revealed that one or more UGT33 genes in the 33c1 cluster play an important role in allowing *S. frugiperda* to utilize maize, wheat, and rice, and tolerate the PSMs produced by some of these plants (DIMBOA and gramine), and one or more UGT40 genes in the 40c cluster facilitate utilization of cotton and confer tolerance to gossypol produced by this plant. These findings demonstrate the key role of UGTs in host plant adaptation in *S. frugiperda* and provide additional evidence of their role in allowing herbivorous insects to overcome the antiherbivore defense chemicals produced by plants (15, 17, 41). Further functional characterization in vivo and in vitro demonstrated that a single UGT *SfUGT33F32* gene encodes the capacity to detoxify DIMBOA and allow *S. frugiperda* to utilize maize as a host. The inert glucoside of DIMBOA, (2R)-DIMBOA-Glc, is the most abundant BXD in young maize leaves and is converted into toxic DIMBOA upon herbivory (42). Previous work has shown that DIMBOA is reglucosylated to (2S)-DIMBOA-Glc in the gut of *Spodoptera* species rendering it nontoxic (42). Israni et al. (16) demonstrated that SfUGT33F28 (that is SfUGT33F32 in this study, Dataset S2) and SfUGT40L8 (that is SfUGT40L10 in this study, Dataset S2) can glucosylate DIMBOA to (2S)-DIMBOA-Glc in vitro. However, in the same study, silencing of *SfUGT33F28* by RNAi only reduced the performance of one of the two *S. frugiperda* strains tested on maize, making the overall importance of this gene in utilization of this host plant unclear (16). Our analyses, using stable gene knockouts, demonstrate that *SfUGT33F32* plays an essential role in allowing *S. frugiperda* to effectively utilize maize as a host plant. Furthermore, heterologous expression of individual UGTs and bioassays of our UGT knockout lines of *S. frugiperda* demonstrate that other genes in the UGT33 and UGT40 either do not metabolize DIMBOA or do not significantly alter the performance of this species on maize. This provides clear evidence that *S. frugiperda* relies on a single gene of major effect to detoxify DIMBOA. Furthermore, SfUGT33F32 appears to be the key UGT employed by this species in the detoxification of BXD compounds based on the finding that there was no significant difference in the performance of XZ-33F32 and XZ-d33c1 on BXDs-containing maize lines.

The previous work on *SfUGT33F32* in *S. frugiperda* also identified an ortholog of this gene in *S. littoralis*, referred to as *SlUGT33F28* by the authors and named *SlittUGT33F55* in our study, and showed that it also encodes the capacity to glucosylate DIMBOA in vitro, demonstrating a conserved detoxification capacity in the two *Spodoptera* species. In the current study, we used heterologous expression and genome editing to show that orthologs of *SfUGT33F32* are present in five different Spodoptera species and allow the generalist species, *S. frugiperda*, *S. exigua*, *S. litura* and *S. littoralis*, to detoxify DIMBOA. In the case of *S. frugiperda* and *S. exigua* genome editing provided further evidence of the key role these UGTs play in allowing these species to utilize BXD-producing maize strains. Importantly, however, we also show that the specialist *S. picta* has lost the ability to metabolize DIMBOA because of nonfunctionalizing mutations in *SpUGT33F34*, the ortholog of *SfUGT33F32*. Thus, we reveal a conserved mechanism in generalist *Spodoptera* species that allowed them to utilize poaceous plants that produce DIMBOA. This finding parallels work on other insects/enzyme systems. For example, cytochrome P450s of the *CYP6B* gene subfamily endowed the genus *Papilio* (swallowtail butterflies) with the ability to feed on furanocoumarin-containing plants (43). Similarly, P450s of the 336A family were recently identified as a conserved mechanism that allowed honeybees and other hymenopteran species that diverged over 281 Mya to metabolize alkaloids (44). The loss of the key DIMBOA-metabolizing UGT in *S. picta*, and its resulting high sensitivity to this PSM, is consistent with its specialization on *Crinum* plants which do not produce this PSM. This finding provides a functional demonstration of the outcome of genes that become redundant during the transition from generalism to specialism.

Interestingly, we identified two paralogous genes Se*UGT33F7* and *SeUGT33F24* in *S. exigua* as orthologs of *SfUGT33F32.* As *S. exigua* is basal to *S. frugiperda* in the species phylogeny of the *Spodoptera* genus it is possible that DIMBOA metabolism evolved by gene duplication followed by neofunctionalization, with the ortholog of *SeUGT33F7* subsequently lost following the divergence of the species. Related to this, computational modeling of protein structure and ligand transport analysis suggested that SeUGT33F24 is capable of transporting DIMBOA from the surface to the active part of the enzyme but SeUGT33F7 is not, and identified four amino acids that may act as important determinants of DIMBOA metabolism. Thus, these residues are excellent candidates for future functional analysis.

In the current study, we focused on the role of UGTs in the metabolism of DIMBOA, however, bioassays of our CRISPR-Cas9 knockout lines revealed that one or more genes within the UGT33 family also influence sensitivity to gramine and one or more genes within the UGT40 family confer tolerance to gossypol. Gramine is an alkaloid produced by some poaceous crops including rice (26) and barley (45) as a defense against herbivores. Previous studies reported that knockdown of a glutathione *S* transferase gene *NlGST1-1* in *Nilaparvata lugens* results in increased sensitivity to diets containing gramine (26, 46). However, UGTs have not been previously implicated in the detoxification of this PSM. *S. frugiperda* consists of two biotypes, the “corn strain” with a feeding preference for maize, cotton, and sorghum and the “rice strain” with a feeding preference for rice and various pasture grasses (47). The XZ strain used in this study, which is representative of *S. frugiperda* invading China (48), belongs to the corn-strain based on its nuclear genome but the rice strain based on its mitochondrial genome (based on *Tpi* and *COI* gene genotyping). While this result would assign the XZ strain to the corn strain biotype, we found it was able to survive and reproduce on rice in our lab. Our bioassays of UGT knock out lines demonstrate the role of these enzymes in allowing *S. frugiperda* to detoxify gramine and utilize rice as a host. Further work is required to identify the causal UGT(s) involved. Furthermore, it would be interesting to compare the metabolic activity of UGTs for gramine in the two biotypes of *S. frugiperda* to examine whether UGTs play a role in determining the relative performance of the two strains on rice.

Gossypol is a polyphenolic binaphthyl dialdehyde presenting in the Malvaceae family of plants, most notably cotton (49). UGTs have been previously linked in the detoxification of this PSM in other insects, with two UGTs of the UGT41 and UGT40 families (UGT41B3 and UGT40D1) of *H. armigera* shown to be capable of glycosylating gossypol in vitro (15). Our findings are consistent with this in demonstrating that one or more UGT40 genes of *S. frugiperda* also enhance tolerance to this PSM and play a role in the utilization of cotton. Further work is required to identify the specific UGT(s) involved in detoxification of gossypol in *Spodoptera* species.

The *Spodoptera* genus contains several highly damaging and economically important crop pests. In this regard, our identification of genes that play a key role in allowing *Spodoptera* species to utilize several staple crops has applied implications for the development of controls against these pests. Specifically, we found the susceptibility of *S. frugiperda* and *S. exigua* to DIMBOA was increased 13.3 to 16.5-fold after knockout of *SfUGT33F32* or *SeUGT33F24*. Furthermore, very few larvae survived to adults and no eggs were produced when the *SfUGT33F32* knockout strain of *S. frugiperda* fed on the BXDs-containing maize line ZD958. Similarly, more than 80% of the larvae of the *SeUGT33F24* knockout strain of *S. exigua* died after 7 d of feeding on the ZD958 maize line. It is thus reasonable to predict that knocking out the orthologous gene of *SfUGT33F32* in *S. litura* and *S. littoralis* would also strongly influence their survival on BXDs-containing maize. Therefore, *SfUGT33F32* and its orthologous genes could be an effective molecular target for the development of interventions against *Spodoptera* pests on maize. These interventions might include the use of chemical inhibitors of UGTs or direct targeting of *SfUGT33F32* and its orthologous genes using gene drives (50) or plant‐mediated RNA interference (51).

In conclusion, we demonstrate the key function of the two largest UGT families in determining host range in the *Spodoptera* genus. We uncover a conserved UGT-mediated mechanism of detoxification of DIMBOA in generalist *Spodoptera* species, and reveal a profound genomic signature associated with the transition from generalism to specialism in this genus in the form of repeated UGT gene pseudogenization in the specialist *S. picta*. These findings advance our understanding of the function of the UGT family of enzymes in insects and provide fundamental insights into the evolutionary processes that underlie the genotypic and phenotypic changes arising from interactions between plants and herbivorous insects.

## Materials and Methods

### Insects and Plants.

The wild-type strain XZ of *S. frugiperda* was collected from Xinzheng city, Henan Province, China in 2019. This strain has been maintained in the laboratory for more than 30 generations under artificial diet and without exposure to any insecticides. The XZ-d33c1, XZ-d33c2, XZ-d40c, and XZ-33F32 strains were created from XZ using CRISPR-Cas9 gene editing. The XZ-d33c1 strain is a deficiency strain for a cluster of 10 UGT33 family genes (specifically *SfUGT33F44*, *SfUGT33AT2*, *SfUGT33V12*, *SfUGT33V8*, *SfUGT33F40*, *SfUGT33F39*, *SfUGT33F37*, *SfUGT33F33*, *SfUGT33F32,* and *SfUGT33S3*). The XZ-d33c2 strain is a deficiency strain for a cluster of 4 UGT33 family genes (specifically *SfUGT33B26*, *SfUGT33B29*, *SfUGT33B24,* and *SfUGT33J15*). The XZ-d40c strain is a deficiency strain for a cluster of 15 UGT40 family genes (specifically *SfUGT40F33*, *SfUGT40F34*, *SfUGT40F21*, *SfUGT40D17*, *SfUGT40D16*, *SfUGT40R18*, *SfUGT40R17*, *SfUGT40R14*, *SfUGT40F20*, *SfUGT40F19*, *SfUGT40M13*, *SfUGT40M12*, *SfUGT40M11*, *SfUGT40M10,* and *SfUGT40L10*). The XZ-33F32 strain is homozygous for a 2-bp deletion in exon 1 of *SfUGT33F32*, which would be predicted to produce a truncated and a nonfunctional protein.

The susceptible strain WH-S of *S. exigua* was kindly provided by Yayun Zuo of Northwest Agriculture & Forestry University, and has been maintained in the laboratory without exposure to insecticides for more than 20 y. WH-S-33F24 is homozygous for a 4-bp deletion in exon 1 of *SeUGT33F24*, created from WH-S with CRISPR-Cas9 gene editing technique.

The KY strain of *S. litura* was provided by Keyun Biotechnology Co., LTD. The LS strain of *S. picta* was collected from Lingshan County of Guangxi Province in China.

All the *Spodoptera* strains were reared at 26 ± 1 °C, 60 ± 10% relative humidity and a photoperiod of 16 h light: 8 h dark. All *Spodoptera* larvae except *S. picta* were fed on an artificial diet constituted of corn and soybean powder. *S. picta* larvae were fed on crinum plants. Adults were supplied with 10% honey solution.

The CRISPR mutants are maintained in the Insect Facility at Henan University and are freely available on request. Three maize lines were used in this study, Zhengdan958 (ZD958, Henan Qiule Seeds Technology, Co, Ltd.) is a popular commercial maize line in China, B73 is an inbred maize line used for research, *bx1* is a BXD-deficient mutant line based on the genetic background of line B73. B73 and *bx1* lines were kindly provided by Prof. Xi Zhang of Henan University. Wheat (Jimai22, Crop Research institute, Shandong Academy of Agricultural Science), Rice (Huanghuazhan, Doneed seed, Co, Ltd.), Soybean (Zhonghuang39, Junhao seed, Co, Ltd.), Peanut (Luhua11, Gufeng seed, Co, Ltd.) and Chinese cabbage (Jinzhi30, Tianjin gengyun seed, Co, Ltd.) are popular commercial lines in China. Cotton (Xinhai15) was kindly provided by Ying Chang of Henan University. All of the plants were grown at 26 ± 1 °C with a 16 L:8 D photoperiod.

### CRISPR-Cas9 Knockout of UGT Clusters or Single UGT Gene.

Previously described methods were used for the design and synthesis of sgRNAs (20). Briefly, according to the target sequence principle of 5′-N20NGG-3′ (the PAM sequence is underlined), all sgRNAs were designed using CRISPRdirect (http://crispr.dbcls.jp/). Oligonucleotide primers used to make sgRNA templates are shown in *SI Appendix*, Table S1. sgRNAs was synthesized according to the manufacturer’s instructions (GeneArtTM Precision gRNA Synthesis Kit, Thermo Fisher Scientific, Pittsburgh, PA).

Fresh *S. frugiperda* or *S. exigua* eggs (laid within 1 h) were collected from gauze using 1% (v/v) sodium hypochlorite solution and rinsed with distilled water three times. Eggs were lined up on a microscope slide on double-sided adhesive tape. An approximately one nanoliter mix of sgRNA and Cas9 protein (GeneArt™ Platinum™ Cas9 Nuclease, Thermo Fisher Scientific, Shanghai, China) was injected into individual eggs using a FemtoJet and InjectMan NI 2 microinjection system (Eppendorf, Hamburg, Germany). Injected eggs were placed at 26 ± 1 °C, 60 ± 10% relative humidity for hatching.

Dual sgRNA-directed CRISPR-Cas9 was used to delete the UGT cluster using methods based on our previous work (52). According to the UGT gene positions and orientations on chromosomes, the sgRNA-33F44 and sgRNA-33S3 were designed to target *SfUGT33F44* and *SfUGT33S3* at each end of the first cluster of UGT33 genes (33c1) respectively (Fig. 3*A*). Similarly, sgRNA-33B26 and sgRNA-33J15 were used for the second cluster of UGT33 genes (33c2), and sgRNA-40F33 and sgRNA-L10 for the UGT40 cluster (40c) (Fig. 3 *B* and *C*). Two sgRNA (200 ng/μL for each sgRNA) for each respective UGT cluster and Cas9 protein (200 ng/μL) were mixed and injected into fresh eggs of the XZ strain of *S. frugiperda*. Hatched larvae after injection were reared on artificial diet to pupae and then to adult (G_0_). About twenty single pair adults randomly selected from G_0_ were allowed to produce fertile offspring (G_1_). Then ten second instar larvae from each of the G_1_ single pair families were pooled to prepare genomic DNA and PCR was amplified with the specific primer pairs for UGT gene cluster knockout. The forward primer F44F (located in *SfUGT33F44*) and the reverse primer S3R (located in *SfUGT33S3*) were designed to detect the 33c1 deletion (*SI Appendix*, Fig. S9*A*). A small fragment of genomic DNA (~350 bp) was expected to be amplified with the primer pair F44F/S3R if the 33c1 was deleted. Similarly, primer pairs B26F/J15R and F33F/L10R were used to amplify ~470 bp after the 33c2 deletion and ~860 bp after the 40c deletion respectively. Then other larvae from the single pair family detected as positive for the deletions of interest were reared to adult (G_1_). Individual G_1_ adults were genotyped using the above methods, then moths with the mutations of interest were chosen to mate and produce G_2_ offspring. When the G_2_ were reared to adults, a detection method was developed to distinguish homozygous mutant, heterozygote, and wild-type individuals (*SI Appendix*, Fig. S9). Insect samples generating a PCR band with F44F/S3R but no band with AT2f/AT2r, F32f/ F32r and S3f/S3r are identified as homozygous for the 33c1 knockout (*SI Appendix*, Fig. S9*C*). Likewise, insect samples generating a PCR band with B26F/J15R but no band with B26f/B26r, B29f/B29r, and B24f/B24r are identified as homozygous for the 33c2 deletion (*SI Appendix*, Fig. S9*D*). Finally, insect samples generating a band with F33F/L10R but no PCR band with F33f/F33r, D16f/D16r, and M10f/M10r are identified as homozygous for the 40c knockout (*SI Appendix*, Fig. S9*E*). Homozygous mutant individuals were then mass crossed to generate homozygous knockout strains named as XZ-d33c1, XZ-d33c2, and XZ-d40c strains respectively (Fig. 3). The viability of three UGT cluster knockout strains under our laboratory rearing conditions indicated that none of the three UGT gene clusters is essential for *S. frugiperda* survival.

The single sgRNA-directed CRISPR-Cas9 system was used to knock out *SfUGT33F32* in *S. frugiperda* and *SeUGT33F24* in *S. exigua*. sgRNA (200 ng/μL) and Cas9 protein (200 ng/μL) were mixed and injected into fresh eggs of the XZ strain of *S. frugiperda* for *SfUGT33F32* knockout or the WH-S strain of *S. exigua* for *SeUGT33F24* knockout. Hatched larvae after injection were reared to adult (G_0_). A mass cross with G_0_ moths was executed to obtain G_1_ and then reared to adults. Two specific pairs of primers XZ-33F32F/R and WH-S-33F24F/R were designed to amplify ~350 bp and ~600 bp fragments flanking the sgRNA-target site of *SfUGT33F32* and *SeUGT33F24* respectively (*SI Appendix*, Table S1). For genotyping the G_1_ individuals, the legs of G_1_ moths before mating were used to extract DNA and direct sequencing of the PCR products flanking the sgRNA-target site were used to determine the gene editing type. A total of 14 of the 115 G_1_ adults of *S. frugiperda* genotyped were heterozygous for a 2-bp deletion at exon 1 of *SfUGT33F32* and 17 of the 44 G_1_ adults of *S. exigua* genotyped were heterozygous for a 4-bp deletion at exon 1 of *SeUGT33F24*. These individuals were then mass crossed to produce G_2_. A total of 13 individuals from 76 *S. frugiperda* G_2_ genotyped and 29 individuals from 128 *S. exigua* G_2_ genotyped were identified as homozygous mutant for *SfUGT33F32* and *SeUGT33F24* respectively (Fig. 5 *A* and *B* and *SI Appendix*, Fig. S12). The homozygous mutants were mass crossed to generate homozygous knockout strains named as XZ-33F32 and WH-S-33F24 respectively.

### *Spodoptera* Performance Assay on Plants.

Newly hatched neonates (24 h old or less) were chosen for performance assays. Plant leaves of different growth stage following first sprouting were used for larval feeding as follows: Wheat = 1 wk, maize = 2 wk, rice = 3 wk, Chinese cabbage = 4 wk, cotton, soybean, and peanut were used at the five to seven leaf stage. Forty-eight or sixty larvae of each strain were tested for each plant. The weights of larvae were recorded after about 7 d when the XZ strain larvae on artificial diet had developed to late forth-instar. To evaluate the performance of different strains on different plants, we used the RWI as the assessment criteria. RWI = [the weight of knockout strain on plant/average weight of knockout strain on artificial diet]/[average weight of XZ strain on plant/average weight of XZ strain on artificial diet]. The description could be simplified as the formula RWI = [W^KO^_Plant_/(W^KO^_AD_)_Avg_]/[(W^XZ^_Plant_)_Avg_/(W^XZ^_AD_)_Avg_]. The RWI of background strain XZ on each plant was set as 1, the RWI associated with decreased performance will be less than 1, and the RWI associated with increased performance will be greater than 1.

The effects of *SfUGT33F32* or 33c1 knockout on the survival and development of *S. frugiperda* on maize were analyzed by comparing biological parameters of knockout strains (XZ-33F32, XZ-d33c1) and background strain XZ, which include mortality after 7 d, larval duration, pupation percentage, pupal weight, pupal duration, emergence percentage, female fecundity, and egg hatchability. The ZD958, B73, and *bx1* lines were used for comparing mortality after 7 d of three *S. frugiperda* strains, ZD958 only was used for evaluating other biological parameters of XZ-33F32 and XZ strains. The treatments on artificial diet were used as a control to evaluate the intrinsic effects of *SfUGT33F32* knockout. Each treatment was replicated five times with nine larvae per replicate. Differences of biological parameters between knockout and background strains were evaluated by Student’s *t* test.

The knockout effects of *SeUGT33F24* of *S. exigua* on maize were executed by comparing mortality of knockout strain WH-S-33F24 and background strain WH-S on ZD958 line after 7 d. The mortality of the XZ strain of *S. frugiperda* and the LS strain of *S. picta* on maize ZD958 line and *Crinum asiaticum* were also analyzed after 7 d.

### Bioassays of Phytochemical toxins.

All bioassays of *Spodoptera* were done at 26 ± 1 °C, 60 ± 10% relative humidity and a photoperiod of 16 h light: 8 h dark. Toxicity of phytochemicals was determined with diet incorporation bioassays revised from the method from Wang et al. (52). Briefly, unfed neonates of *S. frugiperda* (within 24 h of hatching) were put on artificial diet mixed with two concentrations of phytochemicals and untreated artificial diet as a control. A total of 24 larvae were used as a biologically independent sample and 3 to 6 replicates of each strain were tested for each concentration. Mortality was recorded after 7 d except for DIMBOA where mortality was recorded after 5 d. Larvae were scored as dead if they died or did not reach the third instar by the end of bioassays. Corrected mortality = (Mortality in response to treatment − Mortality in response to control)/(1 − Mortality in response to control). Differences in corrected mortality at each concentration between knockout and background strains were evaluated by Student’s *t* test at *P* < 0.05.

To estimate the LC_50_ of different strains of *Spodoptera* to DIMBOA, six to twelve concentrations of DIMBOA were used to establish log-probit lines. Diet incorporation bioassays in all *Spodoptera* except *S. picta* were executed as described above. Semiartificial diet (artificial diet: *C. asiaticum* leaf = 1:1 in weight, then mixed thoroughly) was used to do bioassay for *S. picta*. The LC_50_ values (the concentration killing 50% of larvae) and the 95% fiducial limits of the LC_50_ for each strain were calculated through probit analysis of the mortality data using the PoloPlus program (53). Two LC_50_ values were considered significantly different if their 95% fiducial limits did not overlap (54).

## Supplementary Material

Appendix 01 (PDF)

Dataset S01 (XLSX)

Dataset S02 (XLSX)

Dataset S03 (XLSX)

Dataset S04 (XLSX)

Dataset S05 (XLSX)

Dataset S06 (XLSX)

## Data Availability

The raw sequencing data for *S. picta* has been deposited at National Center for Biotechnology Information under the BioProject PRJNA1073306 (55). The genome assembly and annotation of *S. picta* and the transcriptomic raw data for different tissues of *S. frugiperda* have been deposited at DRYAD (56). All study data are included in the article and/or supporting information.
