# Supplementary material for: UDP-glycosyltransferases act as key determinants of host plant range in generalist and specialist Spodoptera species
Source: Proc Natl Acad Sci U S A. 2024 Apr 29;121(19):e2402045121. doi: 10.1073/pnas.2402045121 (PMC11087754; doi:10.1073/pnas.2402045121)
Supplement: Supplementary file 1 — Appendix 01 (PDF) [file pnas.2402045121.sapp.pdf]

Supporting information for

UDP-glycosyltransferases act as key determinants of host plant range in generalist and specialist *Spodoptera* species

Huidong Wang[a,1,*], Jing Song[a,1], Benjamin J. Hunt[b], Kairan Zuo[a], Huiru Zhou[a], Angela Hayward[b], Bingbing Li[a], Yajuan Xiao[a], Xing Geng[a], Chris Bass[b,*], Shutang Zhou[a,*]

[a] State Key Laboratory of Cotton Bio-breeding and Integrated Utilization, School of Life Sciences, College of Agriculture, Henan University, Kaifeng 475004, Henan, China

[b] Centre for Ecology and Conservation, University of Exeter, Penryn, TR10 9FE, UK.

[1] Authors contributed equally

*Correspondence to:

Shutang Zhou, email: szhou@henu.edu.cn

Chris Bass, email: c.bass@exeter.ac.uk

Huidong Wang, email: wanghd@henu.edu.cn

Supplementary results

Computational protein modelling of *Spodoptera* UGTs and ligand transport analysis

The finding that the UGT33 protein SeUGT33F24 can metabolize DIMBOA but SeUGT33F7 cannot offered an opportunity to identify candidate determinants of metabolism at the amino acid level. Indeed, the distance matrix of nine UGT33F family sequences indicates that SeUGT33F7 has a greater sequence identity to the UGT33F that show activity towards DIMBOA, despite it being unable to metabolize the compound (Table S5). To investigate this phenomenon more fully, modeling of protein structure and in silico studies were conducted. CAVER (1) predicated four access tunnels in SeUGT33F24, but only two in SeUGT33F7 (Fig. S19A, Table S6, S7). The $E_{surface}$, E_a, E_{max} and E_{bound} values for SeUGT33F24 tunnel 1 (most probable tunnel for DIMBOA), support the electrostatic steering of DIMBOA towards the surface at the entrance to the tunnel, and the subsequent movement of the compound to the catalytic site within the protein, where it would bind with an affinity of -6.6 kcal/mol (Table S8). In contrast, in tunnel 1 of SeUGT33F7, the E_{bound} value (-1.3 kcal/mol) is higher than the $E_{surface}$ and the E_{max} values (-6.4 and -5.8 kcal/mol respectively) (Table S9). This indicates that DIMBOA would bind with greater affinity at the entrance to the tunnel and is therefore unlikely to be transported to the catalytic site. It also seems unlikely that DIMBOA is bound at the active site of the second access tunnel in SeUGT33F7, as the binding energies here are positive (E_{bound} 9.0 kcal/mol), indicating an energy cost to binding (Table S9). Thus, it appears that SeUGT33F24 is capable of transporting DIMBOA from the surface to the active part of the enzyme, but SeUGT33F7 is not. The SDPlight algorithm (2) was then used to investigate candidate amino acid residues that might be key to the function of the UGT33F subfamily metabolizing DIMBOA. This identified 79 residues that could be implicated in determining specificity towards DIMBOA (Table S10). The access tunnels with the best ligand transport results and therefore most likely to transport DIMBOA were compared to the top 20 residues identified using SDPlight. None of the residues highlighted by SDPlight appeared in SeUGT33F24 access tunnel 1. In contrast, two residues highlighted by SDPlight made up access tunnel 1 in SeUGT33F7, namely SER_{30} (Z-score 1.39; substituted for ASN in SeUGT33F24) and PHE_{191} (Z-score 1.36; substituted for LEU in SeUGT33F24). However, neither of these substitutions caused a change in the tertiary structure of the enzymes (Fig. S17). The largest structural change between the two AlphaFold predictions occurs in α-helix 3 (Fig. S17). There are four amino acid substitutions in this region that are specific to SeUGT33F7 and are not found in the other eight UGT33F sequences in the study (Fig. S18). They are (SeUGT33F7: SeUGT33F24): $ASN_{75}ASP$, $ASN_{82}ASP$, $ASN_{92}THR$ and $GLU_{94}GLY$. These substitutions have created a coiled region at residues 90-92 towards the end of α-helix 3 (Fig. S17). This structural change, along with a further substitution, $ARG_{85}LYS$, alters the shape of the enzyme at the cleft formed where the N- and C-terminals meet, which accommodates the

substrate binding sites. This $ARG_{85}LYS$ substitution is not unique to SeUGT33F7, as it is also found in *S. frugiperda* SfUGT33F32 which can metabolize DIMBOA. However, the four SeUGT33F7-specific amino acid substitutions have altered the structure of α-helix 3, and this change allows the side chains of ARG_{85} to orientate in a different direction to those of LYS_{84} in SeUGT33F24 (Fig. S19).

Supplementary materials and methods

Chemicals

DIMBOA (98%) was purchased from Wuhan Jonk Biological technology Co., Ltd, Hubei, China. MBOA (97%) and UDP-glucose (98%) were purchased from Sigma-aldrich. Gramine (98%) was purchased from J&K Scientific Co., Ltd, Beijing, China. Catechin (98%) was purchased from Bide Pharmaceutical Technology Co., Ltd, Shanghai, China. Gossypol acetate (98%) was purchased from Shanghai Yuanye Bio-Technology Co., Ltd, China. Ferulic Acid (99%) and tannic acid (95%) were purchased from Aladdin Biochemical Technology Co., Ltd, Shanghai, China. 1-NA (99.8%) was purchased from MedChemExpress. DIMBOA-Glc (70%) was kindly provided by Prof. Xi Zhang of Henan University.

Heterologous expression and quantification of UGT proteins

All recombinant UGTs were expressed using the Bac-to-Bac Baculovirus Expression System (Invitrogen). Briefly, the open reading frame of UGT33 genes of *Spodoptera* were PCR amplified from the XZ strain for *S. frugiperda*, WH-S strain for *S. exigua*, KY strain for *S. litura*, and then cloned into the pFastBacHTC vector (Invitrogen) with In-Fusion Snap Assembly cloning kits (Takara Bio, China). Primers for full length cloning are shown in Table S1. The open reading frame of *SfUGT33AT2*, *SeUGT33F7, SlittUGT33F55*, *SlittUGT33F4* and *SpUGT33F34* were synthesized directly (sequences in Dataset S4). Plasmids of recombinants were transfected to *E. coli* DH10Bac competent cells, and the recombinant bacmids were then transfected to Sf9 cells using the FuGENE® HD Transfection Reagent (Promega, Madison, Wisconsin). High titer recombinant baculovirus stocks were infected to High Five cells for producing recombinant UGT proteins.

The content of recombinant UGTs in microsomal protein were evaluated using the Microsome Isolation Kit (Abcam) and quantified use a Jess Simple Western System (Protein Simple, USA) using a Protein Normalization Assay Module kit (Protein Simple). Each microsome sample was diluted (1:100) and loaded into each well. Recombinant UGTs were detected using a 6×His Tag Monoclonal antibody diluted 1:200 (Thermo Fisher Scientific), and an anti-mouse module were used for chemiluminescent detection. Recombinant Human transglutaminase 2 protein (TGM2, Sino Biological Inc., Beijing, China) with a 6×His tag was used as the standard to quantify recombinant UGT proteins.

In vitro metabolism of chemicals

In vitro metabolism by recombinant UGT enzymes was performed with 5 μg crude microsomal UGT protein, 500 μM UDP-glucose, 1 mM $MgCl_2$, and substrate (5 μM 1-NA, 20 μM DIMBOA) in 100 μl 0.1 M potassium phosphate buffer (pH 7.4). Reactions started by adding

substrate after pre-warming the reaction system at 30°C for 5 minutes. Samples were incubated on a shaking incubator for 1 hour at 30 °C, 1200 rpm for 1-NA and DIMBOA. Reactions were stopped by adding 100 μl methanol, then 800 μl dilution buffer (50% ddH_2O and 50% methanol) was added to each sample and incubated for an additional 20 min. The stopped reactions were centrifuged at 18,000×g for 10 min, 200 μl cleaned supernatant was transferred to HPLC vials and analyzed. The reaction mixture with the crude microsomal preparations from non-transfected cells was used as the control.

Spodoptera fifth instar larvae feeding on artificial diet were used to compare the UGT enzymatic activity of midguts between knockout and background strains. Caterpillars were dissected in cold phosphate buffer (Na_2HPO_4 8 mM, NaCl 136 mM, KH_2PO_4 2 mM, KCl 2.6 mM), and their midgut tissues were collected and cleaned from gut contents. Five individuals were pooled and homogenized in the same phosphate buffer as a biological replicate, three biological replicates were performed for each strain. The protein concentrations of midgut homogenate were determined with the BCA protein detection kit (Thermo Scientific). The enzyme activities were performed with 500 μg midgut homogenate, 500 μM UDP-glucose, 1 mM $MgCl_2$, and 20 μM DIMBOA in 100 μl 0.1M potassium phosphate buffer (pH 7.4). The subsequent process was the same as described for metabolism assays by recombinant UGT enzymes described above.

UPLC-MS/MS

The formation of metabolite DIMBOA-Glc and clearance of substrate 1-NA were applied as the criterion of enzyme activity for DIMBOA and 1-NA respectively. Samples were separated by HSS T3 column (2.1×100 mm, 1.8 μm particle size, Waters, MA) on UPLC system (Waters ACQUITY UPLC I-Class) with different gradient elution programs, the flow speed was 0.3 ml/min. 5 μl samples were separated with A (methanol) and B (water/0.1% (v/v) formic acid). The gradient elution program of DIMBOA-Glc was set as follows: 0 min A: B 15:85, 5 min A: B 15:85, 20 min A: B 60:40, 21 min A: B 100:0, 22 min A: B 100:0, 22.5 min A: B 15:85, 25 min A: B 15:85. The linear gradient system of 1-NA was set as follows: 0-2 min, 5% A; 4-7 min, to 95% A; 7-8 min, 5% A; 8-10 min, to 5% A. Samples were detected by tandem quadrupole mass spectrometer (Xevo TQ-XS, Waters) run in negative ESI mode and multiple reaction monitoring (MRM), with the MRM transitions 371.93 > 148.93 and 142.88 > 114.94 used for quantification of DIMBOA-Glc and 1-NA respectively.

mRNA expression profiling of *S. frugiperda* UGTs

Seven tissues (head, epidermis, hemolymph, fat body, foregut, midgut and hindgut) from six instar larvae of the *S. frugiperda* XZ strain were dissected and prepared for transcriptome sequencing. Ten individuals were pooled as a biological replicate and three biological replicates were performed for each tissue. Extraction, quality and quantity analysis of total RNA, library

preparation and sequencing were completed on the DNBSEQ gene sequencing platform (Huada Genomics Co., Ltd, Shenzhen, China). The raw data was filtered with SOAPnuke (v1.4.0) (3). The clean data were mapped to the reference genome by HISAT (v2.1.0) (4) and the assembled unique gene by Bowtie2 (v2.2.5) (5). The expression level of genes was calculated by RSEM (v1.2.8) (6). The reference genome used was the Faw-zju isolate (GenBank GCF_011064685.2) from Zhejiang University and the *SfUGT34A24* and *SfUGT44A24* genes were manually added to the reference genome to compensate for the deficiency of these two genes in the reference genome. The heatmap of tissue-specific transcriptomic profiling were visualized by TBtools (7).

Genome sequencing and assembly of *S. picta*

High molecular weight DNA was provided to Novogene UK Ltd for sequencing and basecalling using the Nanopore PromethION platform and Guppy v6.3.9, generating 71.6 GB of FASTQ sequencing data classified into quality pass/fail libraries. Passing reads were assembled using Flye v2.9.1 (8) with two rounds of polishing. The draft assembly was subject to three further rounds of polishing with Pilon v1.24 (9) using Illumina DNAseq data trimmed with Trim Galore (10) and aligned using the bwa mem algorithm (11), followed by removal of haplotigs using purge_dups v1.2.5 (12). Contaminating contigs were identified and removed using Blobtools v1.1.1 (13) and the final assembly then softmasked for repetitive sequences using Red v2.0 (14). Gene models were predicted using Braker v2.1.6 (15, 16) using RNAseq evidence trimmed with Trim Galore aligned with HISAT2 v2.1.0 (17). The annotation files were filtered to exclude incomplete or short (< 200bp) gene models, and then the longest transcript per gene was functionally annotated using OmicsBox 3.1.2 (18), with BLAST homology searches run against the NCBI NR Arthropoda dataset. Genome completeness was assessed using the Benchmarking Universal Single-Copy Orthologues (BUSCO) pipeline (19).

Bioinformatic and phylogenetic analysis

UGT genes of S. *frugiperda, S. exigua*, *S. litura*, *S. littoralis* and *S. picta* were identified using the BLAST algorithm within the Geneious software suite (Biomatters, New Zealand) and *H. armigera*, *B. mori* and annotated *Spodoptera* UGTs as query sequences. The chromosome-level assemblies were used as follows, S. *frugiperda* (ZJU_Sfru_1.1, Faw-zju isolate, GCA_011064685.2; AGI-APGP_CSIRO_Sfru_2.0, SF20-4 isolate, GCA_023101765.3), *S. exigua* (PGI_SPEXI_v6, GCA_902829305.4), *S. litura* (ASM270686v3, GCA_002706865.3), *S. littoralis* (PGI_Spodlit_v1, GCA_902850265.1) and *S. picta* (this study assembly). All *S. frugiperda* UGT genes were verified by PCR amplification and sanger sequencing. The genomic regions containing *Spodoptera* UGT33 and UGT40 genes located were checked carefully to avoid overestimating gene losses. The Multiple alignment algorithm MUSCLE (20), conserved motif (21) and exon/intron organization were used to verify UGT gene completeness. The

annotated genes were submitted to the UGT Nomenclature Committee (https://labs.wsu.edu/ugt/) for formal naming.

The 47 *S. frugiperda* UGT proteins and outgroup fringe were aligned using the MUSCLE algorithm (20) within the Geneious software suite (Biomatters). MEGA11 (22) was used to determine the best-fit model of amino acid substitution, using a maximum likelihood fit of 56 different models. The substitution model with the lowest Bayesian Information Criterion score was selected for use in phylogeny estimation. The phylogenetic tree was constructed using Bayesian inference (23) [substitution model LG+G (24) ; chain length, 1,100,000; subsampling frequency, 200; burn-in length, 100,000; heated chains, 4; heated chain temperature, 0.2].

For gene gain/loss analysis, all UGT33 and UGT40 genes including partial and pseudogenes were used, but the indel and premature stop codon of pseudogenes were manually removed to obtain corrected amino acid sequences (Dataset S3). A *Spodoptera* species tree with divergence time was derived from (25). Orthofinder -v2.5.4 (26) analysis was performed separately on UGT33 and UGT40 amino acid sequences to define orthologs groups and duplications within these families in five *Spodoptera* species, using the user-specified tree parameter (-s) to use the species tree previously generated. NOTUNG (27) within TBtools (7) was used to reconcile gene gain/loss events of the UGT33 and UG40 family genes, using the species tree previously generated and gene tree estimated by IQ-TREE (28) within TBtools based on maximum likelihood algorithm with 5000 ultrafast bootstraps.

The conserved motifs of *S.frugiperda* UGT proteins were identified using the MEME database (21). The exon/intron organization of *S. frugiperda* UGT genes were visualized by Gene Structure Display Server (GSDS) database (29). The location information and distribution of *S. frugiperda* UGTs on chromosomes were depicted with TBtools (7).

Modeling of protein structure and in silico studies

A three-dimensional model of *SeUGT33F24* and *SeUGT33F7* was generated using the AlphaFold2 structure prediction software (30, 31). The quality and stereochemical soundness of the model was assessed using ERRAT (32) and PROCHECK (33), and Ramachadran plots were generated using the Structural Analysis and Verification Server (https://saves.mbi.ucla.edu/). The prepared models were exported as 'holoenzymes' in a .pdb format and uploaded to Caver Web v1.2 for further analysis (1). The volume, relevance score and druggability score of the catalytic pockets were estimated for each model, before access tunnel detection was conducted. The structure of DIMBOA was obtained from PubChem (compound CID: 2358) and minimised in UCSF Chimera v 1.16 (34) (steepest descent steps: 1000; steepest descent step size: 0.02 Å; conjugate gradient steps: 100; conjugate descent step size: 0.02 Å). Subsequent transport analyses were conducted with DIMBOA for all relevant

access tunnels detected. The activation energy (E_a), highest binding energy (E_{max}), binding energy at the tunnel entrance ($E_{surface}$) and binding energy in the bound state (E_{bound}) values were calculated for all tunnels.

Nine UGT33F subfamily genes including the orthologs and the most closely related paralogous genes of *SfUGT33F32* in *S. frugiperda*, *S. exigua*, *S. litura* and *S. littoralis* were classified into two groups according to the ability of metabolizing DIMBOA to identify specificity-determining positions (SDPs) by SDPlight algorithm (2). Predicted substrate access tunnels were inspected for the inclusion of residues highlighted in the top 20 SDPlight results (based on Z-score).

The holoenzymes were exported to UCSF Chimera v 1.16 (34) and the secondary structure was examined with reference to the structures known to be implicated in binding and function in other UGTs. The catalytic dyad residues, UGT motif, N-terminal and C-terminal domains were identified on the structures. The secondary structures, as predicted by AlphaFold (30, 31), of SeUGT33F24 and SeUGT33F7 were mapped on to a multiple sequence alignment and this was used to identify key regions with structural differences.

S. frugiperda Faw-zju

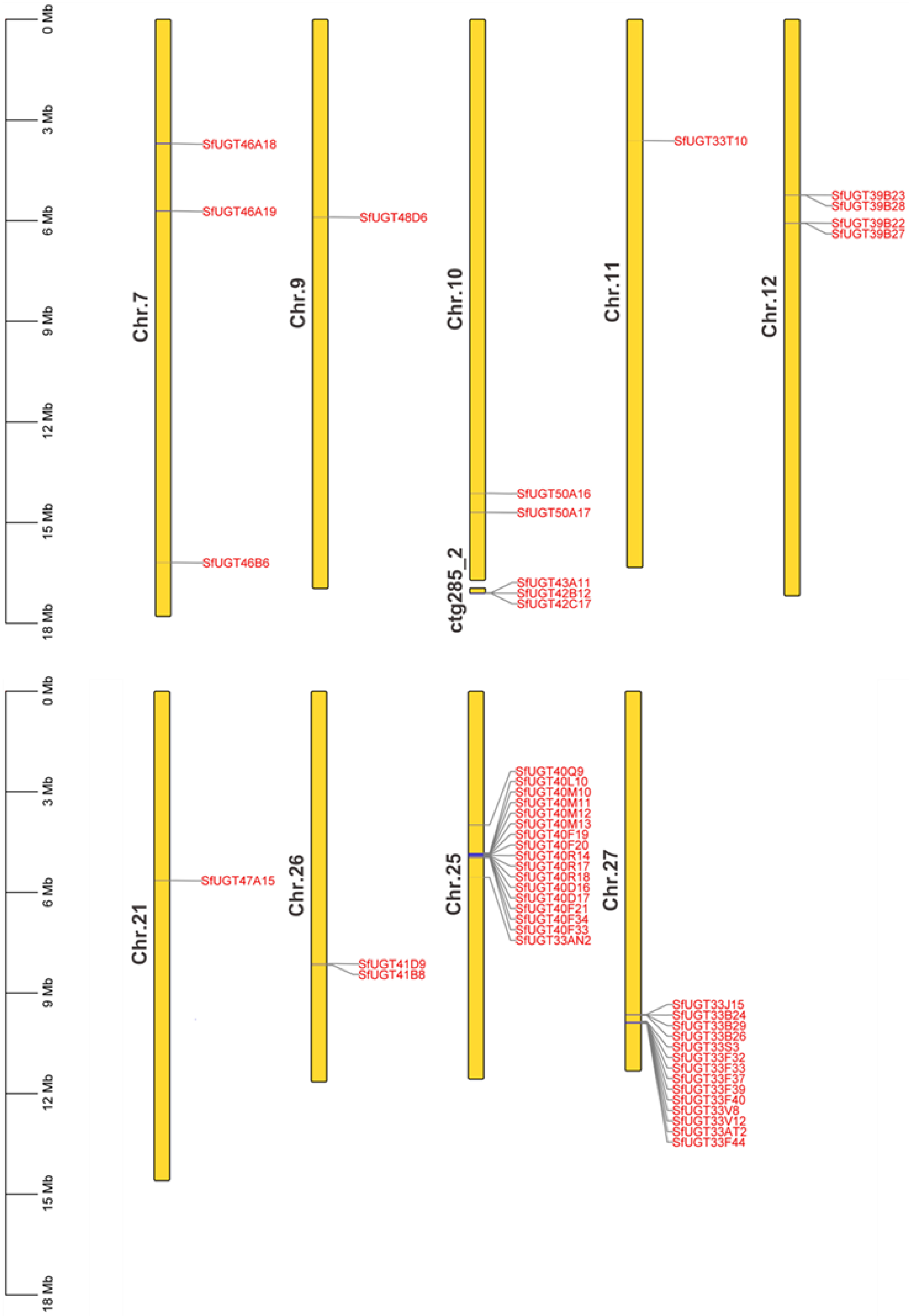

Supplementary Figure 1. Genomic position of the UGT genes of *S. frugiperda*. Gene distribution is shown in *S. frugiperda* genome assembly Faw-zju. Figure was created using the TBtools software (7). The Faw-zju isolate was as the reference genome for the description of chromosomal gene locations in the main text of the manuscript.

S. frugiperda SF20-4

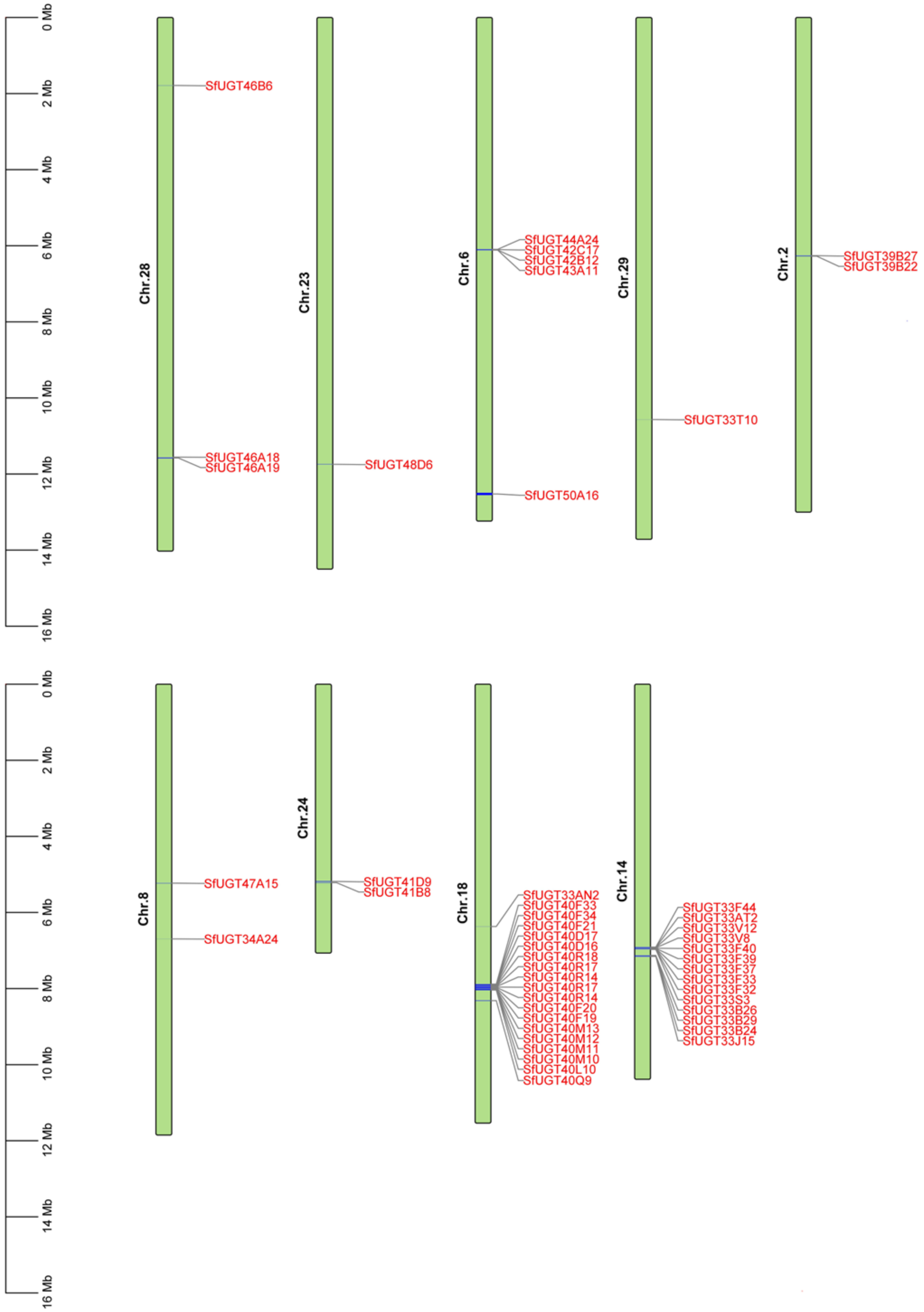

Supplementary Figure 2. Genomic position of the UGT genes of *S. frugiperda*. Gene distribution is shown in *S. frugiperda* genome assembly SF20-4. Figure was created using the TBtools software (7).

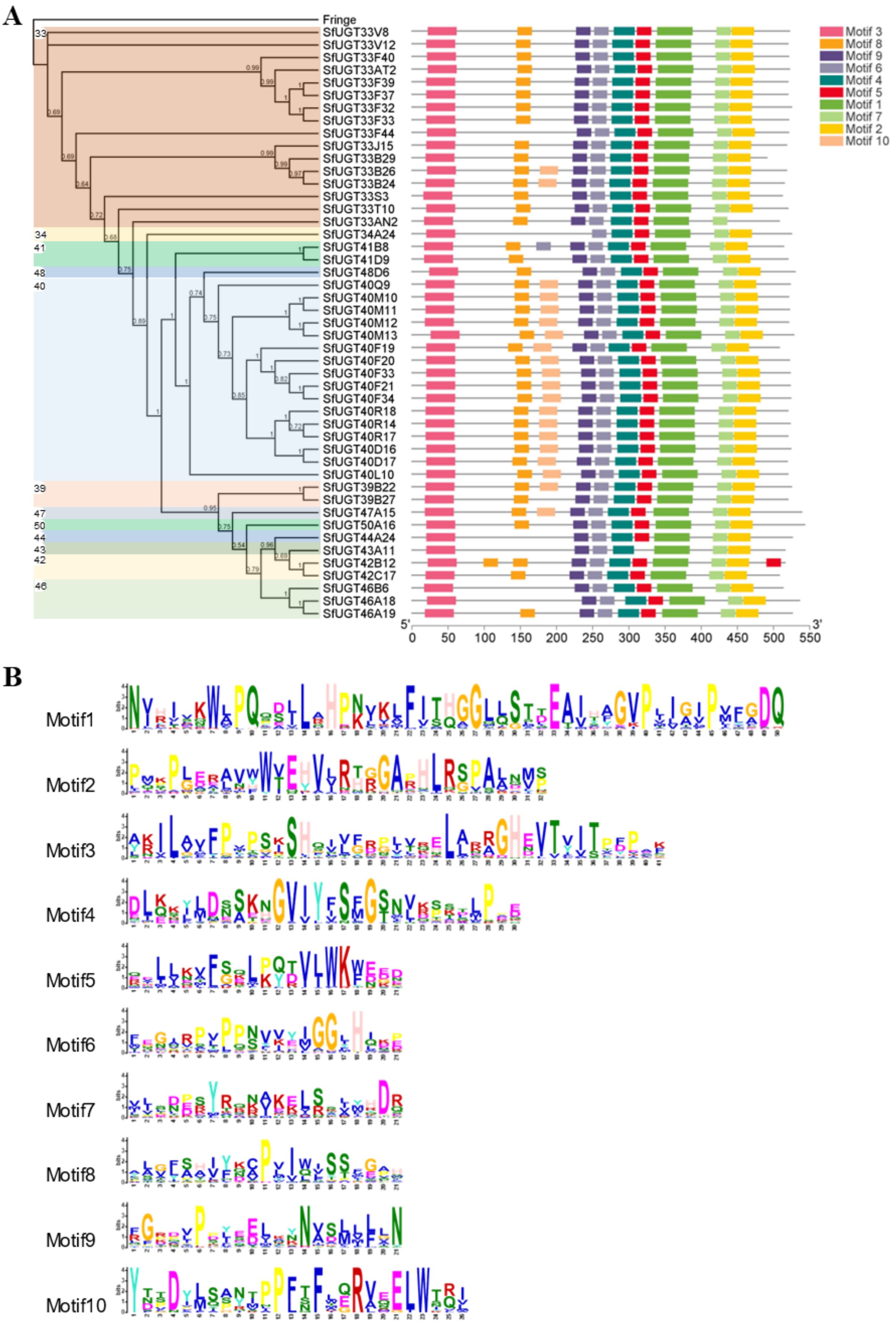
A
Fringe
SfUGT33V8
SfUGT33V12
SfUGT33F40
SfUGT33AT2
SfUGT33F39
SfUGT33F37
SfUGT33F32
SfUGT33F33
SfUGT33F44
SfUGT33J15
SfUGT33B29
SfUGT33B26
SfUGT33B24
SfUGT33S3
SfUGT33T10
SfUGT33AN2
SfUGT34A24
SfUGT41B8
SfUGT41D9
SfUGT48D6
SfUGT40Q9
SfUGT40M10
SfUGT40M11
SfUGT40M12
SfUGT40M13
SfUGT40F19
SfUGT40F20
SfUGT40F33
SfUGT40F21
SfUGT40F34
SfUGT40R18
SfUGT40R14
SfUGT40R17
SfUGT40D16
SfUGT40D17
SfUGT40L10
SfUGT39B22
SfUGT39B27
SfUGT47A15
SfUGT50A16
SfUGT44A24
SfUGT43A11
SfUGT42B12
SfUGT42C17
SfUGT46B6
SfUGT46A18
SfUGT46A19
Motif 3
Motif 8
Motif 9
Motif 6
Motif 4
Motif 5
Motif 1
Motif 7
Motif 2
Motif 10
5'
3'
0 50 100 150 200 250 300 350 400 450 500 550
B
Motif1
Motif2
Motif3
Motif4
Motif5
Motif6
Motif7
Motif8
Motif9
Motif10

Supplementary Figure 3. Conserved motifs in the UGTs of *S. frugiperda* identified using the MEME database (21). (A) The boxes of 10 various colors indicate different conserved motifs. (B) The sequence logo of 10 conserved motif of *S. frugiperda* UGTs. The MEME online database was used to identify 10 Motifs by submitting all *S. frugiperda* UGT amino acid sequences. *SfUGT42B12* has two Motif5s and two Motif8s, *SfUGT41B8* has two Motif6s. The sequences of *SfUGT42B12* and *SfUGT41B8* were confirmed as correct by PCR and Sanger sequencing. Motif5, Motif6 and Motif8 are shorter and contain less conserved amino acids than Motif1-4, which makes it more likely that some UGT genes may contain more than one of these motifs.

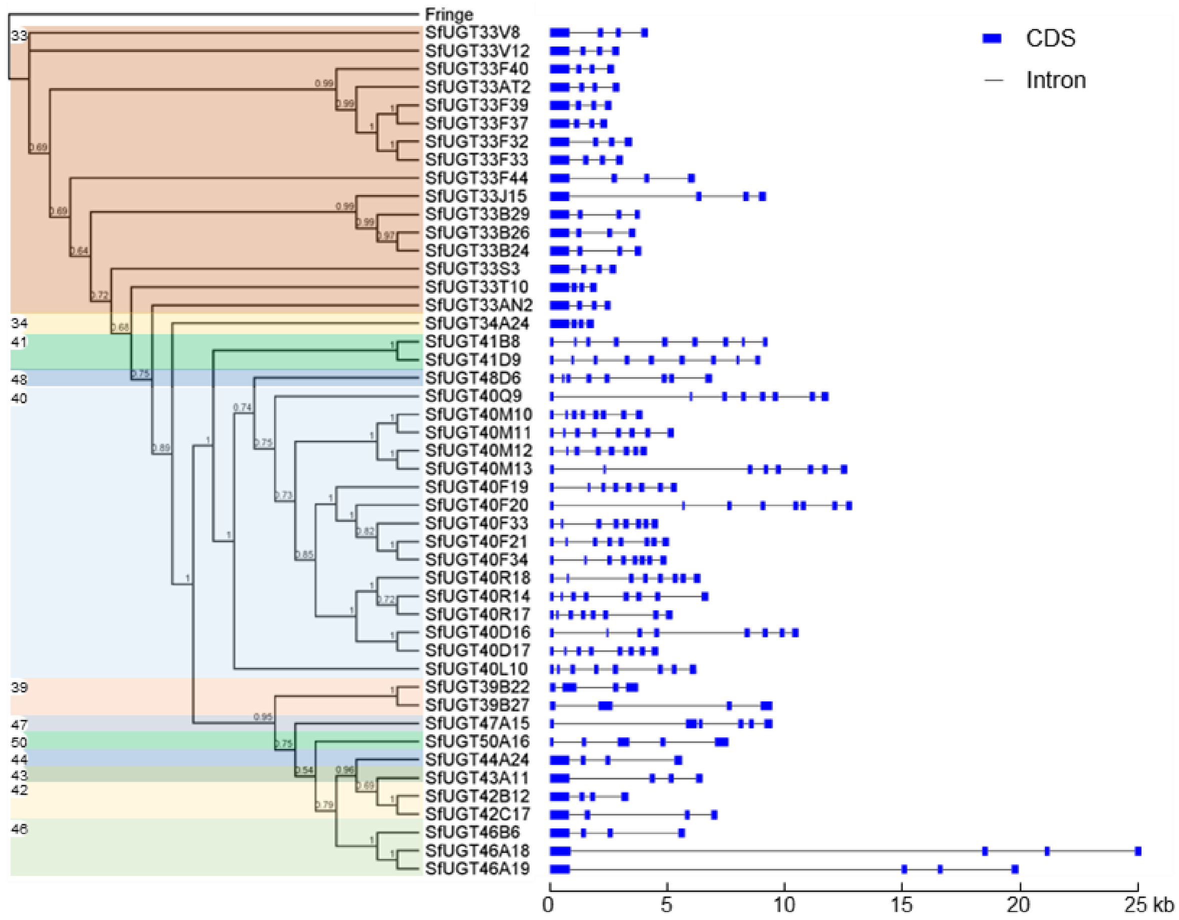

Supplementary Figure 4. The exon-intron structure of 47 UGT genes of *S. frugiperda*.

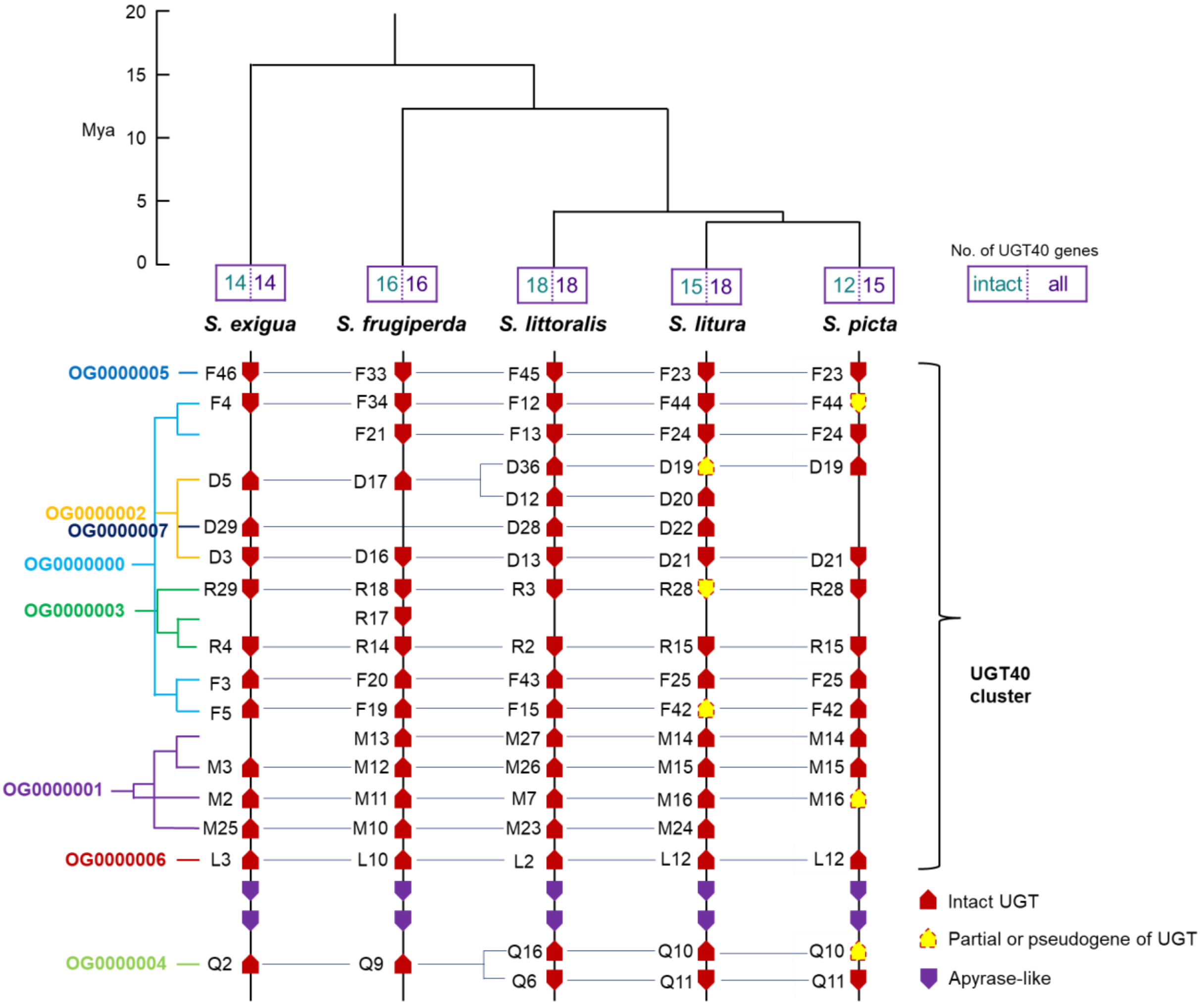

Supplementary Figure 5. Syntenic analysis of UGT40 family genes in five species of *Spodoptera*. The species tree and divergence time (Mya) were derived from (25). UGT genes are shown in their correct orientation and order but physical distances are not to scale. The red arrows indicate UGT genes with intact sequences, the dashed arrow filled in yellow indicate partial or pseudogene of UGTs. OG0000000-0000007 are orthogroups (a set of genes that have descended from a single gene from the last common ancestor in a clade of species) predicted by OrthoFinder (26).

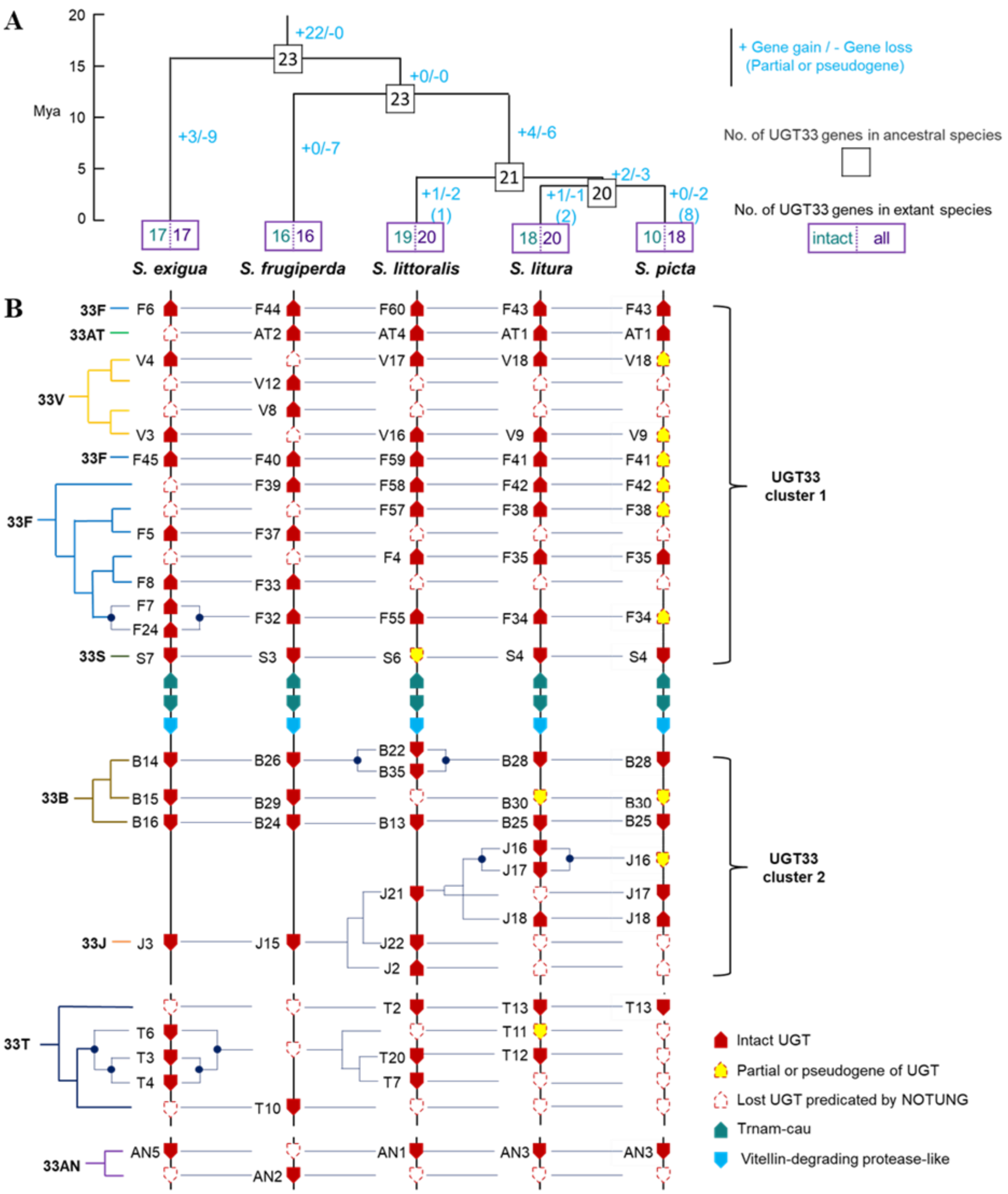

Supplementary Figure 6. Syntenic analysis and estimation of gene gain and loss events of UGT33 family genes in five *Spodoptera* species. (A) Estimation of gene gain and loss events and number of UGT33 genes in ancestral and extant species. The number of gene gains and losses are shown on tree branches and indicated with + and −, respectively, the numbers of partial and pseudogenes are shown in brackets, the numbers of UGT33 genes in ancestral and extant species were in black and purple boxes respectively The species tree and divergence time were derived from (25). (B) Syntenic analysis of UGT33 family genes in five *Spodoptera* species. The UGT genes are shown in their correct orientation and order but physical distances

are not to scale. Gene duplications within species are indicated with ●. Gene gain and loss events were estimated using NOTUNG (27), with data shown in full in Fig. S20. Red arrows indicate UGT genes with intact sequences, dashed arrows filled in yellow indicate partial or pseudogene UGTs, dashed arrows filled in white indicate UGT gene losses predicted by NOTUNG (27).

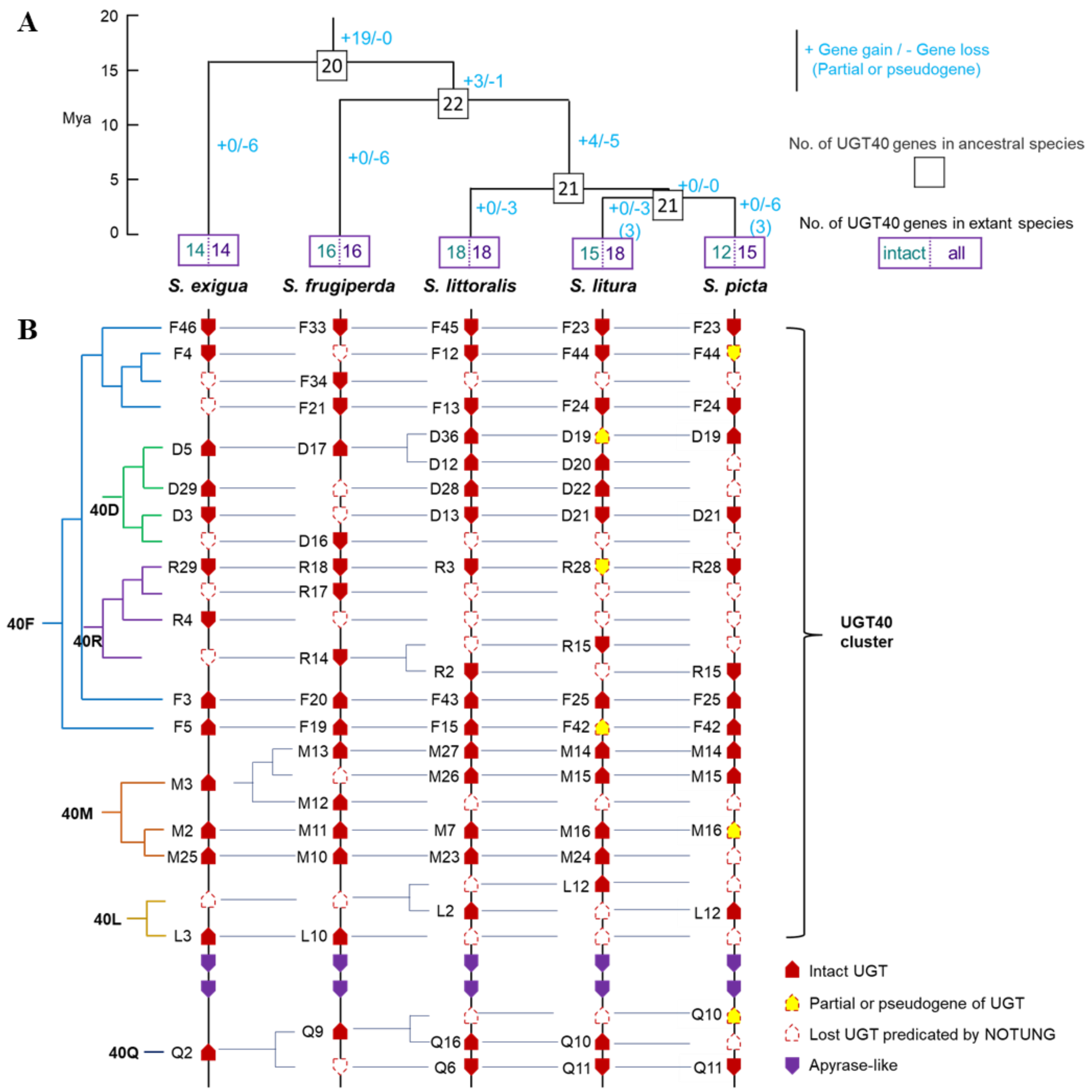

Supplementary Figure 7. Syntenic analysis and estimation of gene gain and loss events of UGT40 family genes in five *Spodoptera* species. (A) Estimation of gene gain and loss events and number of UGT40 genes in ancestral and extant species. The number of gene gains and losses are shown on tree branches and indicated with + and −, respectively, the numbers of partial and pseudogenes are shown in brackets, the numbers of UGT40 genes in ancestral and extant species were in black and purple boxes respectively. The species tree and divergence time were derived from (25). (B) Syntenic analysis of UGT40 family genes in five *Spodoptera* species. The UGT genes are shown in their correct orientation and order but physical distances are not to scale. Gene duplications within species are indicated with ●. Gene gain and loss events were estimated using NOTUNG (27), with data shown in full in Fig. S21. Red arrows indicate UGT genes with intact sequences, dashed arrows filled in yellow indicate

partial or pseudogene UGTs, dashed arrows filled in white indicate UGT gene losses predicted by NOTUNG (27).

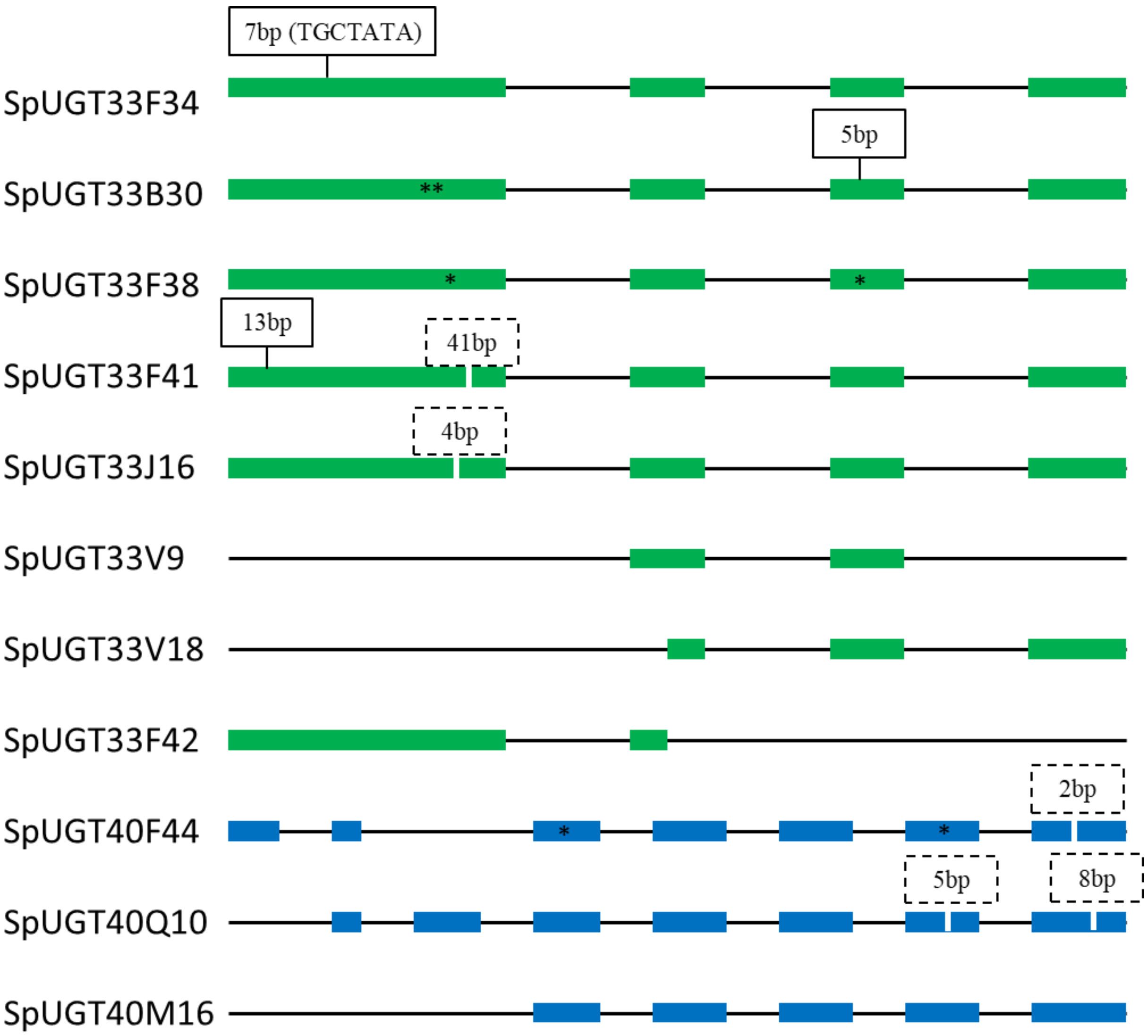

Supplementary Figure 8. **Annotation of nonfunctionalizing mutations in 8 UGT33 and 3 UGT40 genes of *S.picta*.** Green and blue rectangles indicate UGT33 and UGT40 genes respectively, * indicates premature stop codon, solid line and hashed line boxes indicate insertions and deletions respectively.

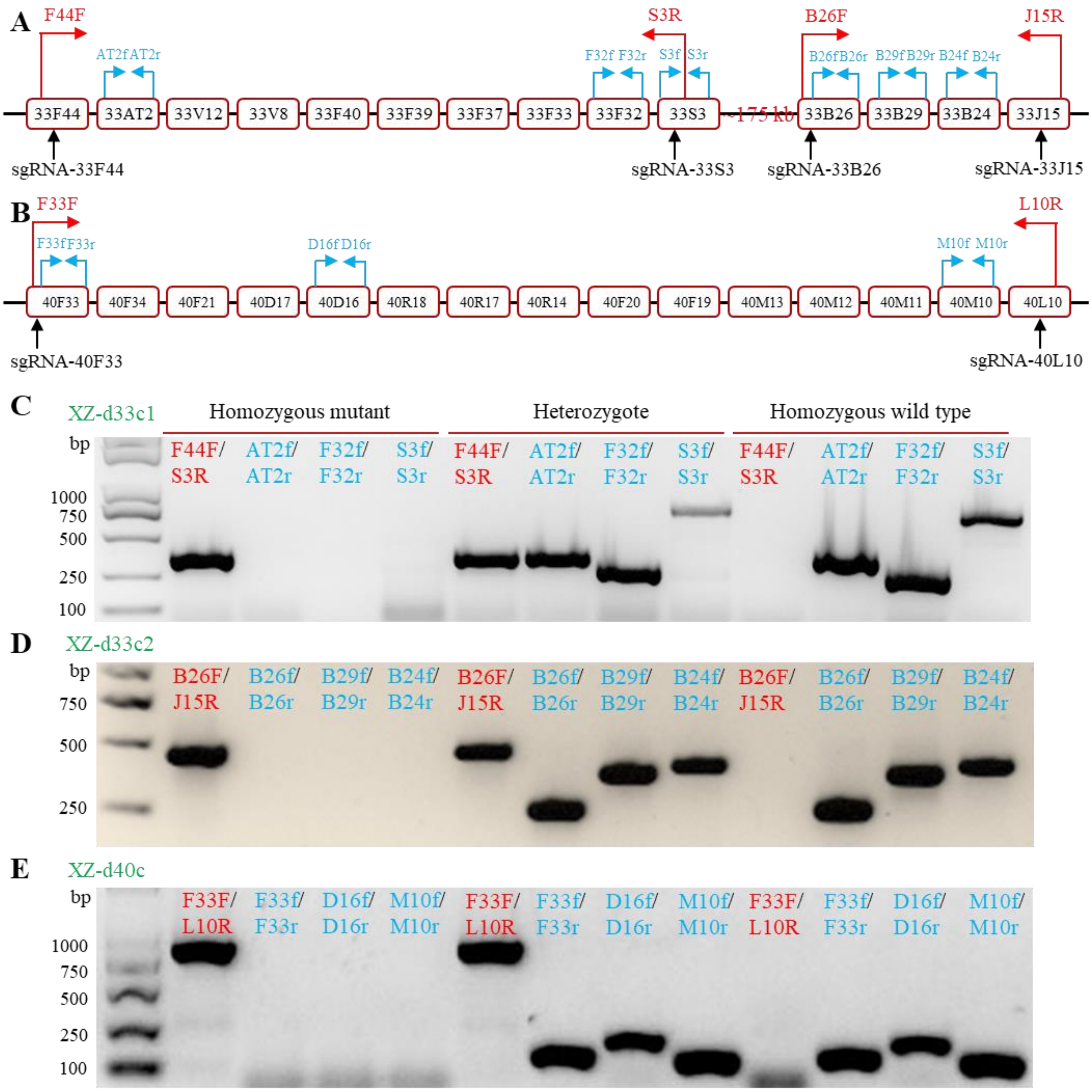

Supplementary Figure 9. CRIPSPR-Cas9-mediated knock out of the UGT33 and UGT40 clusters of *S. frugiperda*. (A, B). Positions of sgRNAs and primer pairs used for allele-specific PCR detection. (C, D, E). Genotyping of individual *S. frugiperda* for deletion of the UGT cluster according to banding patterns of the PCR products amplified with a set of four primer pairs. The PCR bands amplified by primer pairs shown in red spanning two sgRNAs represent deletion events of UGT clusters. The PCR bands amplified by primer pairs shown in blue within the UGT cluster indicate the presence of UGT genes in the cluster. Individuals with PCR bands amplified by the primer pairs shown in red and without a band amplified by the primer pairs shown in blue were considered homozygous mutant for the UGT gene cluster knockout.

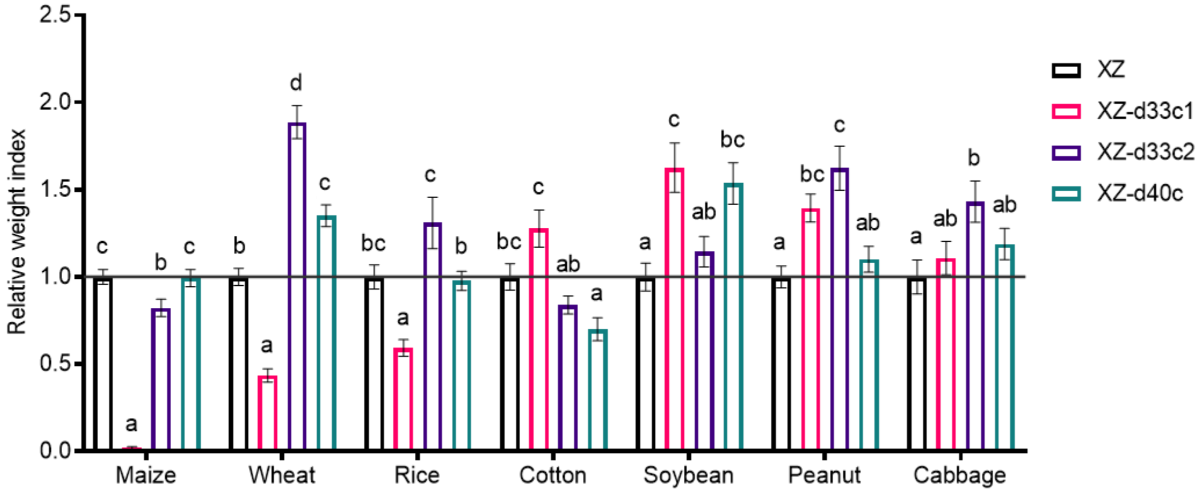

Supplementary Figure 10. Performance of three UGT knockout strains of *S. frugiperda* and the wildtype background strain XZ on seven host plants. Relative weight index (RWI) = (the weight of knockout strain on plant / average weight of knockout strain on artificial diet) / (average weight of XZ strain on plant / average weight of XZ strain on artificial diet). Forty-eight or sixty individuals were tested on each plant or artificial diet. Significant differences ($p < 0.05$) are denoted using letters above bars as determined by one-way ANOVA with Tukey HSD.

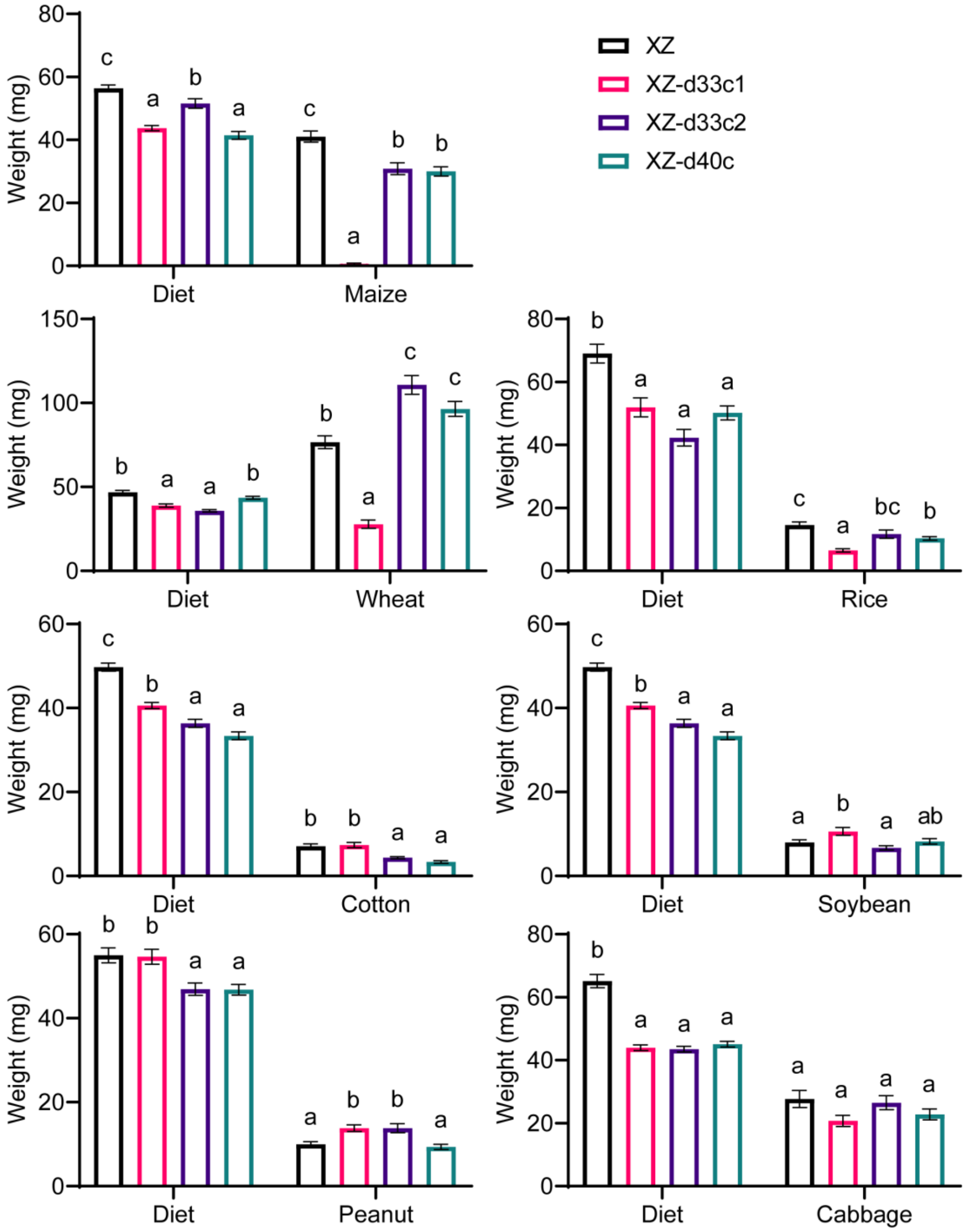

Supplementary Figure 11. Performance of three UGT knockout strains and the wildtype background strain XZ of *S. frugiperda* on seven host plants and artificial diet. Forty-eight or sixty individuals were tested on each plant or artificial diet. Significant differences ($p < 0.05$) are denoted using letters above bars as determined by one-way ANOVA with Tukey HSD.

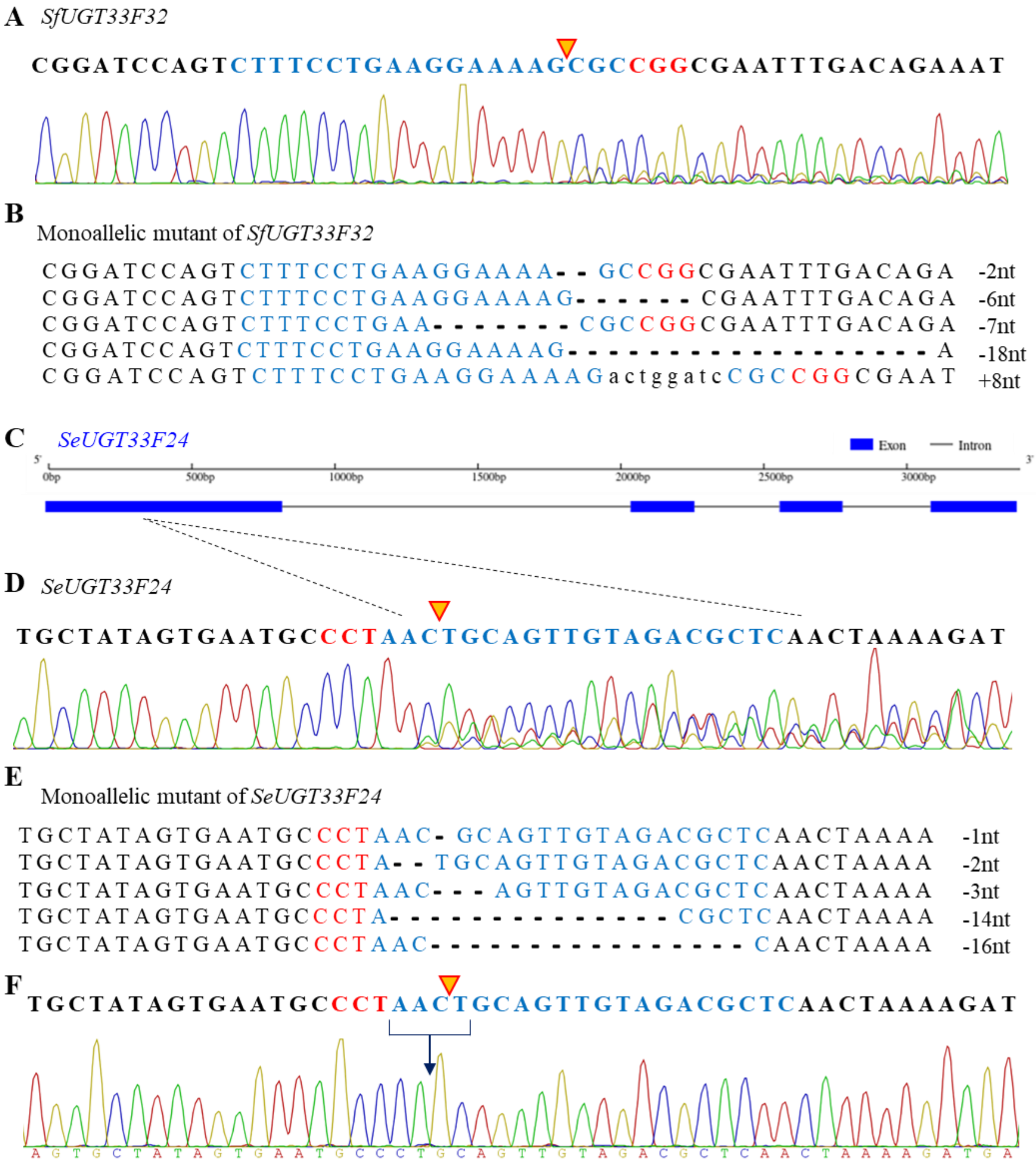

Supplementary Figure 12. CRISPR/Cas9-mediated knockout of *SfUGT33F32* in *S. frugiperda* (A, B) and *SeUGT33F24* in *S. exigua* (C-F). (A) Multiple peaks in chromatograms of directly sequenced PCR products of the G_0 larvae indicate the presence of indel mutations in *SfUGT33F32* (A) and *SeUGT33F24* (D). The target sequences of the wild type *SfUGT33F32* and *SeUGT33F24* allele are highlighted in blue, the PAM sequences are highlighted in red, the cleavage sites are indicated with yellow inverted triangle. (C) Schematic of the sgRNA targeted site and sequences in the exon 1 of *SeUGT33F24.* (B, E) Sequences of indel mutations flanking the target site in G_0 larvae. Deleted bases are represented by dashes and inserted bases are exhibited in lower case. The numbers of bases deleted or inserted are listed at the right side of

sequences. (F) A representative chromatogram derived from direct sequencing of PCR products flanking the sgRNA site of *SeUGT33F24* from the WH-S-33F24 strain.

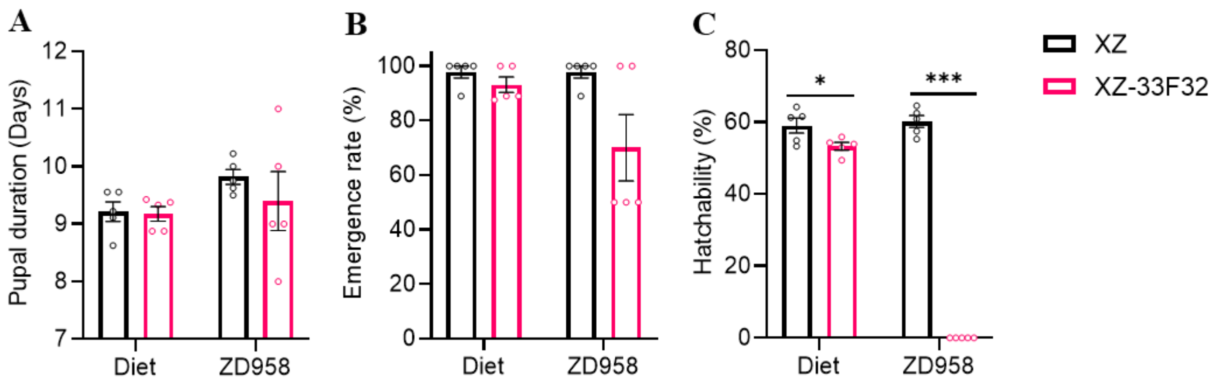

Supplementary Figure 13. **Performance of the *SfUGT33F32* knockout line on artificial diet and maize ZD958.** Metrics assessed were: pupal duration (A), emergence rate (B) and hatchability (C). Error bars represent mean values ± SEM (n = 5), unpaired t test was used for statistical significance comparison, *$p < 0.05$, ***$p < 0.001$.

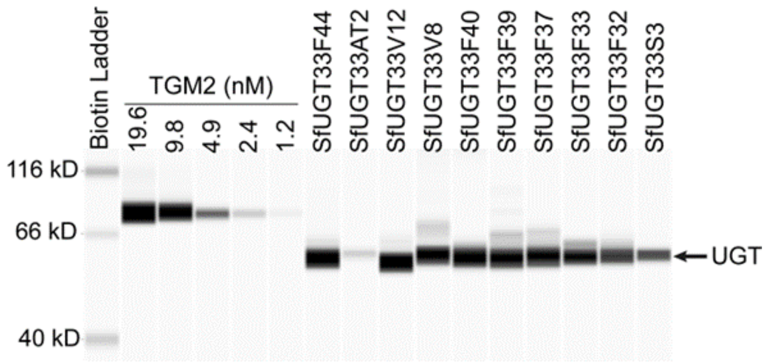

Supplementary Figure 14. Representative western blots showing immunoreactive UGT33 family proteins. Recombinant Transglutaminase 2 (TGM2, Sino Biological Inc.) was used to construct a standard curve through serial dilution in order to quantify UGT protein content.

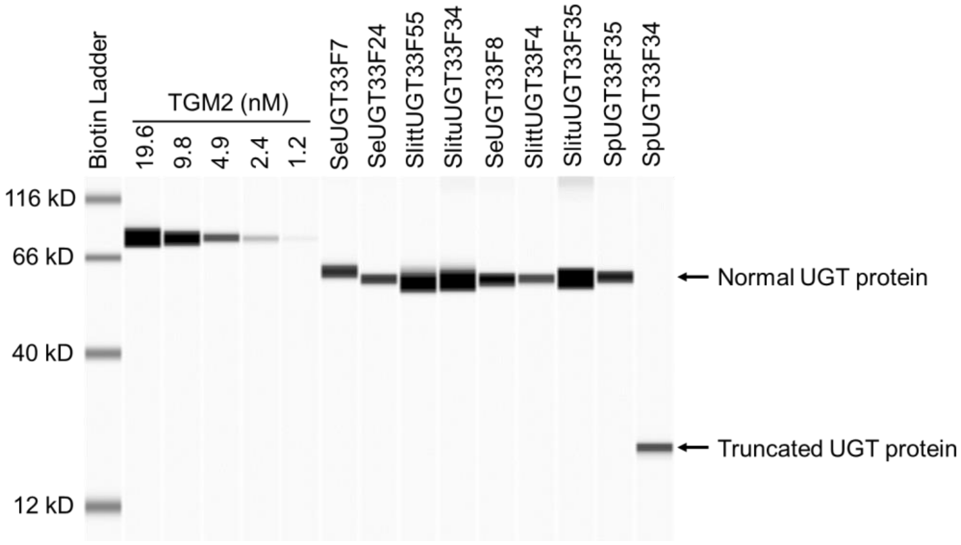

Supplementary Figure 15. Representative western blots showing immunoreactive UGT33 family proteins from four *Spodoptera* species derived from protein simple (Jess) assays. Se, *S. exigua*; Slitu, *S. litura*; Slitt, *S. littoralis*; Sp, *S. picta*. Recombinant Transglutaminase 2 (TGM2, Sino Biological Inc.) was used to construct a standard curve through serial dilution to quantify UGT protein content. SpUGT33F34 was shown as a truncated protein because a 7 bp insertion in exon 1 of this gene leads to premature termination of *SpUGT33F34* translation.

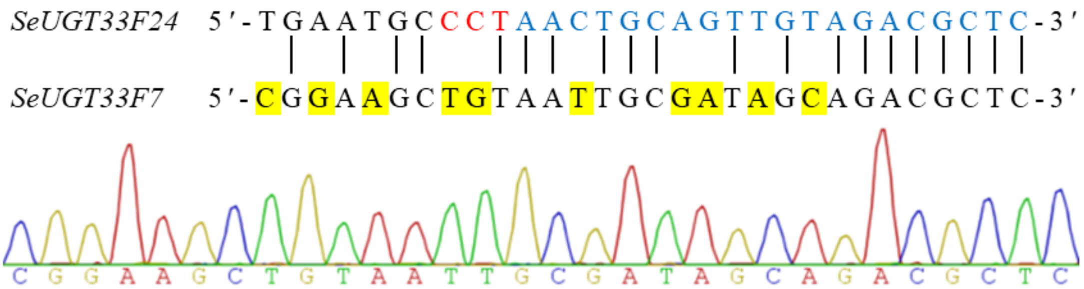

Supplementary Figure 16. **Confirmation that CRISRP/Cas9 gene editing of *SeUGT33F24* did not result in off-target effects on the *SeUGT33F7* gene in the WH-S-33F24 strain.** The sgRNAs are indicated in blue and the PAM sequences in red, the different base pairs between *SeUGT33F24* and *SeUGT33F7* are highlighted. A representative sequencing chromatogram indicates only the wild type sequence present at the potential off-target site of the *SeUGT33F7* gene in the WH-S-33F24 strain.

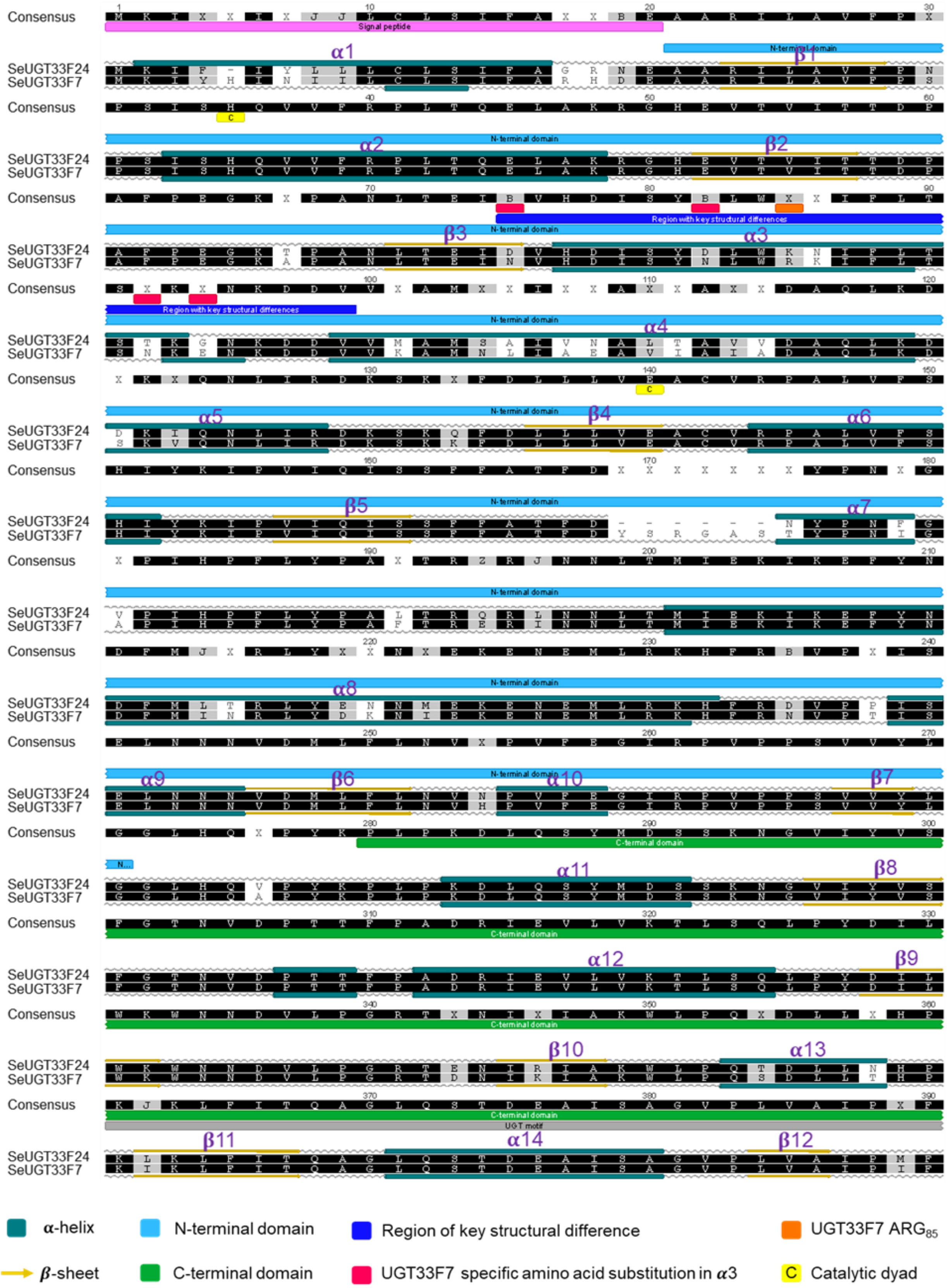
α-helix
β-sheet
N-terminal domain
C-terminal domain
Region of key structural difference
UGT33F7 specific amino acid substitution in α3
UGT33F7 ARG85
Catalytic dyad

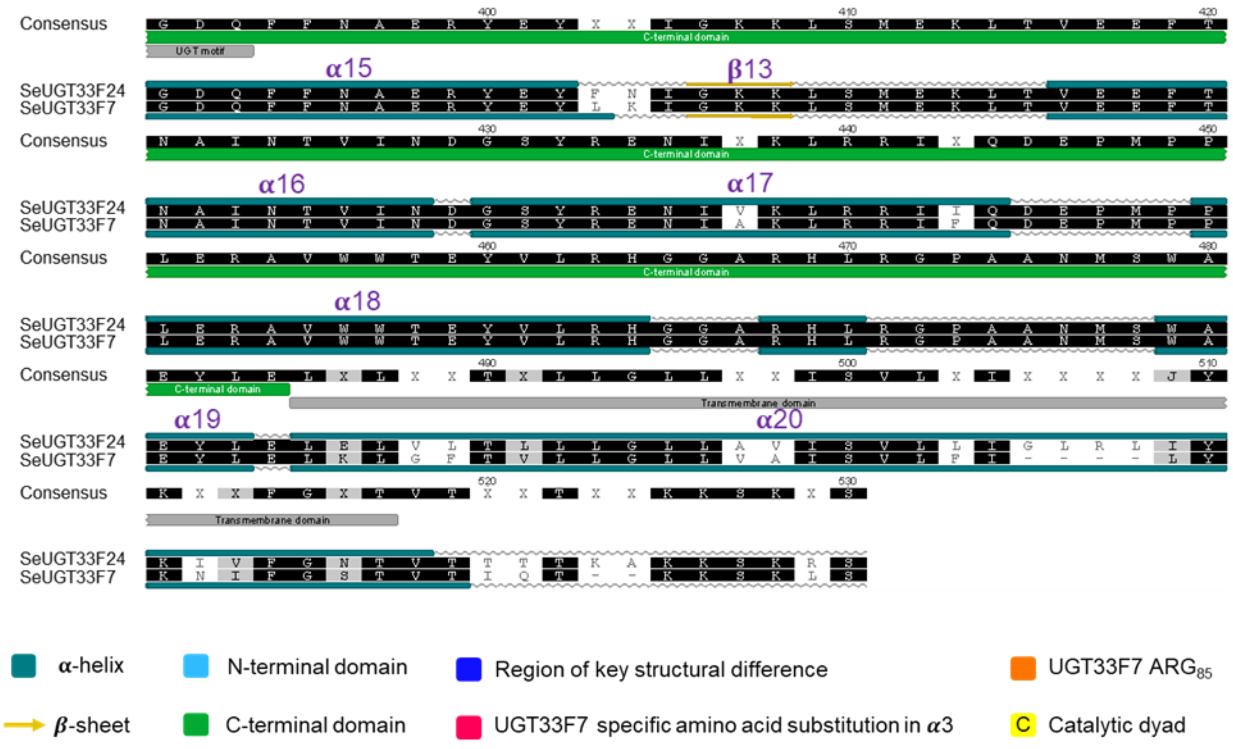

Supplementary Figure 17. Sequence alignment and structure annotation of SeUGT33F24 and SeUGT33F7.

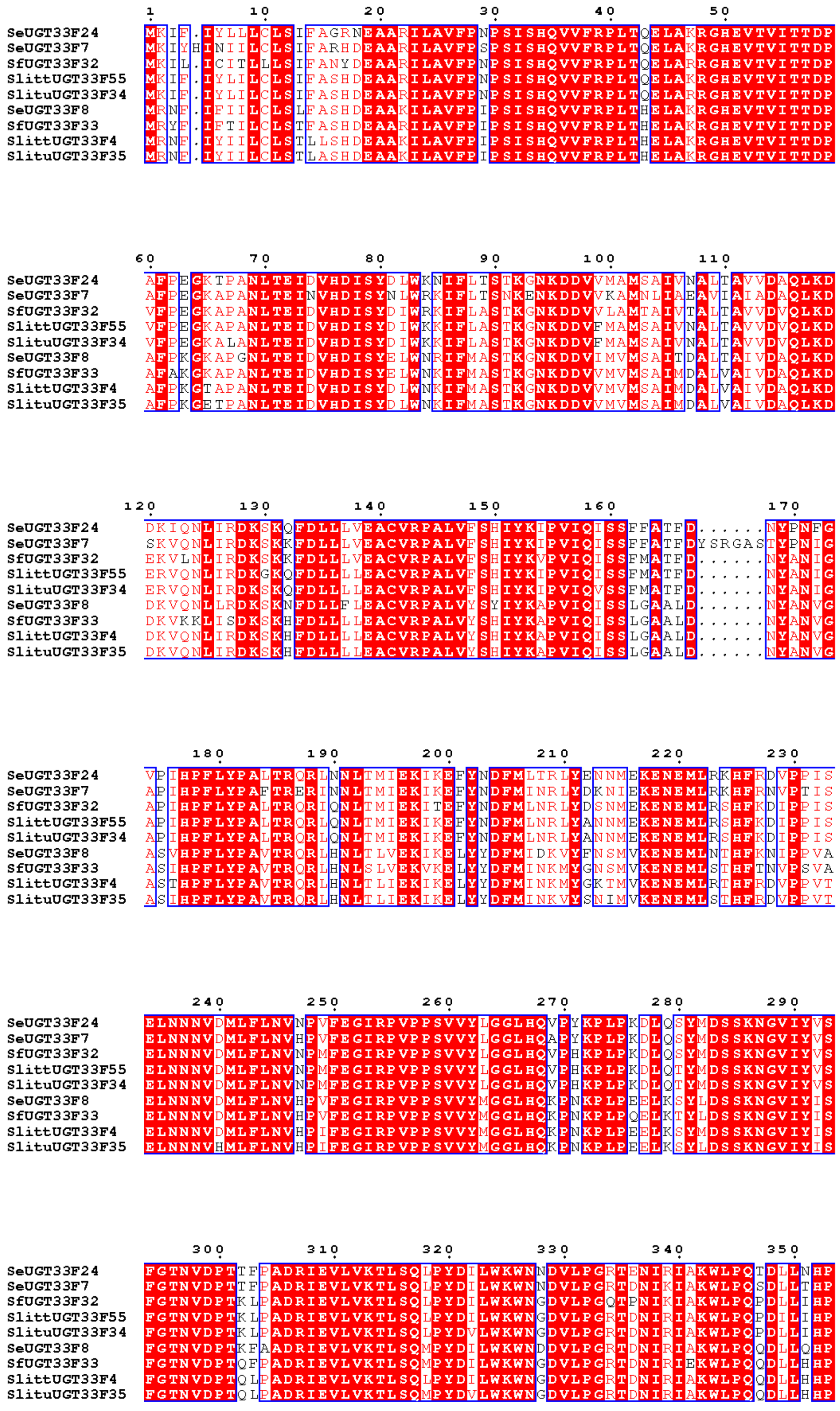
SeUGT33F24
SeUGT33F7
SfUGT33F32
SlittUGT33F55
SlituUGT33F34
SeUGT33F8
SfUGT33F33
SlittUGT33F4
SlituUGT33F35

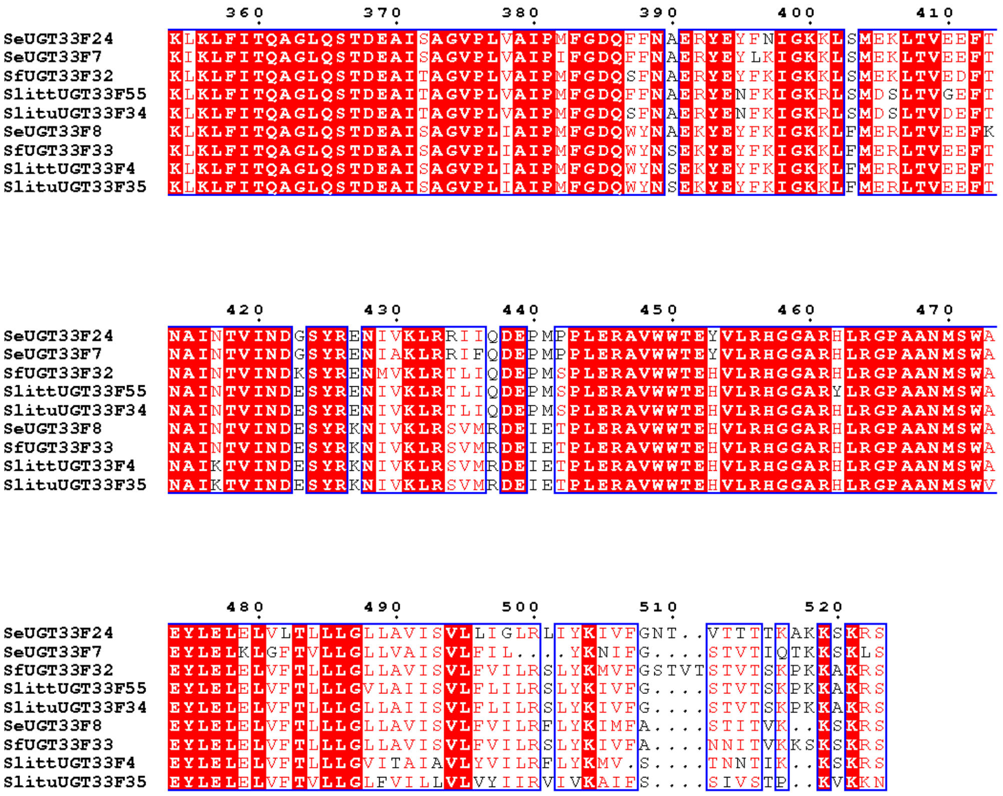

Supplementary Figure 18. Amino acid alignment of nine UGT33F subfamily genes comprising the orthologs and most closely related paralogous genes of SfUGT33F32 in *S. frugiperda*, *S. exigua*, *S. litura* and *S. littoralis*. The alignment was visualized by ESPript 3.0 (35).

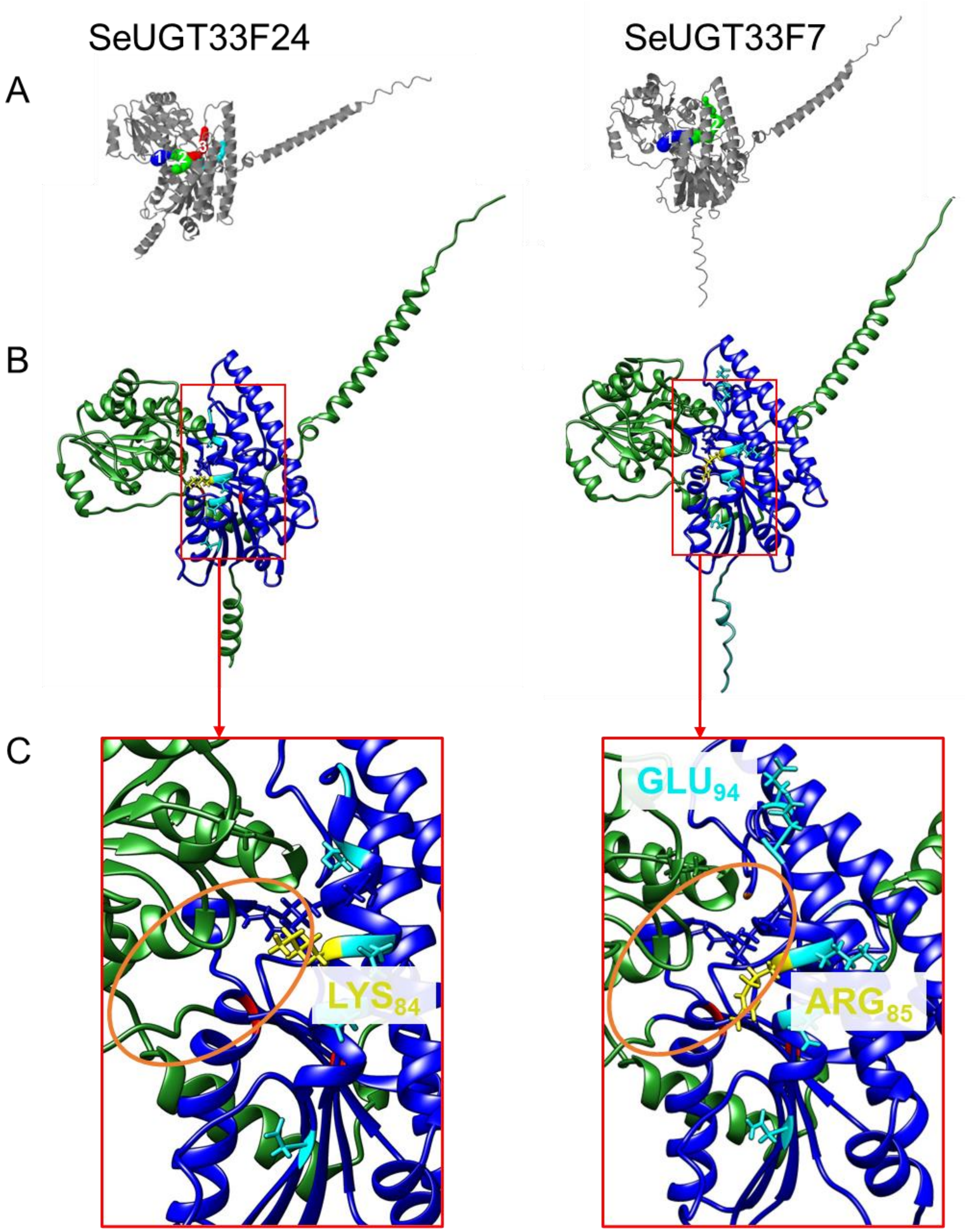

Supplementary Figure 19. Tertiary structures of SeUGT33F24 and SeUGT33F7 and related substrate access tunnels. (A) Position of substrate access tunnels as predicted by CAVER Web v1.2 (1). Tunnel 1 shown in blue, tunnel 2 in green, tunnels 3 and 4 (where present) in red and cyan respectively. Figure created by CAVER Web v1.2 (1). (B) Tertiary structures predicted by AlphaFold (30, 31). N-terminal domain shown in blue and C-terminal in

green. Catalytic dyad coloured in red. (C) Close up of the catalytic region of the tertiary structure. The predicted entrance to substrate tunnel 1 is outlined in orange. Position of the key amino acid substitution in α-helix 3, K84-R85, is shown with side chain visible in yellow. Other substitutions are depicted with side-chains in cyan, with SeUGT33F7 E94 labelled. The six amino acid insertion in SeUGT33F7 (169-174) is coloured yellow. Figures B and C created in UCSF Chimera version 1.16.

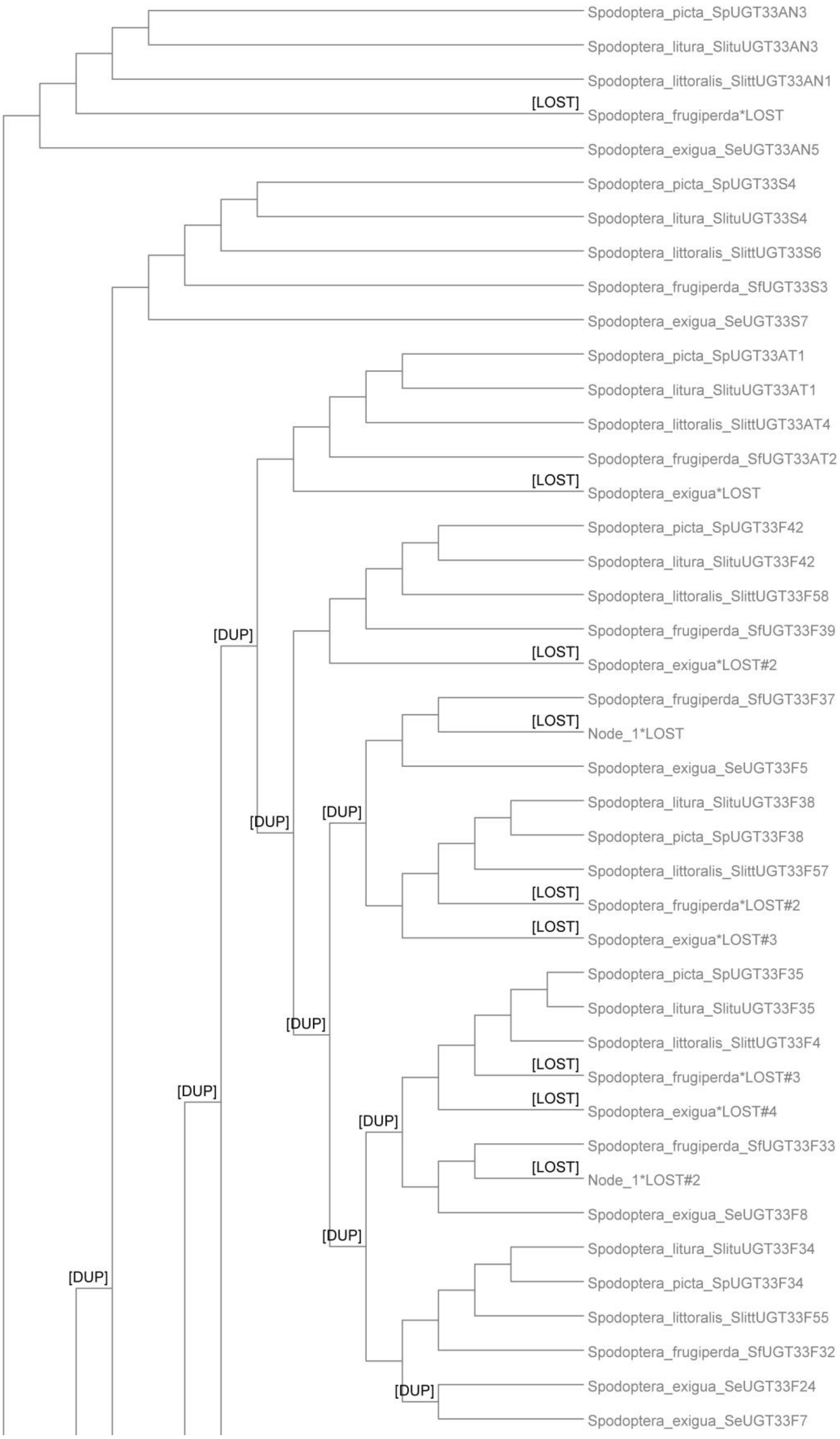
Spodoptera_picta_SpUGT33AN3
Spodoptera_litura_SlituUGT33AN3
Spodoptera_littoralis_SlittUGT33AN1
[LOST]
Spodoptera_frugiperda*LOST
Spodoptera_exigua_SeUGT33AN5
Spodoptera_picta_SpUGT33S4
Spodoptera_litura_SlituUGT33S4
Spodoptera_littoralis_SlittUGT33S6
Spodoptera_frugiperda_SfUGT33S3
Spodoptera_exigua_SeUGT33S7
Spodoptera_picta_SpUGT33AT1
Spodoptera_litura_SlituUGT33AT1
Spodoptera_littoralis_SlittUGT33AT4
Spodoptera_frugiperda_SfUGT33AT2
[LOST]
Spodoptera_exigua*LOST
Spodoptera_picta_SpUGT33F42
Spodoptera_litura_SlituUGT33F42
Spodoptera_littoralis_SlittUGT33F58
[DUP]
Spodoptera_frugiperda_SfUGT33F39
[LOST]
Spodoptera_exigua*LOST#2
Spodoptera_frugiperda_SfUGT33F37
[LOST]
Node_1*LOST
Spodoptera_exigua_SeUGT33F5
Spodoptera_litura_SlituUGT33F38
[DUP]
[DUP]
Spodoptera_picta_SpUGT33F38
Spodoptera_littoralis_SlittUGT33F57
[LOST]
Spodoptera_frugiperda*LOST#2
[LOST]
Spodoptera_exigua*LOST#3
Spodoptera_picta_SpUGT33F35
Spodoptera_litura_SlituUGT33F35
[DUP]
Spodoptera_littoralis_SlittUGT33F4
[LOST]
Spodoptera_frugiperda*LOST#3
[DUP]
[LOST]
Spodoptera_exigua*LOST#4
[DUP]
Spodoptera_frugiperda_SfUGT33F33
[LOST]
Node_1*LOST#2
Spodoptera_exigua_SeUGT33F8
[DUP]
Spodoptera_litura_SlituUGT33F34
[DUP]
Spodoptera_picta_SpUGT33F34
Spodoptera_littoralis_SlittUGT33F55
Spodoptera_frugiperda_SfUGT33F32
[DUP]
Spodoptera_exigua_SeUGT33F24
Spodoptera_exigua_SeUGT33F7

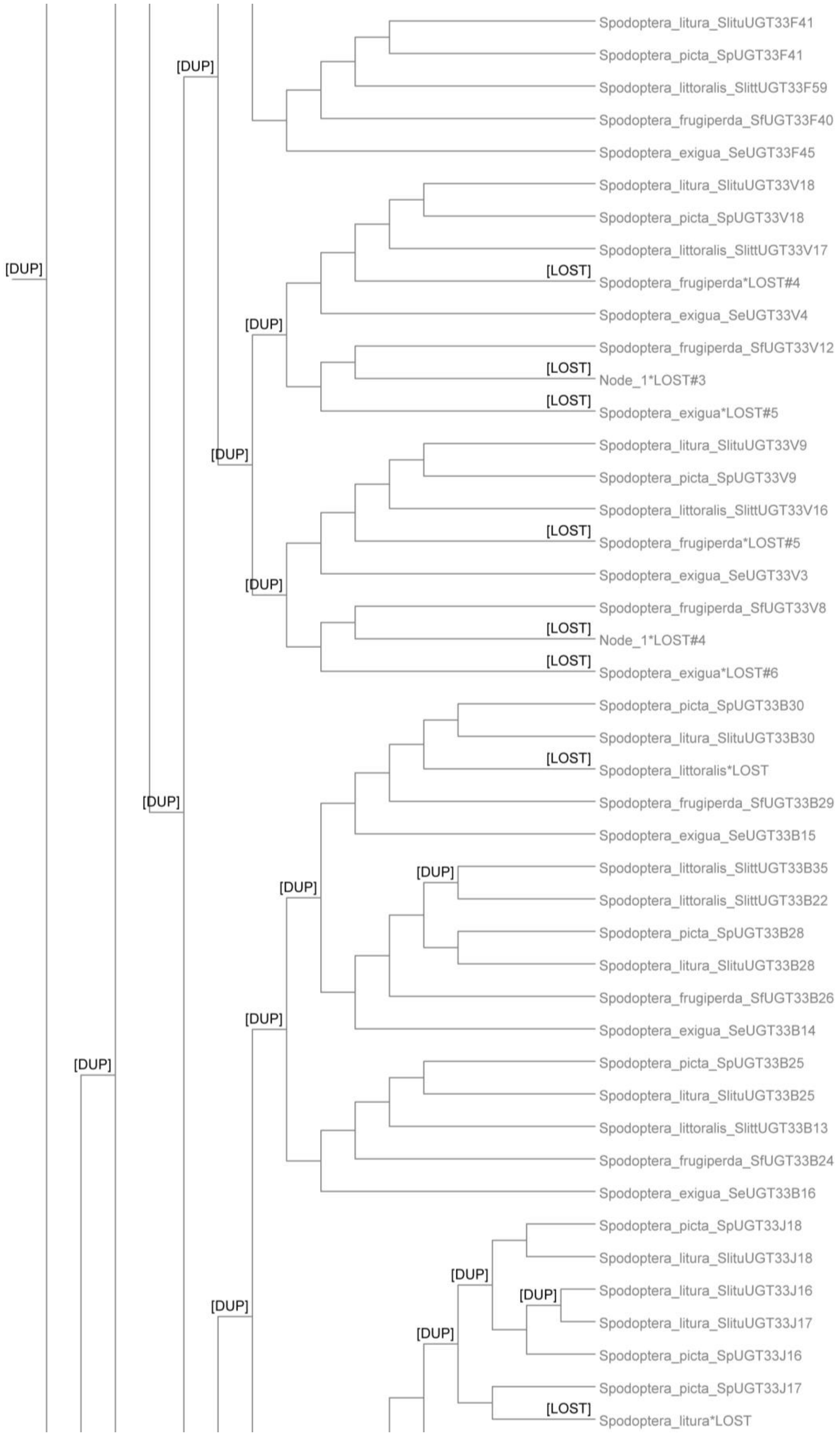
Spodoptera_litura_SlituUGT33F41
Spodoptera_picta_SpUGT33F41
Spodoptera_littoralis_SlittUGT33F59
Spodoptera_frugiperda_SfUGT33F40
Spodoptera_exigua_SeUGT33F45
Spodoptera_litura_SlituUGT33V18
Spodoptera_picta_SpUGT33V18
Spodoptera_littoralis_SlittUGT33V17
Spodoptera_frugiperda*LOST#4
Spodoptera_exigua_SeUGT33V4
Spodoptera_frugiperda_SfUGT33V12
Node_1*LOST#3
Spodoptera_exigua*LOST#5
Spodoptera_litura_SlituUGT33V9
Spodoptera_picta_SpUGT33V9
Spodoptera_littoralis_SlittUGT33V16
Spodoptera_frugiperda*LOST#5
Spodoptera_exigua_SeUGT33V3
Spodoptera_frugiperda_SfUGT33V8
Node_1*LOST#4
Spodoptera_exigua*LOST#6
Spodoptera_picta_SpUGT33B30
Spodoptera_litura_SlituUGT33B30
Spodoptera_littoralis*LOST
Spodoptera_frugiperda_SfUGT33B29
Spodoptera_exigua_SeUGT33B15
Spodoptera_littoralis_SlittUGT33B35
Spodoptera_littoralis_SlittUGT33B22
Spodoptera_picta_SpUGT33B28
Spodoptera_litura_SlituUGT33B28
Spodoptera_frugiperda_SfUGT33B26
Spodoptera_exigua_SeUGT33B14
Spodoptera_picta_SpUGT33B25
Spodoptera_litura_SlituUGT33B25
Spodoptera_littoralis_SlittUGT33B13
Spodoptera_frugiperda_SfUGT33B24
Spodoptera_exigua_SeUGT33B16
Spodoptera_picta_SpUGT33J18
Spodoptera_litura_SlituUGT33J18
Spodoptera_litura_SlituUGT33J16
Spodoptera_litura_SlituUGT33J17
Spodoptera_picta_SpUGT33J16
Spodoptera_picta_SpUGT33J17
Spodoptera_litura*LOST
[DUP]
[LOST]

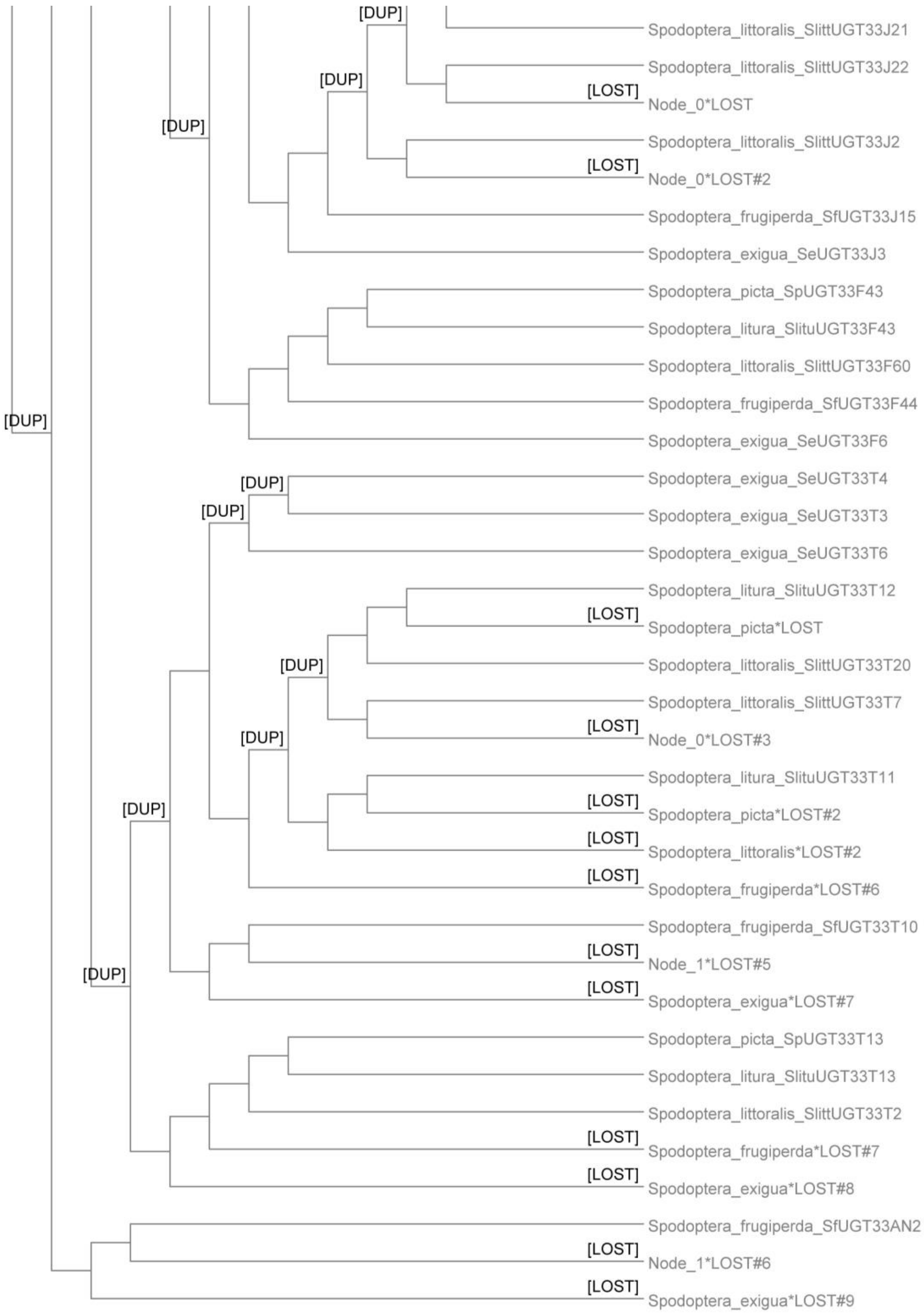

Supplementary Figure 20. Estimation of gene gain and loss events of UGT33 family genes in five *Spodoptera* species by NOTUNG (27) within TBtools (7).

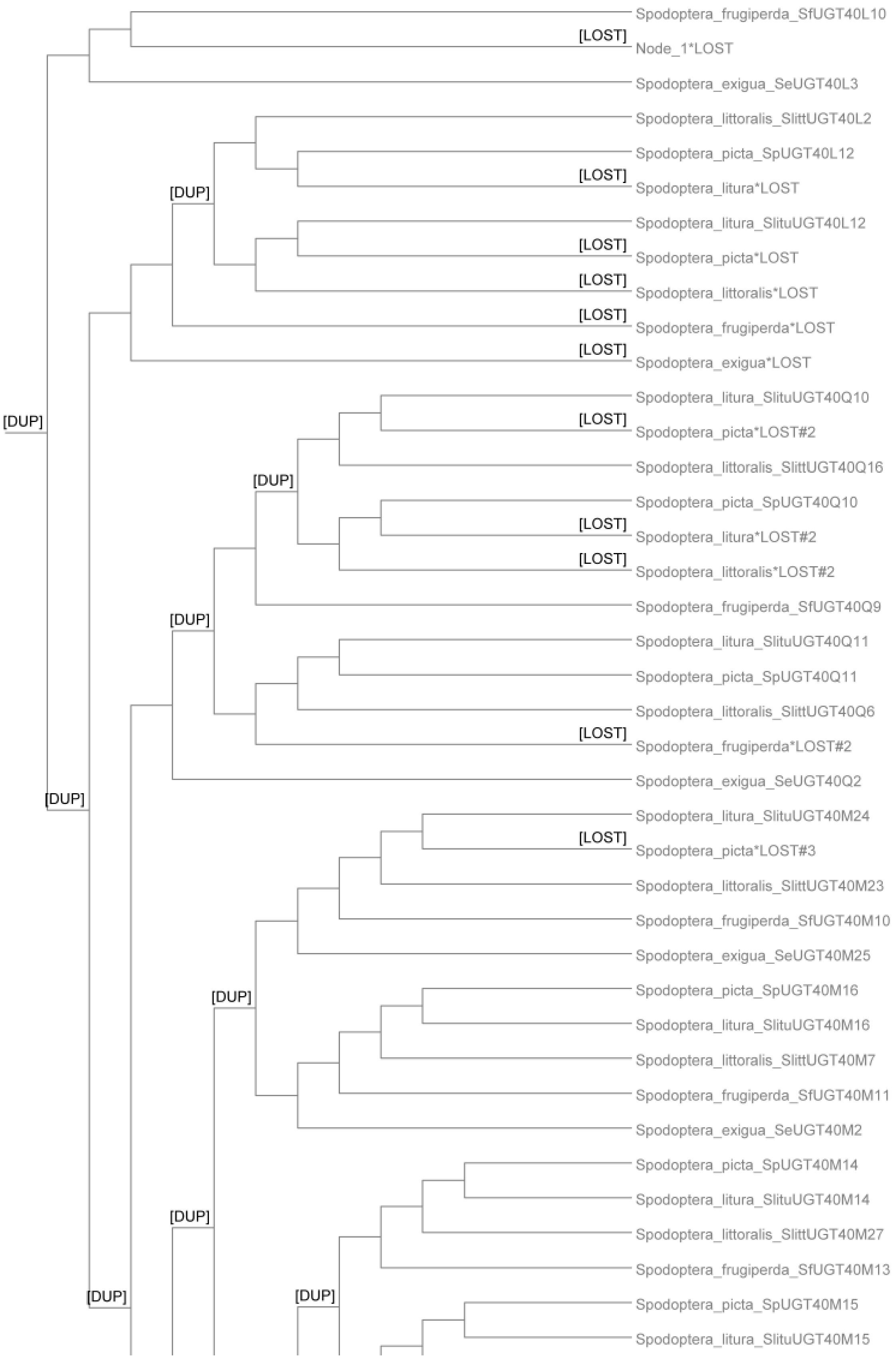
Spodoptera_frugiperda_SfUGT40L10
[LOST]
Node_1*LOST
Spodoptera_exigua_SeUGT40L3
Spodoptera_littoralis_SlittUGT40L2
Spodoptera_picta_SpUGT40L12
[LOST]
Spodoptera_litura*LOST
Spodoptera_litura_SlituUGT40L12
[LOST]
Spodoptera_picta*LOST
[LOST]
Spodoptera_littoralis*LOST
[LOST]
Spodoptera_frugiperda*LOST
[LOST]
Spodoptera_exigua*LOST
Spodoptera_litura_SlituUGT40Q10
[LOST]
Spodoptera_picta*LOST#2
Spodoptera_littoralis_SlittUGT40Q16
Spodoptera_picta_SpUGT40Q10
[LOST]
Spodoptera_litura*LOST#2
[LOST]
Spodoptera_littoralis*LOST#2
Spodoptera_frugiperda_SfUGT40Q9
Spodoptera_litura_SlituUGT40Q11
Spodoptera_picta_SpUGT40Q11
Spodoptera_littoralis_SlittUGT40Q6
[LOST]
Spodoptera_frugiperda*LOST#2
Spodoptera_exigua_SeUGT40Q2
Spodoptera_litura_SlituUGT40M24
[LOST]
Spodoptera_picta*LOST#3
Spodoptera_littoralis_SlittUGT40M23
Spodoptera_frugiperda_SfUGT40M10
Spodoptera_exigua_SeUGT40M25
Spodoptera_picta_SpUGT40M16
Spodoptera_litura_SlituUGT40M16
Spodoptera_littoralis_SlittUGT40M7
Spodoptera_frugiperda_SfUGT40M11
Spodoptera_exigua_SeUGT40M2
Spodoptera_picta_SpUGT40M14
Spodoptera_litura_SlituUGT40M14
Spodoptera_littoralis_SlittUGT40M27
Spodoptera_frugiperda_SfUGT40M13
Spodoptera_picta_SpUGT40M15
Spodoptera_litura_SlituUGT40M15
[DUP]
[DUP]
[DUP]
[DUP]
[DUP]
[DUP]
[DUP]
[DUP]
[DUP]

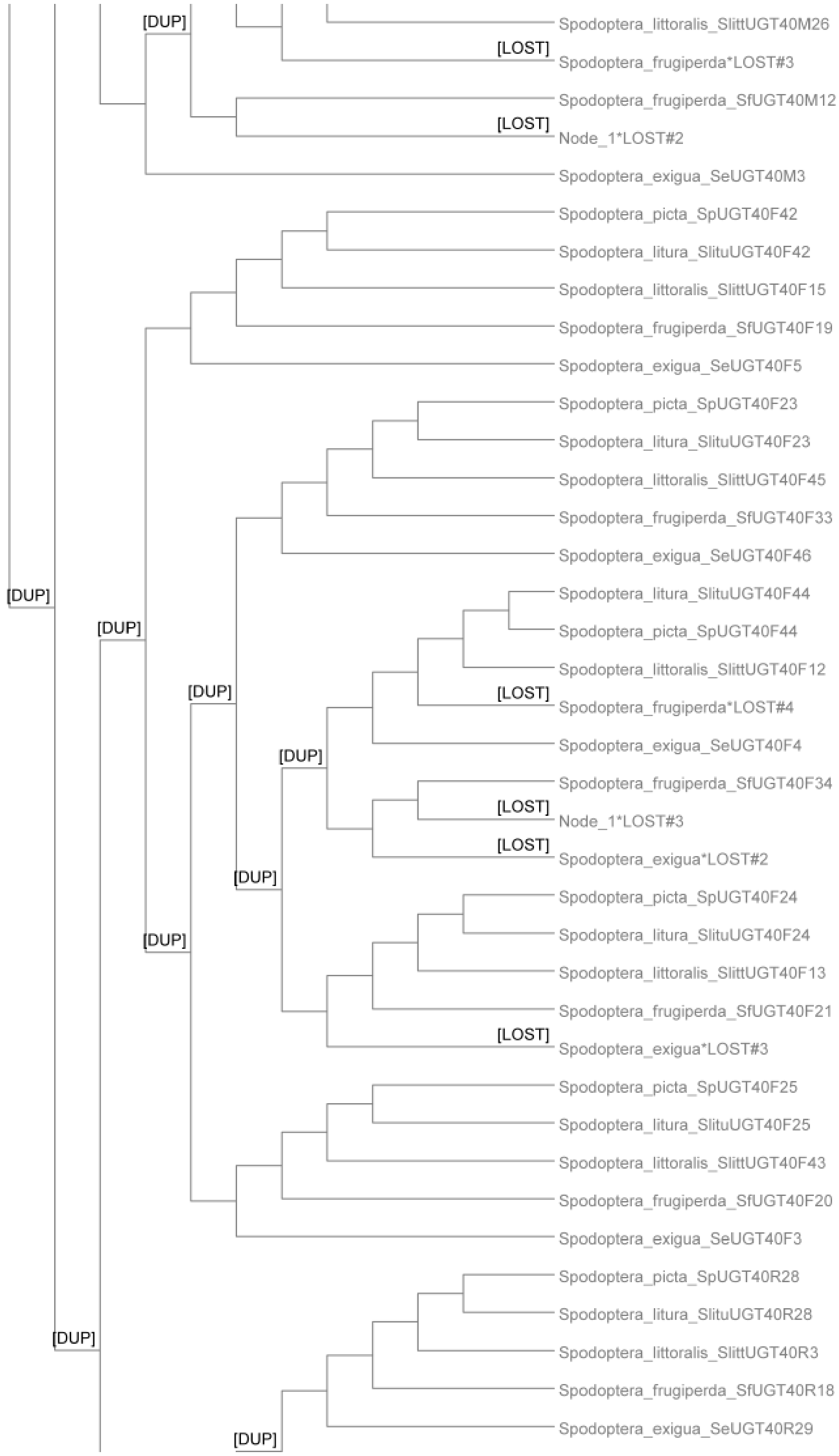
[DUP]
Spodoptera_littoralis_SlittUGT40M26
[LOST]
Spodoptera_frugiperda*LOST#3
Spodoptera_frugiperda_SfUGT40M12
[LOST]
Node_1*LOST#2
Spodoptera_exigua_SeUGT40M3
Spodoptera_picta_SpUGT40F42
Spodoptera_litura_SlituUGT40F42
Spodoptera_littoralis_SlittUGT40F15
Spodoptera_frugiperda_SfUGT40F19
Spodoptera_exigua_SeUGT40F5
Spodoptera_picta_SpUGT40F23
Spodoptera_litura_SlituUGT40F23
Spodoptera_littoralis_SlittUGT40F45
Spodoptera_frugiperda_SfUGT40F33
Spodoptera_exigua_SeUGT40F46
[DUP]
[DUP]
Spodoptera_litura_SlituUGT40F44
Spodoptera_picta_SpUGT40F44
Spodoptera_littoralis_SlittUGT40F12
[DUP]
[LOST]
Spodoptera_frugiperda*LOST#4
Spodoptera_exigua_SeUGT40F4
[DUP]
Spodoptera_frugiperda_SfUGT40F34
[LOST]
Node_1*LOST#3
[LOST]
Spodoptera_exigua*LOST#2
[DUP]
Spodoptera_picta_SpUGT40F24
Spodoptera_litura_SlituUGT40F24
[DUP]
Spodoptera_littoralis_SlittUGT40F13
Spodoptera_frugiperda_SfUGT40F21
[LOST]
Spodoptera_exigua*LOST#3
Spodoptera_picta_SpUGT40F25
Spodoptera_litura_SlituUGT40F25
Spodoptera_littoralis_SlittUGT40F43
Spodoptera_frugiperda_SfUGT40F20
Spodoptera_exigua_SeUGT40F3
Spodoptera_picta_SpUGT40R28
Spodoptera_litura_SlituUGT40R28
[DUP]
Spodoptera_littoralis_SlittUGT40R3
Spodoptera_frugiperda_SfUGT40R18
Spodoptera_exigua_SeUGT40R29
[DUP]

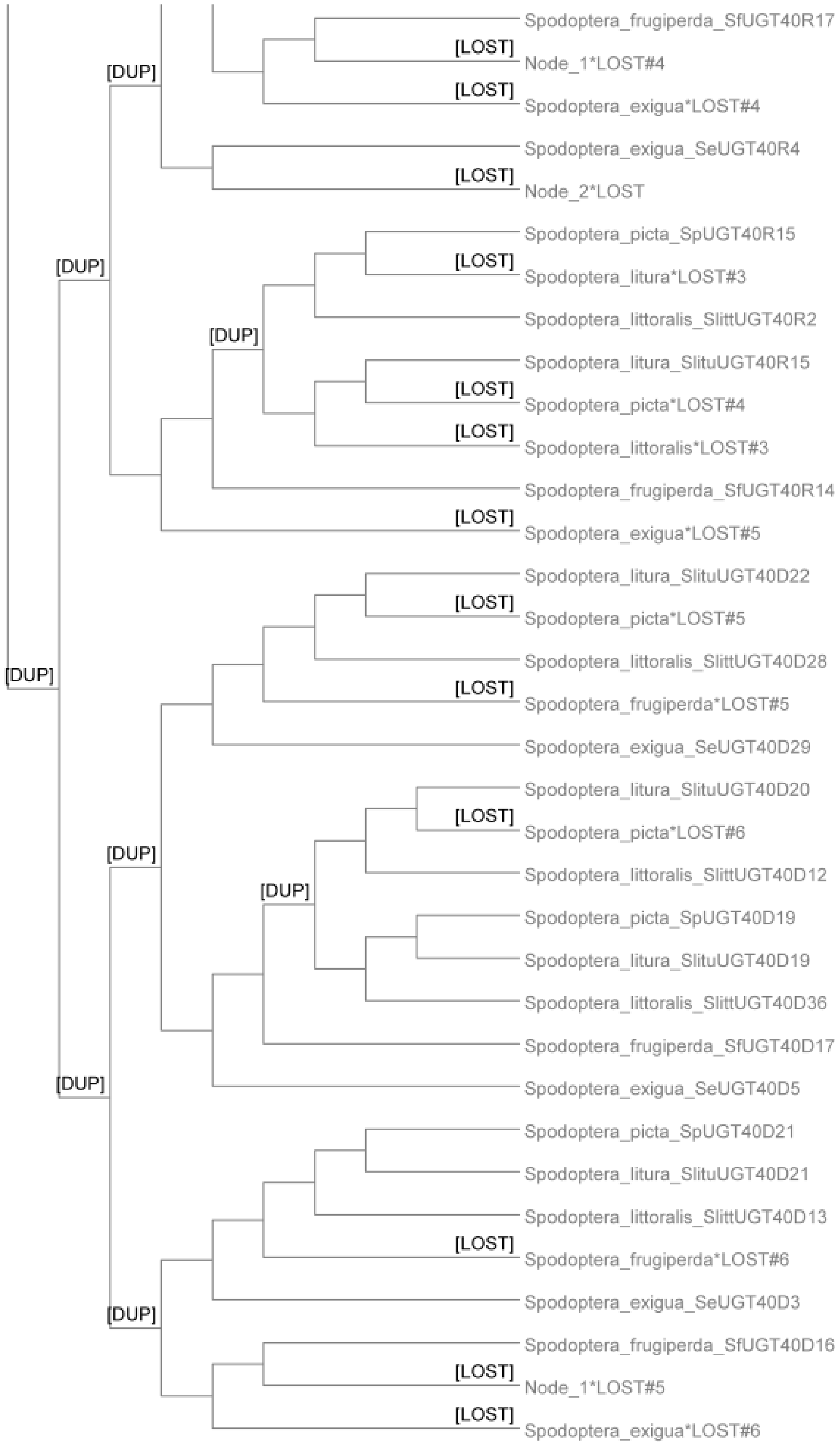

Supplementary Figure 21. Estimation of gene gain and loss events of UGT40 family genes in five *Spodoptera* species by NOTUNG (27) within TBtools (7).

Supplementary Table 1. PCR primers used in this study.

Purpose	Primer name	Sequence (5'-3')
Preparation of template DNAs for sgRNAs	sgRNA-33F44F	TAATACGACTCACTATAGCTAGTTCATGAGTTATAGGA
	sgRNA-33F44R	TTCTAGCTCTAAAACTCCTATAACTCATGAACTAG
	sgRNA-33S3F	TAATACGACTCACTATAGTGGTGACTGATCGATGGTGT
	sgRNA-33S3R	TTCTAGCTCTAAAACACACCATCGATCAGTCACCA
	sgRNA-33B26F	TAATACGACTCACTATAGTAATTCAAGTGTCTTCTCT
	sgRNA-33B26R	TTCTAGCTCTAAAACAGAGAAGACACTTGAATTAC
	sgRNA-33J15F	TAATACGACTCACTATAGTCTAGTAGGAGTTCCAATGT
	sgRNA-33J15R	TTCTAGCTCTAAAACACATTGGAACTCCTACTAGA
	sgRNA-40F33F	TAATACGACTCACTATAGGCAACTCGCAAGTAGCGCT
	sgRNA-40F33R	TTCTAGCTCTAAAACAGCGCTACTTGCGAGTTGCC
	sgRNA-40L10F	TAATACGACTCACTATAGAATCGGAAAGGGCAACTTTG
	sgRNA-40L10R	TTCTAGCTCTAAAACCAAAGTTGCCCTTTCCGATT
	sgRNA-33F32F	TAATACGACTCACTATAGCTTTCCTGAAGGAAAAGCGC
	sgRNA-33F32R	TTCTAGCTCTAAAACGCGCTTTTCCTTCAGGAAAG
	sgRNA-33F24F	TAATACGACTCACTATAGAGCGTCTACAACTGCAGTT
	sgRNA-33F24R	TTCTAGCTCTAAAACAACTGCAGTTGTAGACGCT
Detection of the UGT33c1 cluster deletion	F44F	GCATCTGTCTTCAGCTGTGC
	S3R	CTCCTTTTGGGAAGGCTGGA
	AT2f	GCGCAGCTATCTCACGAAGA
	AT2r	AGAAGGGACTTCAGCTCCAA
	F32f	AGGCCATGAAGTTACCGTCA
	F32r	TCACACATGCTTCCACGAGT
	S3f	GTGARAACATGTTGCTGTGCT
	S3r	CCCCCACTTCTGGCATATCA
Detection of the UGT33c2 cluster deletion	B26 F	CCATCGATCAGCCACCAAGT
	J15 R	TCAAGTTTCACTCCGATTCCA
	B26f	GCGTGGCATCCGTTCCTATA
	B26r	GAGGCACTGGGCGATTGTTA
	B29f	TGCTGATGACCGAAGCTTGT
	B29r	ACTAGGAGGCACTGGACGAT
	B24f	TGGATGCCGTGCAGAAGATT
	B24r	CGATTGCCTTCCCAAATCGG
Detection of the UGT40c cluster deletion	F33F	CTGTGGACGCAGGTGAAAGG
	L10R	AATGGCAAAGTGGTACCAAACC
	F33f	CACTGCCAGAGCAAAGGAAG
	F33r	TTGTTGGTACAGCGGCACTT
	D16f	GTACGCACAAAAAGCGAAGGA
	D16r	AAACTAGGAACAGGATCACGAGG
	M10f	TCAGAAAGCCAAAGAACTCTCCT
	M10r	AAAGGTACGTGGAGTGCTGG
Detection of knockout of single UGT gene	XZ-33F32F	CAGTGTTCCCGAACCCTTCA
	XZ-33F32R	TCACACATGCTTCCACGAGT
	WH-S-33F24F	CCGAAGGTAAAACCCCAGCA
	WH-S-33F24R	ACAACAGAAGGAGGCACTGG
Cloning the cDNA	SfUGT33F44-eF	TCACTAGTCGCGGCCGCTTCAAAATGTCGGCATTACTC

sequences of the UGTs for protein expression	SfUGT33F44-eR	TACCGCATGCCTCGAGTTAATTACGTTTTTTCTTCACAC
	SfUGT33V12-eF	TCACTAGTCGCGGCCGCTTCAAAATGTCGCTCCTATTGTAC
	SfUGT33V12-eR	TACCGCATGCCTCGAGTCAACTCCTTTTAACCTTACCAGC
	SfUGT33V8-eF	TCACTAGTCGCGGCCGCTTCAAAATGTCTCTAGTAAAGCTTT
	SfUGT33V8-eR	TACCGCATGCCTCGAGTCACGCTCGCTTCAATTT
	SfUGT33F40-eF	TCACTAGTCGCGGCCGCTTCAAAATGTCACGTTTTCTCATCA
	SfUGT33F40-eR	TACCGCATGCCTCGAGTCAGCTTTGTTTCAGTTTAATGT
	SfUGT33F39-eF	TCACTAGTCGCGGCCGCTTCAAAATGTTATCTTTTGTGT
	SfUGT33F39-eR	TACCGCATGCCTCGAGTTAATTATTTTTAATTTTAGGAGTA
	SfUGT33F37-eF	TCACTAGTCGCGGCCGCTTCAAAATGATTCTCTTTTTTCA
	SfUGT33F37-eR	TACCGCATGCCTCGAGTCAACTATGTTTAACTTTTTTGA
	SfUGT33F33-eF	TCACTAGTCGCGGCCGCTTCAAAATGAGGTATTTTATCTTT
	SfUGT33F33-eR	TACCGCATGCCTCGAGTCAACTACGTTTTGATTTTGATTTT
	SfUGT33F32-eF	TCACTAGTCGCGGCCGCATGAAGATATTAATTTGTATAACTC
	SfUGT33F32-eR	TACCGCATGCCTCGAGTTAACTACGTTTTGCTTTTTTAG
	SfUGT33S3-eF	TCACTAGTCGCGGCCGCTTCAAAATGTTGCTGTGCTATATAATC AGTGT
	SfUGT33S3-eR	TACCGCATGCCTCGAGTCAGTCTGCCTTAAGCTTCTTTAAA
	SlituUGT33F34-eF	TCACTAGTCGCGGCCGCTTCAAAATGAAGATATTTATCTATTTAA TTCTGTGTCT
	SlituUGT33F34-eR	TACCGCATGCCTCGAGTTAACTACGTTTCGCTTTTTTAGGTTTAC
	SlituUGT33F35-eF	TCACTAGTCGCGGCCGCTTCAAAATGAGGAATTTTATCTATATAA TTCTCTGTCTCAG
	SlituUGT33F35-eR	GTATTGTATCGACACCTAAAGTTAAAAGAATTGACTCGAGGCAT GCGGTA
	SeUGT33F24-eF	TCACTAGTCGCGGCCGCTTCAAAATGAAGATATTTATATATTTATT ACTGTGCCT
	SeUGT33F24-eR	TACCGCATGCCTCGAGTTAACTACGCTTTGATTTTTTAGCTTTAG
	SeUGT33F8-eF	TCACTAGTCGCGGCCGCTTCAAAATGAGAAATTTTATCTTTATAA TTCTGTGTCT
	SeUGT33F8-eR	TACCGCATGCCTCGAG TCAACTACGTTTAGACTTTTTAACAGTG
	SfUGT33F33-eF	TCACTAGTCGCGGCCGCTTCAAAATGAGGTATTTTATCTTT
	SfUGT33F33-eR	TACCGCATGCCTCGAGTCAACTACGTTTTGATTTTGATTTT
	SfUGT33F32-eF	TCACTAGTCGCGGCCGCATGAAGATATTAATTTGTATAACTC
	SfUGT33F32-eR	TACCGCATGCCTCGAGTTAACTACGTTTTGCTTTTTTAG
	SpUGT33F35-eF	TCACTAGTCGCGGCCGCATGAGGAATTTTATCTATATAATTC
	SpUGT33F35-eR	TACCGCATGCCTCGAGTCAGCTACGTTTGGCCTT

Supplementary Table 2. Sensitivity of newly-hatched larvae of different *Spodoptera* strains to DIMBOA.

Species	Strain	LC_{50} (mg/g)	95% FL (mg/g)	Slope±SE	Toxicity ratio
S. frugiperda	XZ	4.62	4.35-4.86	12.16±1.10	-
	XZ-d33c1	0.32	0.28-0.36	7.74±1.28	14.4
	XZ-d33c2	4.48	4.13-4.79	11.01±1.76	1.0
	XZ-d40c	4.81	4.32-5.18	10.63±1.72	1.0
	XZ-33F32	0.28	0.23-0.32	6.32±1.27	16.5
S.exigua	WH-S	2.66	1.74-3.72	5.00±0.55	-
	WH-S-33F24	0.20	0.12-0.35	3.36±0.35	13.3
S. litura	KY	3.82	3.46-4.08	13.40±3.40	-
S. picta	LS	0.24	0.20-0.29	3.42±0.48	-

LC_{50}, lethal concentration that kills 50% of larvae; 95% FL, 95% fiducial limits of LC_{50}; Toxicity ratio was calculated as LC_{50} of background strain/LC_{50} of knockout strain.

Supplementary Table 3. Analysis parameters for substrate detection in UPLC-MS/MS

Analyte	ESI mode	Parent ion (m/z)	Daughter ions (m/z)	Cone voltage (V)	Collision energy (V)
DIMBOA-Glc	ESI-	371.93	148.93*/163.95	38	24/16
1-NA	ESI-	142.88	114.94*/40.94	4	22/22

* quantitative daughter ions.

Supplementary Table 4. Statistics for the *Spodoptera picta* genome assembly

**Assembly Statistic**	**Value**
Assembly size/Total bases	486,557,452 bp
Total number of Contigs	805
Contig N50	2,653,214 bp
Largest contig	11,095,192 bp
Contig L50	54
Mean Length	604,419
Total complete BUSCOs (Arthropoda)	99.1%
Complete single copy BUSCOs	98.4%
Complete single copy BUSCOs (protein)	98.5%
Total gene content	17,448
Total genes annotated	14,578

Supplementary Table 5. Distance matrix showing sequence identity of nine UGT33F family sequences from four *Spodoptera* species. Sequences able to metabolize DIMBOA and those unable to metabolize DIMBOA are bordered in red and blue respectively.

Identity %	SeUGT33F24	SfUGT33F32	SlittUGT33F55	SlituUGT33F34	SeUGT33F7	SeUGT33F8	SfUGT33F33	SlittUGT33F4	SlituUGT33F35
SeUGT33F24		86	88	88	85	79	79	79	77
SfUGT33F32	86		91	92	82	78	77	78	75
SlittUGT33F55	88	91		98	81	80	79	80	78
SlituUGT33F34	88	92	98		81	80	79	79	78
SeUGT33F7	85	82	81	81		75	75	76	74
SeUGT33F8	79	78	80	80	75		92	90	88
SfUGT33F33	79	77	79	79	75	92		91	89
SlittUGT33F4	79	78	80	79	76	90	91		92
SlituUGT33F35	77	75	78	78	74	88	89	92	

Supplementary Table 6. Predicted substrate access tunnels SeUGT33F24

	Bottle neck radius Å	Length Å	Distance to surface Å	Curvature	Throughput	Number of residues	Number of bottlenecks
Tunnel 1	2.1	5.8	5.5	1.1	0.89	27	1
Tunnel 2	1.1	19.6	10.2	1.9	0.55	36	1
Tunnel 3	1.0	27.4	18.0	1.5	0.37	48	1
Tunnel 4	0.9	35.7	24.2	1.5	0.29	58	1

Supplementary Table7. Predicted substrate access tunnels SeUGT33F7

	Bottle neck radius Å	Length Å	Distance to surface Å	Curvature	Throughput	Number of residues	Number of bottlenecks
Tunnel 1	1.9	17.6	12.1	1.5	0.71	44	1
Tunnel 2	0.9	38.8	18.7	2.1	0.17	45	1

Supplementary Table8. Ligand study SeUGT33F24 using DIMBOA

Binding energies dimboa	E_{bound} (Kcal/mol)	E_{max} (Kcal/mol)	$E_{surface}$ (Kcal/mol)	E_{a} (Kcal/mol)	ΔE_{BS} (Kcal/mol)
Tunnel 1	-6.6	-4.9	-6.7	1.8	0.1
Tunnel 2	-6.4	13.3	-3.7	17.0	-2.7
Tunnel 3	-6.5	13.5	2.0	11.5	-8.5
Tunnel 4	-6.1	47.3	-7.0	54.3	0.9

Supplementary Table 9. Ligand study SeUGT33F7 using DIMBOA

Binding energies dimboa	E_{bound} (Kcal/mol)	E_{max} (Kcal/mol)	$E_{surface}$ (Kcal/mol)	E_{a} (Kcal/mol)	ΔE_{BS} (Kcal/mol)
Tunnel 1	-1.3	-5.8	-6.4	0.6	5.1
Tunnel 2	9.0	27.9	12.1	15.8	-3.1

Supplementary Table 10. The specificity-determining positions (SDPs) from nine UGT33F subfamily genes including the orthologs and the most closely related paralogous genes of SfUGT33F32 in *S. frugiperda*, *S. exigua*, *S. litura* and *S. littoralis*.

No	Alignment position	DIMBOA_Active	DIMBOA_No active	Z-score	P-value
1	253	N	H	2	0.01
2	213	L	I	1.97	0.01
3	112	V	I	1.96	0.01
4	29	N	IS	1.39	0
5	275	V	KA	1.38	0
6	442	I	MF	1.38	0
7	190	L	VF	1.36	0
8	446	P	IP	0.96	0
9	222	E	VE	0.96	0
10	447	M	EM	0.96	0
11	409	S	FS	0.96	0
12	209	N	YN	0.96	0
13	181	P	SP	0.96	0
14	43	Q	HQ	0.96	0
15	200	M	LM	0.95	0
16	162	F	LF	0.95	0
17	166	F	LF	0.95	0
18	207	F	LF	0.95	0
19	443	Q	RQ	0.95	0
20	165	T	AT	0.95	0
21	285	Q	KQ	0.95	0
22	88	L	ML	0.95	0
23	269	L	ML	0.95	0
24	101	A	VA	0.95	0
25	63	E	KE	0.95	0
26	433	E	KE	0.95	0
27	148	F	YF	0.95	0
28	394	F	YF	0.95	0
29	283	D	ED	0.95	0
30	1	K	RK	0.95	0
31	215	R	KR	0.95	0
32	398	R	KR	0.95	0
33	238	I	VI	0.94	0
34	298	V	IV	0.94	0
35	384	V	IV	0.94	0
36	60	VA	A	0.87	0
37	115	VA	A	0.87	0
38	378	TS	S	0.87	0
39	82	IL	L	0.86	0
40	353	PT	QS	0.79	0
41	107	NT	DE	0.78	0
42	106	V	MAT	0.78	0
43	163	MF	GF	0.69	0

44	277	HY	NY	0.69	0
45	196	QN	HN	0.69	0
46	84	KR	NR	0.68	0
47	448	SP	TP	0.68	0
48	440	TR	SR	0.68	0
49	230	SK	TK	0.67	0
50	441	LI	VI	0.66	0
51	393	FS	WF	0.5	0
52	412	KS	RK	0.49	0
53	154	IV	AI	0.45	0
54	178	IF	VI	0.44	0
55	288	M	LM	0.4	0
56	234	D	ND	0.39	0
57	324	L	ML	0.39	0
58	22	R	KR	0.39	0
59	396	A	SA	0.39	0
60	255	MV	VI	0.36	0
61	2	I	NIY	0.35	0
62	110	T	VIT	0.34	0
63	282	K	EQK	0.34	0
64	120	ED	DS	0.34	0
65	13	I	TIL	0.33	0
66	528	AS	SV	0.33	0
67	7	LI	IT	0.33	0
68	514	G	ASG	0.27	0
69	239	S	ATS	0.27	0
70	216	L	MVL	0.27	0
71	401	NY	Y	0.23	0
72	411	DE	E	0.22	0
73	47	RK	K	0.22	0
74	121	RK	K	0.22	0
75	407	RK	K	0.22	0
76	357	IN	HQT	0.18	0
77	132	QK	HNK	0.15	0
78	523	ST	IVT	0.11	0
79	235	IV	VI	0.01	0

Supplementary references

1. J. Stourac *et al.*, Caver Web 1.0: identification of tunnels and channels in proteins and analysis of ligand transport. *Nucleic Acids Res.* **47**, W414-W422 (2019).
2. O. V. Kalinina, A. A. Mironov, M. S. Gelfand, A. B. Rakhmaninova, Automated selection of positions determining functional specificity of proteins by comparative analysis of orthologous groups in protein families. *Protein Sci.* **13**, 443-456 (2004).
3. Y. X. Chen *et al.*, SOAPnuke: a MapReduce acceleration-supported software for integrated quality control and preprocessing of high-throughput sequencing data. *Gigascience* **7**, 1-6 (2018).
4. D. Kim, B. Landmead, S. L. Salzberg, HISAT: a fast spliced aligner with low memory requirements. *Nat. Methods* **12**, 357-U121 (2015).
5. B. Langmead, S. L. Salzberg, Fast gapped-read alignment with Bowtie 2. *Nat. Methods* **9**, 357-359 (2012).
6. B. Li, C. N. Dewey, RSEM: accurate transcript quantification from RNA-Seq data with or without a reference genome. *BMC Bioinf.* **12**, 323 (2011).
7. C. Chen *et al.*, TBtools: An Integrative Toolkit Developed for Interactive Analyses of Big Biological Data. *Mol. Plant* **13**, 1194-1202 (2020).
8. M. Kolmogorov, J. Yuan, Y. Lin, P. A. Pevzner, Assembly of long, error-prone reads using repeat graphs. *Nat. Biotechnol.* **37**, 540-546 (2019).
9. B. J. Walker *et al.*, Pilon: an integrated tool for comprehensive microbial variant detection and genome assembly improvement. *PLoS One* **9**, e112963 (2014).
10. F. Kruger, A wrapper around Cutadapt and FastQC to consistently apply adapter and quality trimming to FastQ files, with extra functionality for RRBS data. https://github.com/FelixKrueger/TrimGalore. (accessed 11.4.22).
11. H. Li, R. Durbin, Fast and accurate long-read alignment with Burrows-Wheeler transform. *Bioinformatics* **26**, 589-595 (2010).
12. D. Guan *et al.*, Identifying and removing haplotypic duplication in primary genome assemblies. *Bioinformatics* **36**, 2896-2898 (2020).
13. D. R. Laetsch, M. L. Blaxter, BlobTools: Interrogation of genome assemblies. *F1000 Research* **6**, 1287 (2017).
14. H. Z. Girgis, Red: an intelligent, rapid, accurate tool for detecting repeats de-novo on the genomic scale. *BMC Bioinf.* **16**, 227 (2015).
15. T. Bruna, K. J. Hoff, A. Lomsadze, M. Stanke, M. Borodovsky, BRAKER2: automatic eukaryotic genome annotation with GeneMark-EP+ and AUGUSTUS supported by a protein database. *NAR Genom. Bioinform.* **3**, lqaa108 (2021).
16. Hoff KJ, Lomsadze A, Borodovsky M, S. M, Whole-genome annotation with BRAKER. *Methods Mol. Biol.* 1962,65-95 (2019).
17. D. Kim, B. Langmead, S. L. Salzberg, HISAT: a fast spliced aligner with low memory requirements. *Nat. Methods* **12**, 357-360 (2015).
18. S. Gotz *et al.*, High-throughput functional annotation and data mining with the Blast2GO suite. *Nucleic Acids Res.* **36**, 3420-3435 (2008).
19. F. A. Simao, R. M. Waterhouse, P. Ioannidis, E. V. Kriventseva, E. M. Zdobnov, BUSCO: assessing genome assembly and annotation completeness with single-copy orthologs. *Bioinformatics* **31**, 3210-3212 (2015).
20. R. C. Edgar, MUSCLE: multiple sequence alignment with high accuracy and high throughput. *Nucleic Acids Res.* **32**, 1792-1797 (2004).
21. T. L. Bailey *et al.*, MEME SUITE: tools for motif discovery and searching. *Nucleic Acids Res.* **37**, W202-208 (2009).
22. K. Tamura, G. Stecher, S. Kumar, MEGA11: Molecular Evolutionary Genetics Analysis Version 11. *Mol. Biol. Evol.* **38**, 3022-3027 (2021).
23. J. P. Huelsenbeck, F. Ronquist, MRBAYES: Bayesian inference of phylogenetic trees. *Bioinformatics*

17, 754-755 (2001).
24. S. Q. Le, O. Gascuel, An improved general amino acid replacement matrix. *Mol. Biol. Evol.* **25**, 1307-1320 (2008).
25. G. J. Kergoat *et al.*, A novel reference dated phylogeny for the genus Spodoptera Guenee (Lepidoptera: Noctuidae: Noctuinae): new insights into the evolution of a pest-rich genus. *Mol. Phylogenet. Evol.* **161**, 107161 (2021).
26 D. M. Emms, S. Kelly, OrthoFinder: phylogenetic orthology inference for comparative genomics. *Genome Biol.* **20**, 238 (2019).
27. K. Chen, D. Durand, M. Farach-Colton, NOTUNG: A program for dating gene duplications and optimizing gene family trees. *J. Comput. Biol.* **7**, 429-447 (2000).
28. N. Lam-Tung, H. A. Schmidt, A. von Haeseler, M. Bui Quang, IQ-TREE: A Fast and Effective Stochastic Algorithm for Estimating Maximum-Likelihood Phylogenies. *Mol. Biol. Evol.* **32**, 268-274 (2015).
29. B. Hu *et al.*, GSDS 2.0: an upgraded gene feature visualization server. *Bioinformatics* **31**, 1296-1297 (2015).
30. J. Jumper *et al.*, Highly accurate protein structure prediction with AlphaFold. *Nature* **596**, 583-589 (2021).
31. M. Mirdita *et al.*, ColabFold: making protein folding accessible to all. *Nat. Methods* **19**, 679-682 (2022).
32. C. Colovos, T. O. Yeates, Verification of protein structures: patterns of nonbonded atomic interactions. *Protein Sci.* **2**, 1511-1519 (1993).
33. R. A. Laskowski, J. A. Rullmannn, M. W. MacArthur, R. Kaptein, J. M. Thornton, AQUA and PROCHECK-NMR: programs for checking the quality of protein structures solved by NMR. *J. Biomol. NMR* **8**, 477-486 (1996).
34. E. F. Pettersen *et al.*, UCSF Chimera--a visualization system for exploratory research and analysis. *J. Comput. Chem.* **25**, 1605-1612 (2004).
35. X. Robert & P. Gouet Deciphering key features in protein structures with the new ENDscript server. *Nucleic Acids Res.* **42**,:W320-324 (2014).

Dataset S1: Host plant range of *Spodoptera*

Dataset S2: *Spodoptera frugiperda* UGT sequences

Dataset S3: *Spodoptera* UGT33 and UGT40 sequences

Dataset S4: Sequences for protein expression

Dataset S5: The ortholog groups analysis and duplication events estimated by OrthoFinder

Dataset S6: Performance on plants
